# A cybertaxonomic revision of the micro-landsnail genus *Plectostoma* Adam (Mollusca, Caenogastropoda, Diplommatinidae), from Peninsular Malaysia, Sumatra and Indochina

**DOI:** 10.3897/zookeys.393.6717

**Published:** 2014-03-25

**Authors:** Thor-Seng Liew, Jaap Jan Vermeulen, Mohammad Effendi bin Marzuki, Menno Schilthuizen

**Affiliations:** 1 Naturalis Biodiversity Center, P.O. Box 9517, 2300 RA Leiden, The Netherlands; 2 Institute Biology Leiden, Leiden University, P.O. Box 9516, 2300 RA Leiden, The Netherlands; 3 Institute for Tropical Biology and Conservation, Universiti Malaysia Sabah, Jalan UMS, 88400, Kota Kinabalu, Sabah, Malaysia; 4 Rimba, 4 Jalan 1/9D, 43650, Bandar Baru Bangi, Selangor, Malaysia; 5 jk.artandscience, Lauwerbes 8, 2318 AT, Leiden, The Netherlandss; 6 102, Jalan Muut, Kampung Sekaan Besar, 96250 Matu, Sarawak, Malaysia

**Keywords:** Sundaland, karst habitat, shell, e-taxonomy, tropical, semantic tag, taxonomic impediment

## Abstract

*Plectostoma* is a micro land snail restricted to limestone outcrops in Southeast Asia. *Plectostoma* was previously classified as a subgenus of *Opisthostoma* because of the deviation from regular coiling in many species in both taxa. This paper is the first of a two-part revision of the genus *Plectostoma*, and includes all non-Borneo species. In the present paper, we examined 214 collection samples of 31 species, and obtained 62 references, 290 pictures, and 155 3D-models of 29 *Plectostoma* species and 51 COI sequences of 19 species. To work with such a variety of taxonomic data, and then to represent it in an integrated, scaleable and accessible manner, we adopted up-to-date cybertaxonomic tools. All the taxonomic information, such as references, classification, species descriptions, specimen images, genetic data, and distribution data, were tagged and linked with cyber tools and web servers (e.g. Lifedesks, Google Earth, and Barcoding of Life Database). We elevated *Plectostoma* from subgenus to genus level based on morphological, ecological and genetic evidence. We revised the existing 21 *Plectostoma* species and described 10 new species, namely, *P. dindingensis* **sp. n.**, *P. mengaburensis* **sp. n.**, *P. whitteni* **sp. n.**, *P. kayiani* **sp. n.**, *P. davisoni* **sp. n.**, *P. relauensis* **sp. n.**, *P. kubuensis* **sp. n.**, *P. tohchinyawi* **sp. n.**, *P. tenggekensis* **sp. n.**, and *P. ikanensis* **sp. n.** All the synthesised, semantic-tagged, and linked taxonomic information is made freely and publicly available online.

## Introduction

The purpose of this paper is twofold. Firstly, we demonstrate an updated workflow that uses several free tools to semantically tag and link different types of information during taxonomic revision. This approach allows the taxonomist to manage information in a more effective manner, making good quality data accessible and scaleable, which is essential for the taxonomist himself, future taxonomists and other users. Secondly, we revise the taxonomy of the genus *Plectostoma* based on the materials that have been accumulated since the last revision on the *Plectostoma* species of this region about five decades ago (van Benthem Jutting 1952, 1961). We revised the non-Bornean *Plectostoma* by using the redefined description of shell characters, which are better suited for the representation of the shell ontogeny and shell form. These shell character descriptions constitute a species hypothesis for each species, which is then discussed in terms of its genetic variation and biogeography.

Therefore, in this introduction section, we briefly review current issues in taxonomy, especially on the importance of taxonomic data management. After that, we introduce the taxonomy history of the genus *Plectostoma* and the taxonomic problems of the genus. In the methodology section, we describe in detail the procedure to incorporate cybertaxonomy tools in the taxonomic revision workflow. Finally, we discuss the taxonomy of the genus *Plectostoma* in the results and discussion sections.

### Current issues in taxonomy

Taxonomy is arguably man’s oldest profession and an important fundamental discipline for many other biological fields (Hedgpeth 1961, Wilson 2004). It helps us to inventory the biodiversity on earth by naming each classified group of organisms that shares certain attributes based on, for example, genetic and morphological evidence. However, the taxa names are not necessarily maintained in perpetuity, as classifications can be changed when more specimens and novel attributes are examined and compared. In fact, the continuous improvement of the existing classification scheme of any taxon is a fundamental characteristic in taxonomic science. As a result, a taxon name is a summary of hundreds of years’ of taxonomists’ attempts to classify biodiversity.

Despite this long history, only a small fraction of biodiversity has been named (Mora et al. 2011). Certainly, more resources (money, time and taxonomists) are needed to describe the remaining unknown biodiversity. For example, Carbayo and Marques (2011) estimated that over two hundred billion US dollars are needed to describe the remaining ca. 5.5 million undescribed species. They indicate that half of the cost is to be invested on training taxonomists and project budgets. Although it is not being discussed in Carbayo and Marques (2011), it is not hard to imaging that a significant proportion of the amount were spent on travelling to various museums for studying type specimens and other samples, compiling literature and maintaining all this information. Even if there were no financial constraint, given the current pace in describing species (ca. 18000 species per year, see Zhang 2008), about 400 years are still needed to describe all the species, which translates into 10 generations of taxonomists (Padial et al. 2010).

All organisms could eventually be scientifically described, but there are several pressing contemporary issues that could affect the advancement of taxonomy as a science. Firstly, the population of taxonomists is declining (Wägele et al. 2011), which will directly reduce the pace of species discovery and capacity building for future taxonomists. Secondly, the majority of taxonomic resources (e.g. type materials, literature, expertise) are centralised in developed countries, whereas the remaining undescribed species are concentrated in developing countries (Rodrigues et al. 2010). Neither taxonomists from developed countries nor taxonomists from developing countries can work effectively without having both biodiversity and taxonomic resources readily available to be integrated. Thirdly, many unnamed species are likely to go extinct under the current rates of habitat destruction in biodiversity hotspots (Giam et al. 2011). Thus, taxonomists have to find ways to be able to work more effectively and accelerate the pace of species discovery.

Several proposals to improve the practice of taxonomy have been published (e.g. Webb et al. 2010, Wägele et al. 2011, Miller et al. 2012). One of these suggests that taxonomists embrace the information and internet technologies in their routine workflow. This is particularly relevant because taxonomy is a data-rich science and taxonomists have to deal with all past taxonomic works, in terms of literature, collection data, genetic data, and ecology, that have been accumulated over hundreds of years. Thus, computer and internet technologies have become important tools to manage all these taxonomic data, especially regarding data storage, dissemination, and retrieval.

Over the past few years, many web-based databases have been developed to accommodate almost all kinds of the data that taxonomists deal with. For example, museum specimen data are held in the Global Biodiversity Information Facility (http://www.gbif.org, Edwards 2004), morphology data (images) in Morphbank (http://www.morphbank.net), genetic data in the Barcoding of Life Database (http://www.barcodinglife.com, Ratnasingham and Hebert 2007) and GenBank (http://www.ncbi.nlm.nih.gov/genbank, Benson et al. 1997), nomenclature data in ZooBank (http://zoobank.org, Pyle and Michel 2008), and literature in the Biodiversity Heritage Library (http://www.biodiversitylibrary.org, Gwinn and Rinaldo 2009). Although the need to use these facilities to improve taxonomy has been emphasised (e.g. Maddison et al. 2012, Wheeler et al. 2012, Miller et al. 2013), the majority of taxonomists has yet to join this movement. This hesitance may be caused by taxonomists’ fear for investing extra work to supply the data, and their doubt of the usefulness of these poorly integrated databases.

Recently, tools have become available for managing and integrating various types of taxonomic data by using either top-down approaches (e.g. Encyclopedia of Life http://eol.org, Wilson 2003) or bottom-up approaches (e.g. Lifedesks http://www.lifedesks.org, offered by EOL; Scratchpads http://scratchpads.eu, Smith et al. 2009). The top-down mechanism acts as an automatic data aggregator that harvests and pools semantic-tagged information from different databases. The bottom-up mechanism, on the other hand, acts as quality controller that checks, links and tags the different types of taxonomic data. Obviously, the key factor that determines the success of cybertaxonomy and the main challenge of these integration processes is the quality of the underlying taxonomic data (Parr et al. 2012).

Many data in the databases are outdated (e.g. nomenclature change), incorrect due to the limitation of technology (e.g. automation text extraction, Page 2011) or human error (e.g. misidentification, Yesson et al. 2007), and not linked or semantic-tagged. In fact, taxonomists spend much of the time in their careers to validate, link and tag all the existing and new taxonomic information. Thus, their contribution to the quality control of the data is essential. However, each database has its unique needs, standards, and format for the data and thus it might require taxonomists to do redundant work, such as uploading and key in the same data in different formats as required by the database.

In addition to the possible redundant efforts that need to be spent, it is not very clear how taxonomists as data suppliers would benefit most from such databases. In view of this, we demonstrate a working example of how these existing databases and platforms can be incorporated into revisionary taxonomic study processes, which begin with managing new specimen information, then establishing identity (literature study and examination of specimens in museum collections), and then writing taxonomic treatises (Winston 1999). Our working example consists of a revision of the taxonomy of the genus *Plectostoma* of Peninsular Malaysia, Indochina and Sumatra (i.e. non-Bornean) by using cybertaxonomy tools during the entire revisionary taxonomy process. With these cybertaxonomy tools, we show that various kinds of taxonomic information can be semantically tagged, linked and integrated in a more user-friendly interface (Penev et al. 2011). In addition, we demonstrate that using this technology and these databases could facilitate the hypothesis-testing nature of taxonomic research that deals with vast amounts of different kinds of information (e.g. Sluys 2013). The validated, linked and tagged data that are generated during the taxonomic revision in turn facilitates forthcoming taxonomy studies that will be conducted by future taxonomists. Finally, all this species information is made readily available and accessible for other users such as ecologists and conservationists, especially for those who have limited budget and are from developing countries.

### An overview of genus *Plectostoma*’s taxonomy history

The study of *Opisthostoma* sensu stricto and *Plectostoma* started during the English colonisation in India and North Borneo in the 1860s (Blanford and Blanford 1860; Adam 1865b). All early collections were made by English officers, who later described the species themselves or sent their collections to other malacologists for species identification and publication. Until 1880, there was only one *Plectostoma* species known–*Plectostoma decrespignyi* from Labuan, North Borneo. Between the 1880s and 1900s, A. H. Everett, C. Hose and S. Beddome collected more *Plectostoma* specimens from North Borneo and Sarawak, where they were working as either British colonial administrators or naturalists. Based on these specimens, a total of 20 *Plectostoma* species were eventually described from Borneo (Godwin-Austen 1889, 1890, Boettger 1893, Smith 1893b, 1894, 1904, 1905a, Fulton 1901). Many of these were originally described under *Plectostoma*, which was considered a subgenus of *Opisthostoma*.

After the intensive malacological documentation in Borneo in the late 19th and early 20^th^ century, more material of *Plectostoma* was collected in Sarawak and Sabah during explorations which principally had a different purpose, by the geologist G.E. Wilford and his associates in 1960s, by soil scientists K. Auffenberg and D.K. Dorman in the late 1980s, and by the botanist J.J. Vermeulen in the 1980s and early 1990s. All of this material was revised by Vermeulen (1994), under the genus *Opisthostoma*, which resulted in the description of 27 new *Plectostoma* species from Borneo. Since the 2000s, *Plectostoma* has attracted more interest, also regarding its phylogeny and evolution (Schilthuizen 2003, Schilthuizen et al. 2006, Webster et al. 2012) and ecology (Schilthuizen et al. 2003a, 2005).

In addition to the Bornean *Plectostoma*, two other *Plectostoma* species were described from Southern Thailand and Southern Vietnam in the 1900s (Sykes 1903, Dautzenberg and Fischer 1905). Between the 1930s and 1960s, M.W.F. Tweedie (then director of the Raffles Museum of Singapore) collected many *Plectostoma* specimens during explorations of Peninsular Malaysia. These specimens were later described as 13 new *Plectostoma* species (Tomlin 1938, 1948, van Benthem Jutting 1952, 1961). The research on the taxonomy of non-Bornean *Plectostoma* ceased in the 1960s with the retirement of Tweedie and the death of van Benthem Jutting. However, ecological investigations of one *Plectostoma* species–*Plectostoma retrovertens*–had started in 1960s by A.J. Berry, a professor at University Malaya (Berry 1961, 1962, 1963, 1964, 1966).

No publications on *Plectostoma* from Peninsula Malaysia appeared between 1966 and 1996, but many *Plectostoma* specimens were collected by Geoffrey W. H. Davison, mainly from the state of Kelantan, and other localities in Malaysia (e.g. Davison and Kiew 1990). In 1996, *Plectostoma klongsangensis* was described from Southern Thailand (Panha 1996). After that, four new species were described from Southern Thailand and Peninsula Malaysia (Maassen 2001). One year later, the first *Plectostoma* from Sumatra–*Plectostoma kitteli*, was decribed by Maassen (2002).

To sum up, a total of 69 *Plectostoma* are currently known. The hotspots of *Plectostoma* diversity are the Malay Peninsula (including the southern part of Thailand) and Borneo, harbouring 19 and 48 species, respectively. In addition, one species is known from Sumatra and another from Southern Vietnam.

### Current taxonomic status of *Plectostoma* from Indochina, Sumatra and Peninsular Malaysia

The last revision of the non-Bornean *Plectostoma* species was done by van Benthem Jutting (1952, 1961). It is important to note that she described 10 new species on the basis of shell characters from only 21 samples from 16 locations. However, it is problematic to use conventional shell descriptions in this genus. The striking shell form of *Plectostoma* has attracted the attention of malacologists, but it also poses a challenge to describe the shell accurately. As mentioned by Benthem-Jutting (1952), “… it is evident that in such irregular shells … the measurements can only be given approximately, and never indicate the real proportion of the shell…” and “…after comparing over and over again did I succeed in checking the points of difference [between species], but even then it remained difficult to bring the true nature of these minor details into adequate words”. To date, Vermeulen’s (1994) approach in describing the *Plectostoma* shell is the most comprehensive, but it is still difficult to recognise the species from the written description alone.

As mentioned in the taxonomic history section above, additional *Plectostoma* specimens were collected by Geoffrey W. H. Davison in Peninsular Malaysia in the 1990s. Furthermore, we collected more specimens, including living ones for genetic study, during field trips to Peninsular Malaysian limestone hills between 2010 and 2011. These *Plectostoma* specimens are valuable to improve the taxonomy of *Plectostoma* in Peninsular Malaysia. Thus, it is timely to revise the non-Bornean *Plectostoma* based on these recently collected specimens by re-examining the species hypotheses formed on the basis of shell morphology. In addition, we also update the knowledge of *Plectostoma* regarding conservation status, distribution, and genetics.

## Materials and methods

### Taxonomic data mining, storing and tagging (Appendix 1)

**Literature.** In addition to traditional searching of, for example, the Zoological Record, we also searched for the terms “Geothauma, Plectostoma and Opisthostoma” in Google Scholar (on 21^st^ November 2012) and the Biodiversity Heritage Library (on 19^th^ November 2012). A URL link to the full-text article was provided as listed in bibliography whenever possible (e.g. from http://www.biodiversitylibrary.org). Each of the articles was tagged with the relevant *Plectostoma* taxon names. All the relevant references were catalogued as individual contents in bibliography of the *Opisthostoma* Lifedesks pages (http://opisthostoma.lifedesks.org/biblio). All the relevant bibliography metadata of each article were entered and stored according to the standard data entries of BibTeX (http://www.bibtex.org/) as implemented in Lifedesks. Furthermore, the bibliography metadata can be exported as BibTeX formatted file (.bib), which all the tagged metadata can be retrieved and reused. For video tutorials, see Appendix 1(1 and 2).

**Nomenclature information and classification.** After the relevant literature was identified, the relevant taxon names were extracted and organised by using the classification tool (tree editor) of Lifedesks (for detailed descriptions of the methodology, see: http://help.lifedesks.org/classification/edit). The classification and nomenclature information can be downloaded and saved according to the DarwinCore standard as xml file. For video tutorials, see Appendix 1(3).

**Species information.** In addition, the original species descriptions and important notes from the literature were imported to individual species pages as quotations (for detailed descriptions of the methodology, see: http://help.lifedesks.org/quickstart). New and unpublished data, together with the information extracted from literature were managed and stored in the relevant chapters under the headings of ‘Overview’, ‘Conservation’, ‘Description’, ‘Ecology and Distribution’, ‘Evolution and Systematics’, and ‘Relevance’ of each species webpage. As all the information was tagged accordingly in the form of xhtml format, the data of each species can be retrieved and reused. For video tutorials, see Appendix 1(4).

**Managing museum collections data.** As common curation practice, each collection (i.e. one museum lot) consists of a specimen or multiple specimens, which are kept either dry (empty shells) or wet (shells with animals preserved in ethanol) that were collected at a single sampling occasion, for example from a particular location at a particular day/time. In this study, each collection is regarded as a sample. For each sample, there are two categories of information that can be extracted, namely the physical properties and metadata of the samples.

For the physical properties of the samples, we recorded the exact number of shells or (for samples >10 shells) categorised the samples into four categories of sample size: 1) > 10 (10–24 shells); 2) > 25 (25–49 shells); 3) > 50 (50–100 shells); and 4) > 100 (>100 shells). We also obtained shell morphology data as follows. Whenever possible, each registered collection was photographed. The images for each unique collection were imported into Lifedesks as an individual content. Each of the images was then linked with the species name. For video tutorials, see Appendix 1(5).

Metadata consisted of collection reference number, collector information, collecting date, and locality of each sample. This information was published in Lifedesks as image description, except for the collection reference number, which was published as image caption. In addition to presenting collection data in a tabular format (Appendix 2), we also published the collection data in a more interactive manner, which can be used in Google Earth.

Whenever possible, location data of the collections were georeferenced. When the location description in the specimen label or in the publication was not clear, the itinerary of the collectors or expedition which had been published in other types of publication (e.g. maps and reports) was consulted. After the exact limestone hill where the collection had been made was identified, it was verified in Google Earth, after which the latitude and longitude were obtained. Although the coordinates as obtained from Google Earth are of high accuracy, they might be too accurate and lack an uncertainty estimate (Mesibov 2012). Thus, the coordinates that we report here should be interpreted as the location of the limestone hill and not the exact spot where the specimens were collected.

A Keyhole Markup Language (KML) file, which comprises the location, images, and collection reference number of each of the museum collections, was created by using Google Earth Spreadsheet Mapper v3.0 (http://www.google.com/earth/outreach/tutorials/spreadsheet3.html). For the data input for the spreadsheet (Template4), the species name was used for “Folder name”, and collection reference number for “Placemark Name”; the concatenation of species name and collection reference number for “Title”; URL of each collection’s original image in Lifedesks was named “Image URL”; detailed collection data as “Paragraph Text”. After that, the data in Spreadsheet Mapper were converted into a KML file, which allows semantic-tagged collection information to be retrieved and reused. When the KML file is opened in Google Earth, each of the museum collection (specimens) is shown as a single landmark on the virtual earth. For video tutorials, see Appendix 1(6).

### Specimen repositories

BMNH Natural History Museum (previously known as British Museum (Natural History), London, United Kingdom.

BOR BORNEENSIS collection, Institute for Tropical Biology and Conservation, Universiti Malaysia Sabah, Kota Kinabalu, Malaysia.

RMNH Naturalis Biodiversity Center (previously known as Rijksmuseum van Natuurlijke Historie), Leiden, the Netherlands

V Jaap Vermeulen’s private collection, Leiden, the Netherlands.

YSC Chen Yansen’s private collection, Medan, Sumatra, Indonesia.

ZMA Naturalis Biodiversity Center (previously known as Zoological Museum of Amsterdam), Leiden, the Netherlands.

### Establishing identity and revising the collection data

In the routine of conventional taxonomic revision practice, samples are sorted into groups (e.g. morphospecies) based on their morphology and distribution. Then, each group is assigned to an existing species name–when the morphology, distribution and/or other important characteristics fit with the species’ morphological description and distributional range as mentioned in the literature; or a newly designed species name–when the characteristics do not fit to any of the named species. In some cases, different species identities may have been assigned to the same specimen by multiple taxonomists. Thus, for each specimen, the collection data (morphology, distribution, genetics and others) are immutable, but the species name is mutable.

The key of this process is the taxonomists, who gather, integrate, sort and analyse, not only the biological specimens, but diverse and vast amounts of information from hundreds of specimens (Sluys 2013). This task has become more challenging for taxonomists and their successors because of the accumulative nature of taxonomic information. Thus, taxonomists have been using information technology to assist their routine work since the 1980s (Heywood 1974, Maxted 1992). However, the potential of information technology to be used by taxonomists remains underexploited, except for data storage. In fact, in additional to data storage, this technology can improve the efficiency and effectiveness of taxonomists in integrating, sorting, analysing and disseminating the information from the specimens.

As mentioned above, all key information from specimens and literature was digitised and tagged. Here, we integrated different types of information for different processes in taxonomy revision. For the specimen sorting process, we used a KML file to link and present the unique collection number, images and location data for each specimen. Each museum collection was shown on Google Earth as a landmark and these landmarks were sorted into respective species folders. When each landmark was selected (by clicking it), the information of the morphology (as shown in images), location (text description and map), and other relevant information was visible to the user (taxonomist). Species identification can be done in a single platform (i.e. Google Earth), where the morphological variation within a species or between the specimens across the genus’ geographical range can be examined. Based on this information, the species identity of the landmark (specimen) was determined by either keeping the landmark in the same species folder or moving to the other species folder. Likewise, whenever the coordinate of a specimen location was wrong, the landmark was edited by moving it to the correct location. Whenever necessary, the specimen itself was examined. For video tutorials, see Appendix 1(7).

After all the collection species identifications and location data in the KML file were verified and corrected, the data in the KML file were extracted with a customised Python script to update the data in Spreadsheet mapper and image species link in Lifedesks for all specimens in an automatic manner. This saved much time compared to the traditional laborious method of manually updating the collection database specimen by specimen after sorting and identification. Lastly, the taxonomic content was written in Lifedesks and then exported to the appropriate format and layout for publication. For video tutorials, see Appendix 1(8).

Since the information was stored and tagged digitally, a simple program can be customised to retrieve, integrate and process the data from many different online/offline databases and files. In our case, we used scripting language Python 2.73 (http://www.python.org). Its “urllib” and “re” modules were used to retrieve information from Internet resources and searching for patterns in text. In the same way, additional specimen information from Lifedesks (i.e. image pages) can be integrated into the KML file. Simultaneously, the literature and species information compiled in Lifedesks can be retrieved easily when necessary.

### Species delimitation

The application of a species concept in *Plectostoma* has been particular problematic. Nowadays, the most widely accepted species concepts (e.g., the biological species concept; Mayr 1942) include some aspect of genetic and/or reproductive cohesion. Such species concepts are, however, difficult to apply in taxa like *Plectostoma*, where all species and populations are restricted to isolated limestone outcrops. This island-like (allopatric) distribution pattern suggests very limited gene flow between populations. Furthermore, it would be impractical to verify experimentally “potential interbreeding” for each population, as *Plectostoma* populations occur in hundreds of different limestone outcrops.

Previous species circumscriptions in this genus have been mainly based on a morphological species concept. In gastropods, this is common practice. However, shell shape or even some shell structures, such as rib intensity, can be genetically variable and/or phenotypically plastic under different environmental conditions (e.g. Berry 1962, Kemp and Bertness 1984). Nevertheless, when the intra- and inter-specific variations in shells are understood (see below “Morphological analysis”), a morphological species concept may be used as one of the guidelines in species delimitation.

Finally, the phylogenetic species concept (sensu Cracraft 1983–“A species is the smallest diagnosable cluster of individual organism within which there is a parental pattern of ancestry and descent”) also cannot be used in the case of *Plectostoma* species, again because of their populations’ allopatric distribution on isolated limestone outcrops. The deposition age of Peninsular Malaysian limestone ranges from the Ordovician to the late Triassic (*ca.* 480 mya–200 mya). Though the exact time at which the limestone outcrop was exposed is unknown, van Benthem Jutting (1960) believed this began to happen after the Cretaceous - *ca.* 140 mya. Given the fact that *Plectostoma* species have been found on relatively young, Miocene age, limestone (*ca.* 24 mya–5 mya) in Borneo, it is likely that *Plectostoma* species will have colonised limestone hills in Peninsular Malaysia soon after the limestone hills were exposed. Hence, populations of the same *Plectostoma* species on each isolated limestone hill could have been separated for a long time and thus these isolated populations would appear as a several deeply diverged lineages in the phylogenetic tree (Liew TS, unpublished data). In view of this, the blind application of a phylogenetic species concept could inflate the number of species in *Plectostoma*.

Because of these problems, in this study, we have used elements of different species concepts for the delimitation of *Plectostoma* species. First we used a set of shell characters for initial morphological species delimitation. These groups were then checked for their distribution ranges. Whenever more than one of these morphological species was found at the same hill, we examined the genetic dissimilarity (in DNA barcode, COI) between these sympatric morphological species. In addition, the intra-specific genetic divergence was examined among several geographically separated populations of the same species (see below “COI Barcoding”). By reciprocal illumination from the morphological, genetic and distribution data, we determined for each species a set of characters that is stable within a species and diagnosable between *Plectostoma* species. We applied the same approach to the Bornean species (Liew et al. in prep).

### Morphological analysis

In conventional conchology, shell descriptions and measurements are mainly made based on the standard apertural view of the shell. In this view, the shell is positioned so that the columella is vertical and the shell is rotated around this columella axis until the aperture faces the user. After the apertural view of the shell is set, the shell linear measurements are taken and descriptions of other shell characters are made (see, e.g., Vermeulen 1994). However, the irregularity in the orientation of the aperture of *Plectostoma* shells, caused by the distortion in the shell coiling hinder this traditional conchological approach.

**Figures 1.**
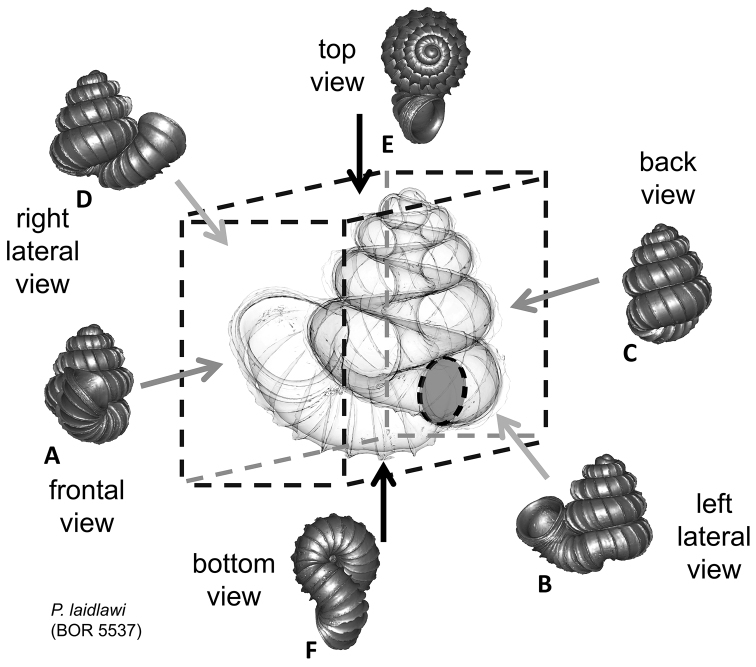
Six shell views. The shaded circle is the operculum. The frontal view (perpendicular on the operculum) was set as reference perspective for the other views: back, right lateral, left lateral, top, and bottom.

We feel that in *Plectostoma* the usefulness of this traditional approach is limited because of the presence of the tuba that deviate from the coiling axis of the spire. The varying length and coiling mode of the tuba prevent any standardisation of the ‘frontal’ view of the spire (Figure 1; see also Sasaki 2010, who refers to “different direction in shell-mouth opening”). Nevertheless, all previous authors use this method to describe and illustrate *Plectostoma* shells. Here, we proposed a better approach to describe the shells of this, conchologically unusual, genus.

In *Plectostoma*, the only part of the shell that can serve as a landmark to determine a frontal view, while at the same time fixing the position of both the spire as well as the tuba, is not the aperture, but the constriction, the point where the spire ends and the tuba begins, and operculum rests. Therefore, we determined the frontal view of the shell as follows: the shell is held with the coiling axis of the spire vertical and with the operculum perpendicular to the line of view, on the right side (because all *Plectostoma* shells are dextral). Left lateral view, back view and right lateral view are obtained by turning the shell 90°, 180°, and 270° from this starting point (Figure 1). Accordingly, all the shell characters were described based on this positioning scheme.

**Specimen images and 3D models.** To examine the shell morphology, at least one digital 3D model of each species was obtained. We used microcomputed X-ray tomography (CT) to obtain 3D models of *Plectostoma* shells. For each species, several empty shells and ethanol-preserved shells with the soft body inside, spaning the morphological variation breadth, were selected. Microcomputed tomography was carried in a high-resolution micro-CT scanner (SkyScan, model 1172, Aartselaar, Belgium). 3D models were created from the reconstructed images with the manufacturer’s software CT Analyser v1.12.0.0 (Skyscan©) and saved as digital polygon mesh objects (*.ply). The 3D models were then simplified by quadric edge collapse decimation (to ca. 200,000 faces) as implemented in the program MeshLab v1.3.2 (Cignoni et al. 2008).

The position and orientation of the 3D digital shell was manipulated so that the shell columella was in parallel with the z-axis and the operculum outer side was visible from a user perspective (Figure 1, Appendix 1(9)). Then, the outer operculum view of the shell was regarded as frontal view. After that, the field of view of the 3D model was set to orthographic, and an image was taken for each of the six perspectives: frontal view (A), left lateral view (B), back view (C), right lateral view (D), top view (E), and bottom view (F). In addition, two images were made of the constriction teeth of the parietal (G) and basal (H) inner shell whorl after clipping of the 3D model. All manipulation and imaging was done with MeshLab v1.3.2 (Cignoni et al. 2008). Thus, a total of eight images were made for each species (A–H). For video tutorials, see Appendix 1(10).

**Shell characters and descriptions.** The shell is an accretionary exoskeleton of the snail. The overall shape of the shell, which resembles a 3D spiral, results from changes in the curvature and torsion during the shell accretionary process, and form changes in aperture form during shell growth (Okamoto 1988). However, the exact quantifications of these changes might exceed the requirements of the practical purpose of this taxonomic paper. Therefore, we used traditional linear measurements to quantify the shell form, in a way that these measurements abstract the shell ontogeny and its 3D spiral properties.

**Figures 2.**
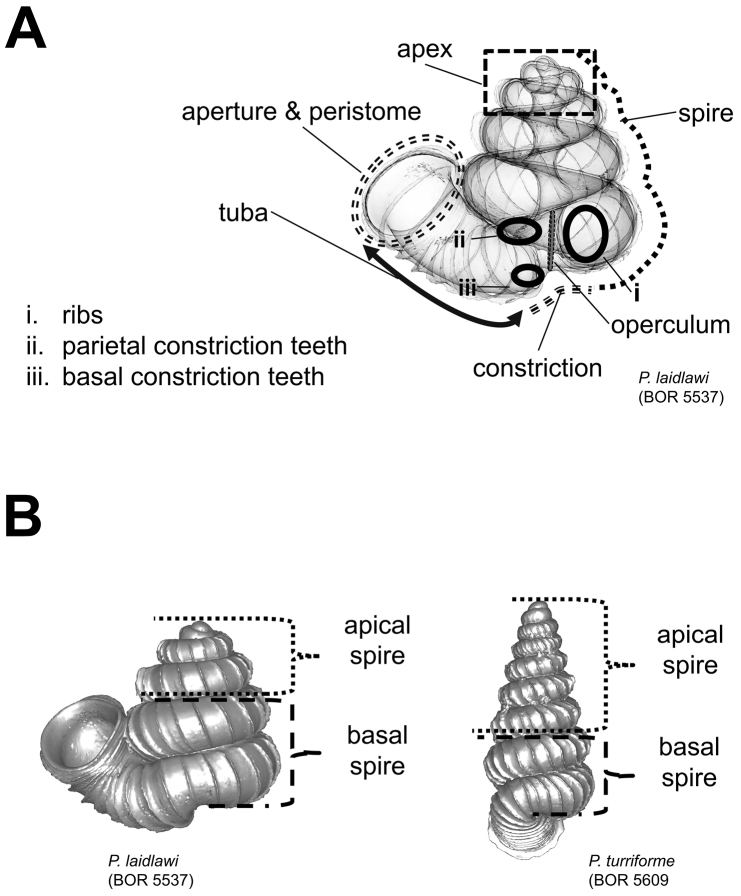
Morphological terminology and shell measurements. **A** shell part terminology as used in the species descriptions is shown in an example shell **B** Two shell examples show the basal spire that consist of the last two whorls of the spire, and the apical spire that consist of the remaining spire whorls.

**Figures 3.**
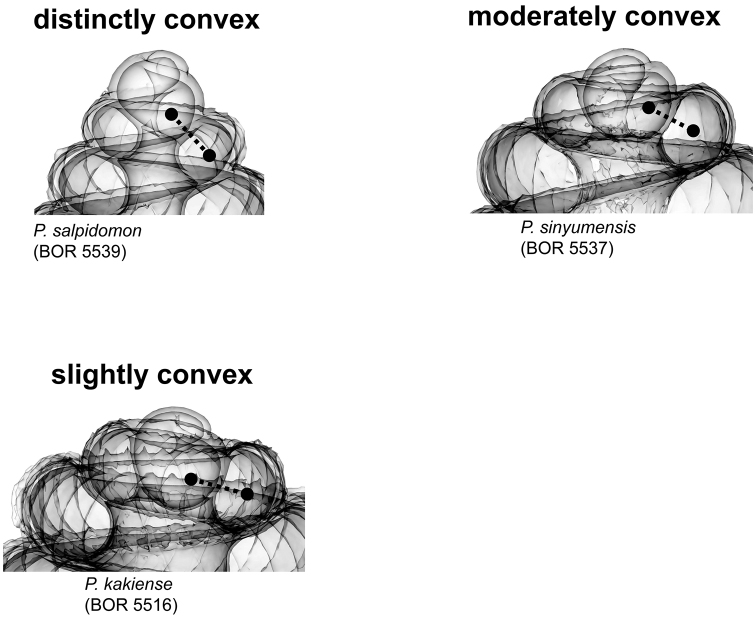
Apex forms in shell left lateral view. The degree of shell apex depression results from the growth regime of the teleoconch after the protoconch.

**Figures 4.**
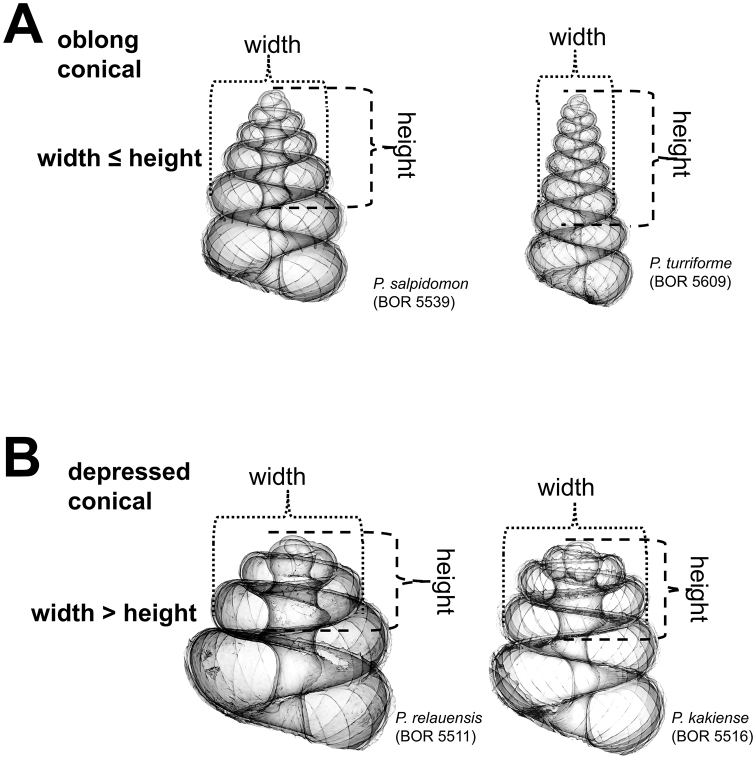
Two basic forms of apical spire whorls: **A** oblong conical **B** depressed conical.

**Figures 5.**
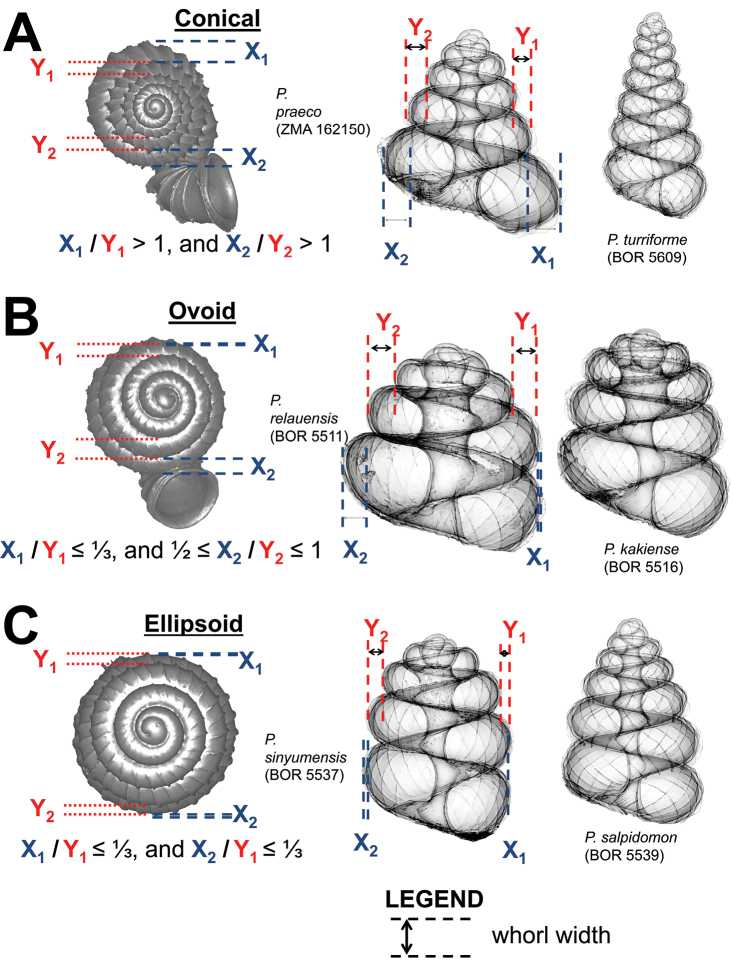
Three basic forms of shell spire basal whorls in left lateral view. Panels from left to right of each of the shell forms: quantifications of the shell form in top view; example of the shell form with a low number of whorls in left lateral view; example of the shell form with a higher number of whorls in left lateral view. **A** conical **B** ovoid **C** ellipsoid.

**Figures 6.**
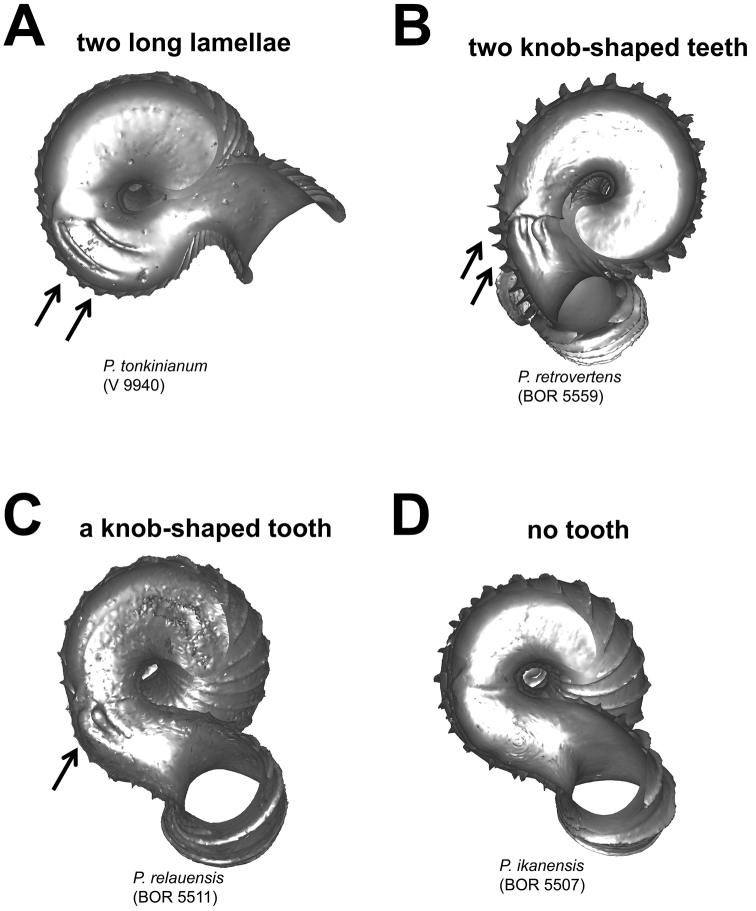
Different types of parietal constriction teeth before the operculum resting site, in bottom view. **A** two long lamellae run parallel to the whorl growing direction **B** two short ridges run parallel to the whorl growing direction, each knob-shaped at one end **C** a single ridge runs parallel to the whorl growing direction, knob-shaped at one end **D** no tooth.

**Figures 7.**
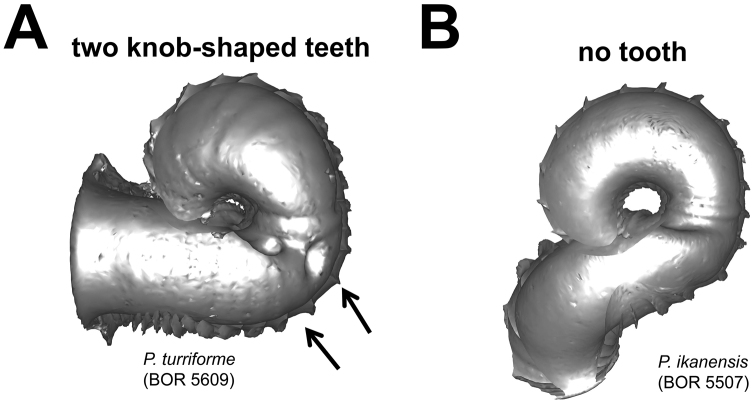
Different types of basal constriction teeth in top view. **A** two teeth after operculum resting site: one ridge runs parallel to the whorl growing direction; the other ridge has a knob at one end and runs perpendicular to the whorl growing direction **B** no tooth.

**Figures 8.**
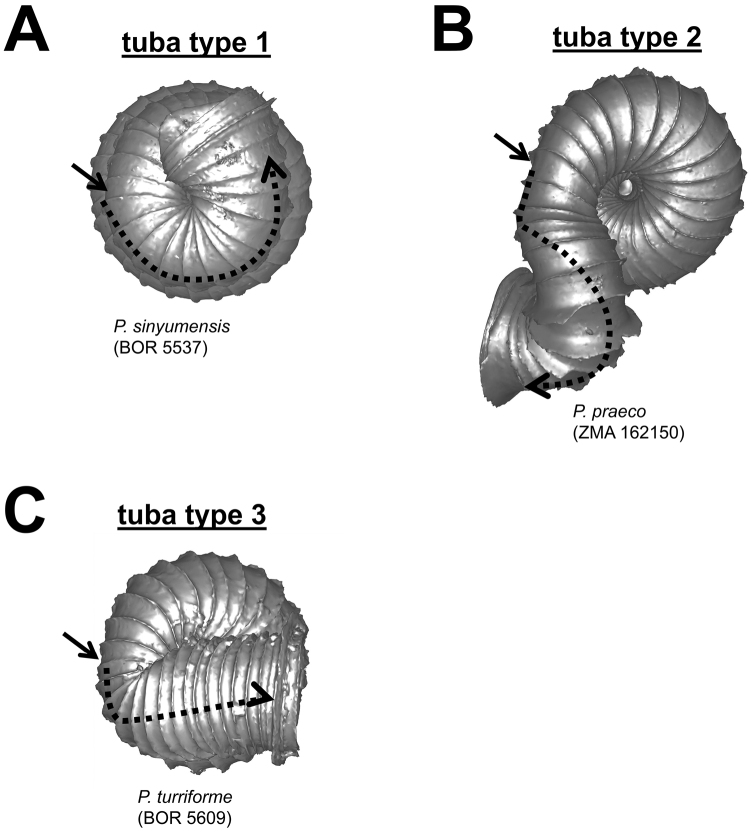
Different tuba coiling regimes in bottom view. **A** tuba type 1–tuba coiling as regularly as the spire last whorl **B** tuba type 2–tuba gradually coiling downward and then in a different direction **C** tuba type 3–tuba bent abruptly. Arrows point to the constriction.

**Figures 9.**
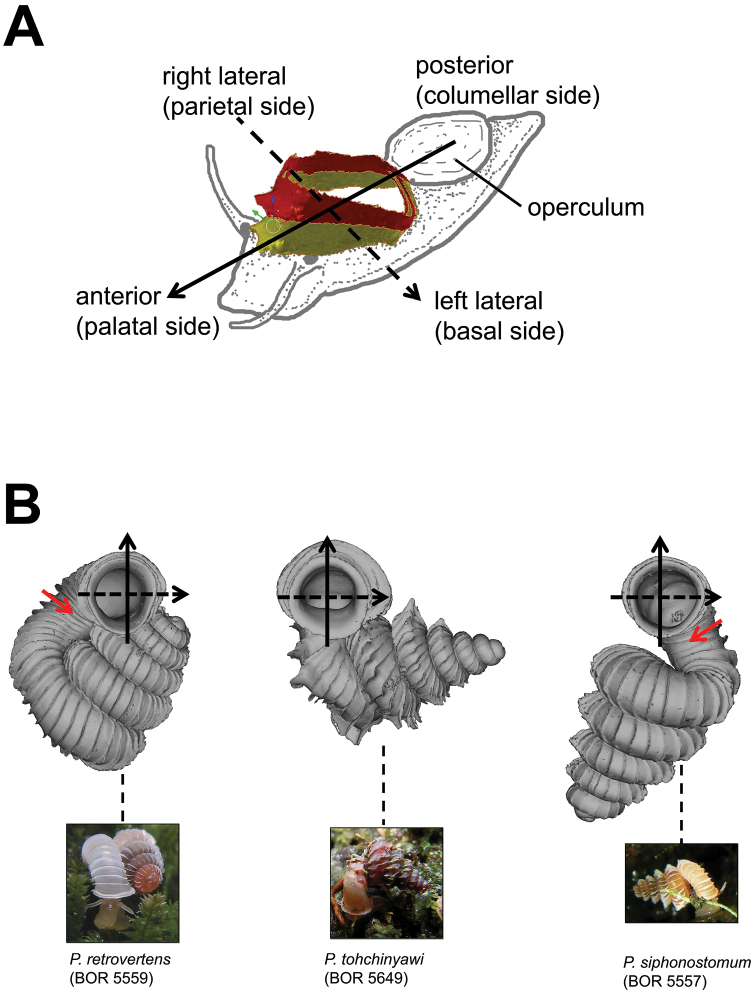
Positioning scheme for the description of aperture and peristomes. **A** anterior, posterior, right and left lateral sides of aperture and peristome are defined according to the orientation of the shell relative to the active animal **B** Three shell examples showing the defined positions of aperture and peristome. Red arrows point to the aperture area with the densest ribs.

**Figures 10.**
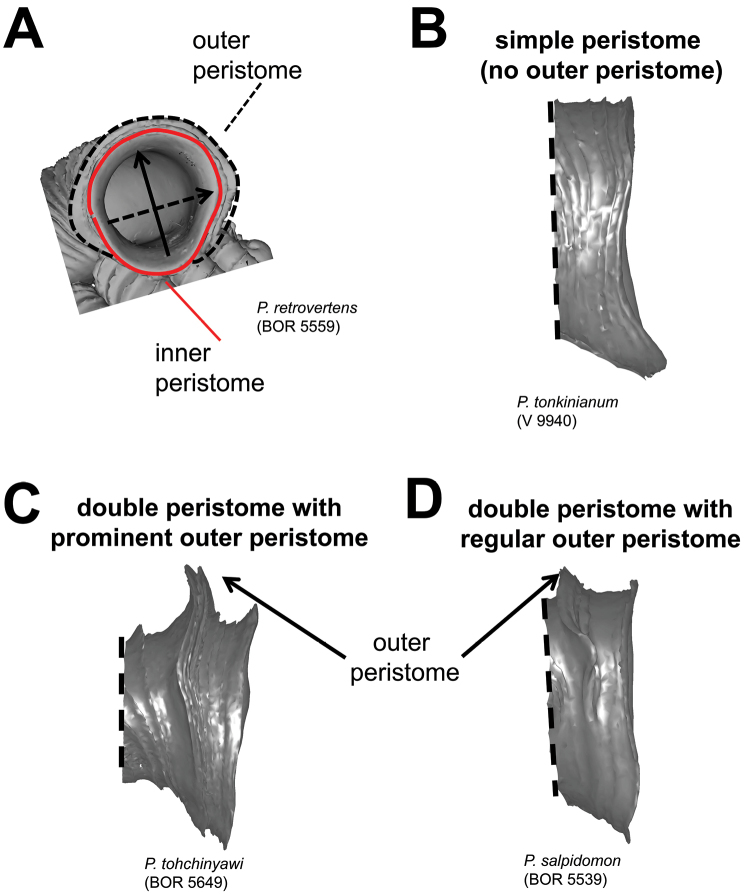
Aperture and peristome forms. **A** outer and inner peristome **B** Simple aperture without outer peristome **C** aperture with double peristomes in which the outer peristome is prominent **D** aperture with double peristomes in which the outer peristome is equally prominent as the inner.

**Figures 11.**
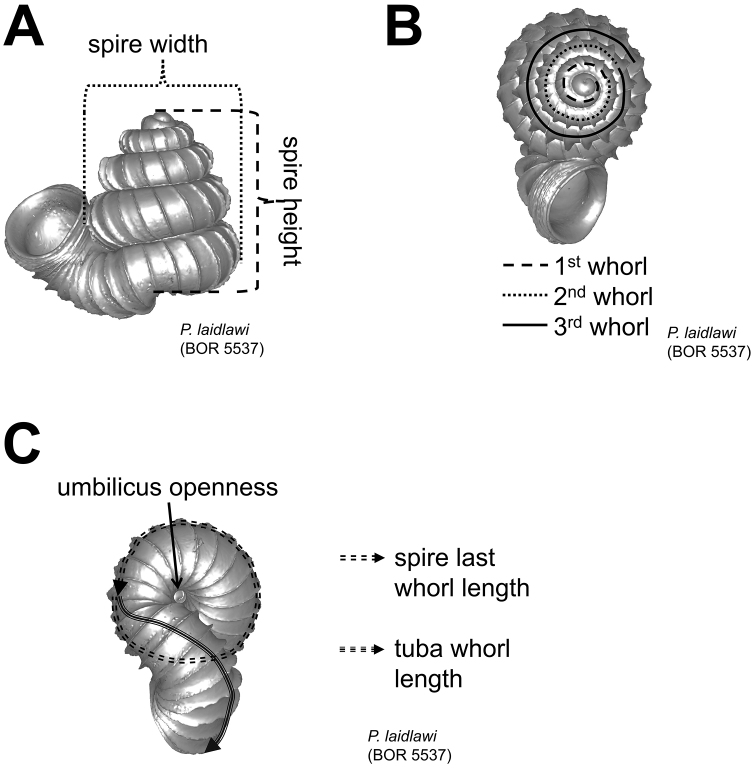
Shell measurements. **A** height and width of spire **B** number of whorls **C** spire last whorl length, tuba whorl length and umbilicus openness.

**Figures 12.**
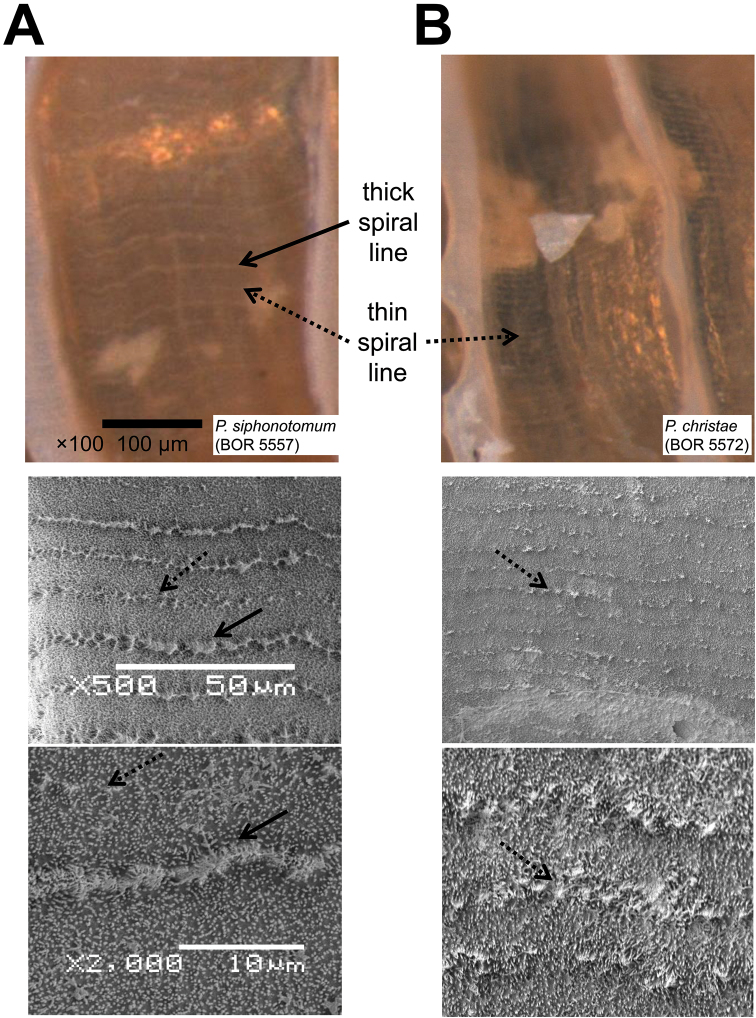
Spiral lines on the shell surface are shown in, from top to bottom, 100 × magnification under the dissecting microscope, 500 × and 2000 × magnification under scanning electron microscope. **A** shell with both thick and thin spiral lines **B** shell with only thin spiral lines. Each corresponding image in **A** and **B** is at the same scale.

**Figures 13.**
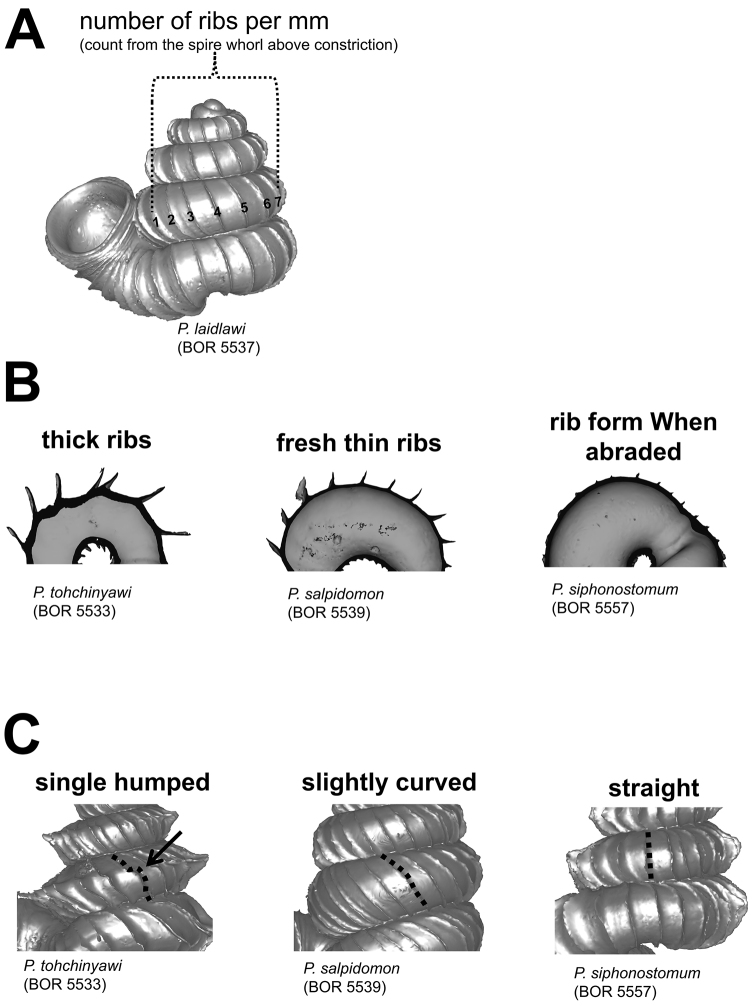
Radial ribs. **A** measurement of number of ribs per mm at the whorl above the constriction in left lateral view **B** rib intensity, from left to right: thick ribs, thin ribs, and abraded ribs **C** three different rib shapes, from left to right: single-humped, slightly curved and straight.

After the six views of a shell were determined as described (Figure 1), the shell whorls were described for each of six major parts, according to the shell ontogeny order: (1) apex–protoconch and the first teleoconch (Figure 2A, and 3); (2) apical spire–the whole teleoconch except the last 1 1/2 whorls before the constriction (Figure 2B, and 4); (3) basal spire–the last 1 1/2 whorls before the constriction (Figure 2B, and 5); (4) constriction–the narrowest transitional part of the whorl between spire and tuba (Figure 2A, 6, and 7); (5) tuba whorl–teleoconch after the constriction (Figure 2A, and 8); and (6) aperture and peristome (Figure 2A, 9, and 10). The first three parts constitute the shell spire, for which size and shape were quantified from the left lateral view. The height, width and number of whorls of the shell were measured and counted from the spire (Figure 11A, and B). In addition to the description of the general shell form, we recorded the shell surface ornamentations, namely, (7) fine spiral striation (Figure 12), and (8) distinct radial ribs (Figures 2A and 13).

**(1) Apex** – Ranges from “distinctly convex”, via “moderately convex”, to “slightly convex” (Figure 3). The slightly convex apex has a teleoconch that grows with less torsion and greater curvature than the protoconch, and vice versa in the distinctly convex apex.

**(2) Apical spire** – Similar to the apex form, less torsion and greater curvature in shell growth produce a “depressed conical” apical spire, and the reverse produces an “oblong conical” apical spire. The oblong and depressed conical shape of the spire can be estimated by measuring the ratio between the apical spire height and width (Figure 4).

**(3) Basal spire** – The curvature of the basal spire determines the final form of the spire. The width of the spire base is related to how tightly the basal spire whorl coils towards the shell columella, and to the whorl width. The basal spire shape categories, namely, conical, ovoid, and ellipsoid, can be estimated by comparing the difference between two whorl width measurements from three consecutive whorl peripheries (Figure 5). This measurement is made at both the left and the right side of the shell, in left lateral view. The spire umbilicus may be open, partially closed or totally closed by the tuba.

**(4) Constriction** – The constriction is a short transitional and narrow part of the whorl between the spire and tuba (Figure 2A). This is the furthest point to where the snail can retract into the shell and where the operculum rests (Figure 1, and 2A). Inside the constriction of some species, there are calcareous structures that protrude from the inner shell wall–constriction teeth (Figure 6 and 7). The number, shape, and location of the constriction teeth are taxonomically informative characters.

**(5) Tuba** – It is difficult to describe the variable forms of tuba in words. For the sake of convenience, we categorised the tuba into three coiling regimes, which represent how many times torsion changes drastically. These are: type 1–no drastic changes in torsion at the beginning of the tuba as compared to the spire; type 2–drastic changes in torsion at the beginning and midway of the tuba; and type 3–drastic changes in torsion at the beginning of tuba (Figure 8). In addition to the coiling regime, the overall shell form is also determined by the final tuba length. However, estimation of tuba length is difficult. Hence, we quantified the tuba length by estimating the ratio of the tuba periphery length on the one hand, and the spire last whorl periphery length on the other. In addition, we estimated the proportion of the tuba that attaches to the spire. Finally, the difference between tuba forms can also be determined by comparing the shell perspective where the aperture opening is best visible.

**(6) Aperture and peristome** – Before we characterise the aperture form, we define the anatomical position of the aperture according to the orientation of the animal inside (Figure 9). Our definitions for the four areas of aperture side correspond to the conventional terminology used for the aperture of a regularly coiling shell: Anterior = palatal side, posterior = columellar side, right lateral = parietal side and left lateral = basal side. The convenient way to recognise these aperture areas is by identifying the posterior area where it has the densest ribs (Figure 9B). After that we describe and compare the shape of the aperture and the outer peristome (Figure 10). The shell has either a simple or a double peristome, and this character is species specific in *Plectostoma*. The prominence and shape of the outer peristome is described by how much the outer peristome is projected at the anterior, posterior, and both lateral sides, as compared to the inner peristome (Figure 10A).

**(7) Spiral lines** – Spiral line sculpture on the shell is composed of a row of granulated micro-structures. The intensity of the spiral lines depends on the size of these micro-structures. In general, Thick spiral lines should be visible under the dissecting microscope at 50× magnification (Figure 12A), whereas thin spiral lines are hardly visible at 50× magnification, but are visible at 100× magnification (Figures 12A and B, Appendix 3). Thick spiral lines are more widely spaced (< 7 lines per 100 μm) than thin lines (> 10 lines per 100 μm). Thin lines may be present in between sparse thick lines (Figure 12A), but on living snails these may fade away when the snail ages and may be hardly visible in old empty shells.

**(8) Radial ribs** – Radial ribs are produced by a change of shell ontogeny in both shell accretion direction and aperture dimensions. During rib formation, the shell material accretion direction around the aperture changes from longitudinal to orthogonal; meanwhile, aperture size increases and probably aperture shape changes. Thus, the formation of each rib represents a discontinuity in shell growth in the longitudinal direction and a change of the aperture shape and size. In view of these, three characters can be observed in the radial ribs.

The first character is the total number of ribs and the spacing between them on the shell. The number of ribs for a species can be highly variable between different individuals. The ribs are not evenly distributed on the shell surface; for example, the spacing between ribs consistently increases from the apex to the last whorl of the spire (Appendix 4). In view of this, we describe the rib density as the number of ribs within 1 mm on the whorl above the shell constriction in left lateral view (Figure 13A).

The second character is the intensity of the ribs in terms of length and thickness. Generally, rib length is related to the spacing between the ribs; for example, the greater the spacing (whorl length) before the rib, the longer the rib projects from the whorl periphery (Liew T.S. unpublished data). Thus, the lengths of the ribs, from the apex to the last whorl of the shell, change in a trend similar to the rib spacing. Besides, the thickness of ribs can vary between species, but less so within species. The thickness of the ribs depends on the number of shell layers and the thicker the ribs, the more likely it is that ribs persist in old shells (Figure 13B).

The third character is the form of the ribs. As mentioned above, each radial rib on a *Plectostoma* shell was actually a deformed aperture during shell ontogeny. Thus, a more biologically meaningful way to describe the radial ribs is to compare the rib edge form (deformed aperture) to the whorl before the rib (regular aperture). The rib form can be either the same or different in the spire and tuba parts of the shell. As is the case with the aperture and peristome, the rib edge is either slightly or distinctly projected at its anterior side (i.e. at whorl periphery) as compared to the lateral sides (hereafter “rib plate”). In addition, the shape of the rib plate can be straight, slightly curved, or single-humped (Figure 13C). Although thinner ribs are easily abraded in old shells, at least the rib plate form can be inferred from the abraded scar at the whorl periphery (Figure 13B).

Finally, we noticed variation among species in the rib inclination, which can be estimated with respect to the coiling axis. However, it is difficult to quantify this inclination accurately. Nevertheless, we add this character into the description of the shell in a qualitative manner in the terms of orthoclin (i.e. ribs are almost straight with respect to the collumella, as in *Plectostoma christae* and *Plectostoma siphonostomum*), prosoclin (i.e. ribs are distinctly tilted with respect to the collumella, as in *Plectostoma tohchinyawi* and *Plectostoma salpidomon*).

In brief, shells of all species were described following a template consisting of three major elements (Table 1). First, the shell was described in several parts which represent the chronology of the shell ontogeny. Second, we defined a set of characters that can be determined in each shell part. Lastly, we described each character in either a quantitative or quantitative manner. We regard this template as a morphological model for the taxonomy of the genus *Plectostoma*, and each element in this template may be updated by future taxonomists when necessary. We present the final description of each species in a uniform telegraphic format (e.g. semantic-tagged by bold text, and colon “:”) so that these morphological data can be mined effectively. By doing this, we hope to reduce the redundant process where species descriptions are done de novo each time a taxonomist revises the same taxa (Deans et al. 2012).

**Table 1.** Format for species shell description.

Shell parts (Figure 2)	Characters for shell part†	Descriptions (see section “Morphological analysis” for details).
Apex.	Shape: #.	Figure 3.
Spire.	Height: #. Width: #. Number of whorls: #. Apical spire shape: #. Basal spire shape: #. Whorl periphery: #. Umbilicus: #.	Figure 2. Figure 4. Figure 5. Figure 11.
Constriction.	Parietal teeth: #. Basal teeth: #.	Figure 6. Figure 7.
Tuba.	Coiling direction: # and aperture visible in # view. Tuba whorl length in proportion to spire last whorl: #. Proportion of tuba that attaches to spire: #.	Figure 1. Figure 8. Figure 11.
Aperture and peristome.	Peristome: #. Outer peristome shape: #.	Figure 9. Figure 10.
Spiral lines.	Thick lines: #. Thin lines: #.	Figure 12.
Radial ribs.	Rib density: # ribs per mm. Rib intensity: #. Shape: #. Inclination: #.	Figure 13.

† “#” is the description for each shell character for Figure 1–13.

**Digital model.** Pictures are more effective than verbal descriptions for shell morphology. However, it is not feasible to have hundreds of pictures taken for each perspective of a shell. Many non-linear characteristics of a shell cannot effectively be representedby 2D images. Thus, an interactive 3D model shell improves the dissemination of morphological information. Presenting 3D models in digital publication has started five year ago (Ruthensteiner and Heß 2008), and since then more taxonomists have taken the initiative to embed 3D models in e-papers. However, in this paper we have refrained from embed 3D models in the paper itself, since this limits further analysis by readers. Instead, we provide sets of 3D data in *.blend files, which consists of all 3D models and which can be opened in Blender v2.63 (www.blender.org). The 3D models in the blend file can be exported to *.ply format, which can be opened in MeshLab v1.3.2 (Cignoni et al. 2008). Both Blender and MeshLab are freeware and can be used to analyse the 3D model further (e.g. measurements, modification, etc). For video tutorials, see Appendix 1(11 and 12).

### Conservation status assessment

We propose the conservation status for each species by following IUCN Red List Criteria and guidelines (IUCN Standards and Petitions Subcommittee 2013). We assess the conservation status based on our fieldwork in Malaysia between the year 2010 and year 2013 and the information obtained from the museum collections.

### Molecular phylogeny and COI barcoding

**Taxon sampling.** A total of 27 ingroup taxa of the genus *Opisthostoma* (n=11) and *Plectostoma* (n=16) were included in this study: six *Opisthostoma* species from Borneo, five *Opisthostoma* from Peninsular Malaysia, nine *Plectostoma* species from Borneo, and seven *Plectostoma* from Peninsular Malaysia. All of these ingroup taxa were selected on the basis of their distribution and shell forms which are representative for about 150 species in both genera. In addition to the ingroup taxa, eight outgroup taxa were included in the phylogenetic analysis. Sequence data for these outgroup taxa, which include three genera of the Diplommatinidae and a species of the Cochlostomatidae, were obtained from Webster et al. (2012). The details of these specimens and the Genbank accession numbers are listed in Table 2.

**Table 2.** Details of the taxa used for phylogenetic analysis and DNA barcoding.

no.	Species	Voucher codes	Locality (Country, state, location, Lat, Long)	Genbank accession no.			
				COI	16S	18S	28S
1	*Cochlostoma septemspirale* (Wagner, 1897)	RMNH.MOL.119825	Switzerland.	HM753326	HM753497	HM753423	HM753367
2	*Arinia paricostata* (Vermeulen, 1996)	RMNH.MOL.119779	Malaysia, Sabah.	n.a.	HM753500	HM753441	HM753384
3	*Diplommatina canaliculata* (Moellendorff, 1886)	RMNH.MOL.119783	Malaysia, Pahang.	HM753338	HM753504	HM753445	HM753388
4	*Diplommatina gomantongensis* (Smith, 1894)	RMNH.MOL.119800	Malaysia, Sabah.	HM753342	HM753509	HM753451	HM753394
5	*Diplommatina rubicunda* (Von Martens, 1864)	RMNH.MOL.119819	Malaysia, Sabah.	HM753363	HM753520	HM753477	HM753417
6	*Diplommatina rubra* (Godwin Austen, 1889)	RMNH.MOL.119797	Malaysia, Sabah.	HM753346	HM753514	HM753456	HM753399
7	*Palaina albata* (Beddome, 1889)	RMNH.MOL.119810	Belau, Peleliu.	HM753354	HM753531	HM753469	HM753409
8	*Palaina striolata* (Crosse, 1866)	RMNH.MOL.119812	Belau, Ngerekebesang.	HM753356	HM753533	HM753470	HM753410
9	*Opisthostoma hailei* Solem, 1964	n.a.	Malaysia, Sabah, 5.536, 118.213.	KC250856	KC250897	KC250923	KC250948
10	*Opisthostoma holzmarkii* Thompson, 1978	BOR 5580	Malaysia, Sarawak, 3.804, 113.78.	KC250871	KC250912	KC250937	KC250962
11	*Opisthostoma* sp.	BOR 5561	Malaysia, Sarawak, 4.044, 114.815.	KC250876	KC250917	KC250942	KC250967
12	*Opisthostoma brachyacrum brachyacrum* (Thompson, 1978)	BOR 5548	Malaysia, Sarawak, 1.319, 110.291.	KC250874	KC250915	KC250940	KC250965
13	*Opisthostoma brachyacrum brachyacrum* (Thompson, 1978)	BOR 5593	Malaysia, Sarawak, 3.804, 113.78.	KC250873	KC250914	KC250939	KC250964
14	*Opisthostoma* sp.	BOR 5561	Malaysia, Sarawak, 4.044, 114.815.	KC250878	KC250920	KC250945	KC250970
15	*Opisthostoma vermiculum* Clements & Vermeulen, 2008	BOR 5526	Malaysia, Perak, 4.55, 101.131.	n.a.	KC250910	n.a.	n.a.
16	*Opisthostoma paulucciae* Crosse & Nevill, 1879	BOR 5558	Malaysia, Pahang, 4.487, 101.976.	KC250866	KC250907	n.a.	n.a.
17	*Opisthostoma tenuicostatum* van Benthem Jutting, 1952	BOR 5525	Malaysia, Kelantan, 5.09, 102.22.	KC250869	n.a.	KC250935	KC250960
18	*Opisthostoma platycephalum* van Benthem Jutting, 1952	BOR 5576	Malaysia, Pahang, 3.908, 103.147.	KC250864	KC250905	KC250931	KC250956
19	*Opisthostoma tenerum* van Benthem Jutting, 1952	BOR 5577	Malaysia, Pahang, 3.984, 103.144.	KC250861	KC250902	KC250928	KC250953
20	*Plectostoma mirabile* (Smith, 1893)	n.a.	Malaysia, Sabah, 5.531, 118.071.	KC250867	KC250908	KC250933	KC250958
21	*Plectostoma obliquedentatum* (Vermeulen, 1994)	BOR 5607	Malaysia, Sabah, 4.726, 116.615.	KC250880	KC250922	KC250947	KC250972
22	*Plectostoma hosei* (Godwin-Austen, 1890)	BOR 5592	Malaysia, Sarawak, 3.804, 113.78.	KC250860	KC250901	KC250927	KC250952
23	*Plectostoma austeni* (Smith, 1894)	BOR 5589	Malaysia, Sarawak, 1.311, 110.293.	KC250863	KC250904	KC250930	KC250955
24	*Plectostoma wallacei busauense* (Smith, 1893)	BOR 5545	Malaysia, Sarawak, 1.323, 110.3.	KC250875	KC250916	KC250941	KC250966
25	*Plectostoma stellasubis* (Vermeulen, 1994)	BOR 5588	Malaysia, Sarawak, 3.804, 113.78.	KC250858	KC250899	KC250925	KC250950
26	*Plectostoma pulchellum* (Godwin-Austen, 1890)	BOR 5563	Malaysia, Sarawak, 4.044, 114.815.	KC250857	KC250898	KC250924	KC250949
27	*Plectostoma cookei* (Smith, 1894)	BOR 5591	Malaysia, Sarawak, 3.804, 113.78.	n.a.	KC250918	KC250943	KC250968
28	*Plectostoma grandispinosum* (Godwin-Austen, 1889)	BOR 5590	Malaysia, Sarawak, 3.804, 113.78.	KC250879	KC250921	KC250946	KC250971
29	*Plectostoma christae* (Maassen, 2001)	BOR 5509	Malaysia, Kelantan, Limestone hill on the right hand side of the road no. 8 direction to Gua Musang, opposite to Quarry Damai, 4.896, 102.137.	KC420288	n.a.	n.a.	n.a.
30	*Plectostoma christae* (Maassen, 2001)	BOR 5505	Malaysia, Kelantan, Bukit Sejuk near Quarry Damai, along the road no. 8 from Gua Musang to Kuala Krai, 4.905, 102.121.	KC420287	n.a.	n.a.	n.a.
31	*Plectostoma christae* (Maassen, 2001)	BOR 5505	Malaysia, Kelantan, Bukit Sejuk near Quarry Damai, along the road no. 8 from Gua Musang to Kuala Krai, 4.905, 102.121.	KC420294	n.a.	n.a.	n.a.
32	*Plectostoma christae* (Maassen, 2001)	BOR 5506	Malaysia, Kelantan, Limestone hills `Ciku 2`. In the FELDA plantation Ciku 2, 4.924, 102.177.	KC420305	n.a.	n.a.	n.a.
33	*Plectostoma christae* (Maassen, 2001)	BOR 5506	Malaysia, Kelantan, Limestone hills `Ciku 2`. In the FELDA plantation Ciku 2, 4.924, 102.177.	KC420303	n.a.	n.a.	n.a.
34	*Plectostoma christae* (Maassen, 2001)	BOR 5572	Malaysia, Kelantan, Limestone in FELDA Ciku 5, 5.004, 102.2.	KC420271	n.a.	n.a.	n.a.
35	*Plectostoma christae* (Maassen, 2001)	BOR 5509	Malaysia, Kelantan, Limestone hill on the right hand side of the road no. 8 direction to Gua Musang, opposite to Quarry Damai, 4.896, 102.137.	KC420298	n.a.	n.a.	n.a.
36	*Plectostoma christae* (Maassen, 2001)	BOR 5505	Malaysia, Kelantan, Bukit Sejuk near Quarry Damai, along the road no. 8 from Gua Musang to Kuala Krai, 4.905, 102.121.	KC420269	n.a.	n.a.	n.a.
37	*Plectostoma crassipupa* (van Benthem Jutting, 1952)	BOR 5512	Malaysia, Kelantan, Limestone hill near Kampung Paloh, on the right hand side of the road no 8 to Gua Musang, 4.992, 102.228.	KC420304	n.a.	n.a.	n.a.
38	*Plectostoma crassipupa* (van Benthem Jutting, 1952)	BOR 5512	Malaysia, Kelantan, Limestone hill near Kampung Paloh, on the right hand side of the road no 8 to Gua Musang, 4.992, 102.228.	KC420273	n.a.	n.a.	n.a.
39	*Plectostoma crassipupa* (van Benthem Jutting, 1952)	BOR 5515	Malaysia, Kelantan, Limestone hill in rubber estate (Ulu Kumbang FELCRA), 4.69, 101.989.	KC420295	n.a.	n.a.	n.a.
40	*Plectostoma davisoni* sp. n.	BOR 5508	Malaysia, Kelantan, Limestone hill on the right hand side of the road D29, km 17 from Jelawang to Gua Musang, 4.985, 101.965.	KC250872	KC250913	KC250938	KC250963
41	*Plectostoma davisoni* sp. n.	BOR 5508	Malaysia, Kelantan, Limestone hill on the right hand side of the road D29, km 17 from Jelawang to Gua Musang, 4.985, 101.965.	KC420264	n.a.	n.a.	n.a.
42	*Plectostoma dindingensis* sp. n.	BOR 5612	Malaysia, Pahang, Dinding, 3.846, 102.378.	KC420285	n.a.	n.a.	n.a.
43	*Plectostoma ikanensis* sp. n.	BOR 5504	Malaysia, Kelantan, Gua Ikan. Loc 1, 5.353, 102.026.	KC420286	n.a.	n.a.	n.a.
44	*Plectostoma ikanensis* sp. n.	BOR 5507	Malaysia, Kelantan, Gua Ikan. Loc 1, 5.353, 102.026.	KC420302	n.a.	n.a.	n.a.
45	*Plectostoma ikanensis* sp. n.	BOR 5504	Malaysia, Kelantan, Gua Ikan. Loc 1, 5.353, 102.026.	KC420301	n.a.	n.a.	n.a.
46	*Plectostoma ikanensis* sp. n.	BOR 5504	Malaysia, Kelantan, Gua Ikan. Loc 1, 5.353, 102.026.	KC250862	KC250903	KC250929	KC250954
47	*Plectostoma ikanensis* sp. n.	BOR 5507	Malaysia, Kelantan, Gua Ikan. Loc 1, 5.353, 102.026.	KC420278	n.a.	n.a.	n.a.
48	*Plectostoma ikanensis* sp. n.	BOR 5504	Malaysia, Kelantan, Gua Ikan. Loc 1, 5.353, 102.026.	KC420268	n.a.	n.a.	n.a.
49	*Plectostoma kakiense* (Tomlin, 1948)	BOR 5516	Malaysia, Perlis, Kaki Bukit, 6.645, 100.202.	KC420265	n.a.	n.a.	n.a.
50	*Plectostoma tohchinyawi* sp. n.	BOR 5533	Malaysia, Terengganu, Tasik Kenyir. Gua Bewah, 4.851, 102.723.	KC420289	n.a.	n.a.	n.a.
51	*Plectostoma tohchinyawi* sp. n.	BOR 5533	Malaysia, Terengganu, Tasik Kenyir. Gua Bewah, 4.851, 102.723.	KC420307	n.a.	n.a.	n.a.
52	*Plectostoma kubuensis* sp. n.	BOR 5519	Malaysia, Perlis, Bukit Kubu. Loc 3, 6.404, 100.144.	KC420272	n.a.	n.a.	n.a.
53	*Plectostoma kubuensis* sp. n.	BOR 5519	Malaysia, Perlis, Bukit Kubu. Loc 3, 6.404, 100.144.	KC420270	n.a.	n.a.	n.a.
54	*Plectostoma laidlawi* (Sykes, 1902)	BOR 5510	Malaysia, Kelantan, Limestone hill in Kampung Bayu. About 337 km from Kuala Lumpur by road no. 8, 5.09, 102.22.	KC420279	n.a.	n.a.	n.a.
55	*Plectostoma laidlawi* (Sykes, 1902)	BOR 5510	Malaysia, Kelantan, Limestone hill in Kampung Bayu. About 337 km from Kuala Lumpur by road no. 8, 5.09, 102.22.	KC420300	n.a.	n.a.	n.a.
56	*Plectostoma laidlawi* (Sykes, 1902)	BOR 5571	Malaysia, Kelantan, Limestone in FELDA Ciku 4, 5.043, 102.196.	KC420275	n.a.	n.a.	n.a.
57	*Plectostoma mengaburensis* sp. n.	BOR 5574	Malaysia, Pahang, Sri Jaya, Gunung Mengapur, 3.731, 102.821.	KC420299	n.a.	n.a.	n.a.
58	*Plectostoma mengaburensis* sp. n.	BOR 5574	Malaysia, Pahang, Sri Jaya, Gunung Mengapur, 3.731, 102.821.	KC420267	n.a.	n.a.	n.a.
59	*Plectostoma palinhelix* (van Benthem Jutting, 1952)	BOR 5520	Malaysia, Pahang, Bukit Serdam, 3.83, 101.927.	KC250877	KC250919	KC250944	KC250969
60	*Plectostoma palinhelix* (van Benthem Jutting, 1952)	BOR 5520	Malaysia, Pahang, Bukit Serdam, 3.83, 101.927.	KC420283	n.a.	n.a.	n.a.
61	*Plectostoma relauensis* sp. n.	BOR 5511	Malaysia, Kelantan, Taman Negara, Sungai Relau Station. Gua Gajah, 4.642, 102.063.	KC420277	n.a.	n.a.	n.a.
62	*Plectostoma retrovertens* (Tomlin, 1938)	BOR 5559	Malaysia, Pahang, Bukit Chintamanis, 3.446, 102.014.	KC420297	n.a.	n.a.	n.a.
63	*Plectostoma salpidomon* (van Benthem Jutting, 1952)	BOR 5539	Malaysia, Pahang, Kenong Rimba Park. Gunung Kesong, 4.194, 102.177.	KC420276	n.a.	n.a.	n.a.
64	*Plectostoma salpidomon* (van Benthem Jutting, 1952)	BOR 5542	Malaysia, Pahang, Kenong Rimba Park. Gunung Tangkup, 4.194, 102.177.	KC420296	n.a.	n.a.	n.a.
65	*Plectostoma salpidomon* (van Benthem Jutting, 1952)	BOR 5541	Malaysia, Pahang, Kenong Rimba Park. Gunung Telahup, 4.194, 102.177.	KC420266	n.a.	n.a.	n.a.
66	*Plectostoma salpidomon* (van Benthem Jutting, 1952)	BOR 5569	Malaysia, Pahang, Gua Bama, 4.194, 101.967.	KC420280	n.a.	n.a.	n.a.
67	*Plectostoma salpidomon* (van Benthem Jutting, 1952)	BOR 5569	Malaysia, Pahang, Gua Bama, 4.194, 101.967.	KC250868	KC250909	KC250934	KC250959
68	*Plectostoma senex* (van Benthem Jutting, 1952)	BOR 5575	Malaysia, Pahang, Gua Charas, 3.908, 103.147.	KC250859	KC250900	KC250926	KC250951
69	*Plectostoma sinyumensis* (Maassen, 2001)	BOR 5537	Malaysia, Pahang, Gunung Jebak Puyuh, near Gunung Senyum, 3.7, 102.453.	KC420291	n.a.	n.a.	n.a.
70	*Plectostoma sinyumensis* (Maassen, 2001)	BOR 5537	Malaysia, Pahang, Gunung Jebak Puyuh, near Gunung Senyum, 3.7, 102.453.	KC250870	KC250911	KC250936	KC250961
71	*Plectostoma siphonostomum* (van Benthem Jutting, 1952)	BOR 5513	Malaysia, Kelantan, Taman Negara, Sungai Relau Station. Gua Gajah, 4.642, 102.063.	KC420292	n.a.	n.a.	n.a.
72	*Plectostoma siphonostomum* (van Benthem Jutting, 1952)	BOR 5513	Malaysia, Kelantan, Taman Negara, Sungai Relau Station. Gua Gajah, 4.642, 102.063.	KC420274	n.a.	n.a.	n.a.
73	*Plectostoma siphonostomum* (van Benthem Jutting, 1952)	BOR 5557	Malaysia, Pahang, Limestone hill on the left hand site of the road no. 8 toward Kuala Lipis. Near Kampung Chegar Perah I and II FELDA, 4.487, 101.976.	KC420306	n.a.	n.a.	n.a.
74	*Plectostoma siphonostomum* (van Benthem Jutting, 1952)	BOR 5557	Malaysia, Pahang, Limestone hill on the left hand site of the road no. 8 toward Kuala Lipis. Near Kampung Chegar Perah I and II FELDA, 4.487, 101.976.	KC420293	n.a.	n.a.	n.a.
75	*Plectostoma siphonostomum* (van Benthem Jutting, 1952)	BOR 5557	Malaysia, Pahang, Limestone hill on the left hand site of the road no. 8 toward Kuala Lipis. Near Kampung Chegar Perah I and II FELDA, 4.487, 101.976.	KC250865	KC250906	KC250932	KC250957
76	*Plectostoma whitteni* sp. n.	BOR 5536	Malaysia, Terengganu, Tasik Kenyir. Gua Taat, 4.842, 102.721.	KC420284	n.a.	n.a.	n.a.
77	*Plectostoma whitteni* sp. n.	BOR 5536	Malaysia, Terengganu, Tasik Kenyir. Gua Taat, 4.842, 102.721.	KC420282	n.a.	n.a.	n.a.
78	*Plectostoma umbilicatum* (van Benthem Jutting, 1952)	BOR 5503	Malaysia, Pahang, Gua Tongkat, 3.891, 102.473.	KC420281	n.a.	n.a.	n.a.
79	*Plectostoma umbilicatum* (van Benthem Jutting, 1952)	BOR 5503	Malaysia, Pahang, Gua Tongkat, 3.891, 102.473.	KC420290	n.a.	n.a.	n.a.

**DNA extraction, PCR and sequencing.** DNA extraction was done for each specimen (entire animal and its shell), with the E.Z.N.A. Mollusc DNA kit (OMEGA bio-tek). We followed the manufacturer’s extraction protocol. After extraction, PCR was carried out to amplify four regions, namely, 16S (mitochondrial, Palumbi 1996), COI (mitochondrial, Folmer et al. 1994), 28S (nuclear, Park and Foighil 2000), and 18S (nuclear, Stothard et al. 2000). We used the PCR reactions and programs of Webster et al. (2000). Positive PCR products were sequenced by Macrogen sequencing service (Macrogen Inc., Europe).

**Phylogenetic inferences.** Alignment of sequences was done with Bioedit v7.1.3 (Hall 1999) and adjusted manually. The final aligned data matrix consists of 2241 positions, of which 2092 can be aligned unambiguously (Appendix 17). The remaining 149 characters (91 from 16S and 58 from 28S) were excluded from further analysis. Mr.Modeltest v2.3 (Nylander 2004) was used to select the most appropriate model, based on the Akaike Information Criterion (AIC) for 16S, 18S and 28S, and as well as each of the three codon positions for COI. The best fits were: the GTR+I+Γ model for 16S, 28S, COI(2nd codon) and COI (3rd codon); the GTR+Γ for COI(1st codon); and the SYM+I+Γ for 18S.

Three phylogenetic analyses were done, namely, Bayesian inference (BI), Maximum Likelihood analysis (ML), and Parsimony analyses (PA). Bayesian inference was run in MrBayes v3.2.1 (Huelsenbeck and Ronquist 2001) with the following setting: mcmc ngen=5000000; nchains=4; samplefreq=100; average deviation of split frequencies < 0.01; and a burn-in value of 25%. A Maximum Likelihood analysis was run using RaxML v7.2.6 (Stamatakis 2006) as implemented on CIPRES portal v2.2 (Miller et al. 2010) on all the genes together, with 1000 rapid bootstraps using GTR + U. The data was divided into six partitions, all analyzed with a GTR substitution model. The Parsimony analyses were run using PAUP v4.0b (Swofford 1998). Gaps were set as fifth character state. A bootstrapped heuristic search with 1000 bootstrap replicates, and 10 random addition sequence heuristic search replicates, with no rearrangement limit per replicate was carried out, with 50% as the minimum bootstrap support included.

**COI Barcoding.** As mentioned above, the shell morphology alone may not be sufficient to delimit many species that have greater morphological variation within species. Thus, in addition to shell morphology, we sought another way to make the decision about the species delimitation. DNA barcoding is used to provide more insight into the species delimitation for gastropods (Boeters and Knebelsberger 2012, Puillandre et al. 2012). Therefore, we sequenced COI (mitochondrial, Folmer et al. 1994) for standard DNA barcoding analysis for a greater number of individuals than we used for the phylogenetic analysis. In total, we obtained COI sequences from 51 individuals, which comprise 19 species (including 8 new species) (Table 2). Then, the pairwise genetic distances for all the 51 sequences were computed by using Kimura 2-parameters in MEGA5 (Tamura et al. 2011).

**Genetic data repository.** The collection information for the specimens used were uploaded, stored and managed in Barcoding of Life Database (BOLD, http://www.boldsystems.org, Ratnasingham and Hebert 2007). After that, all DNA sequences were uploaded to NCBI GenBank (http://www.ncbi.nlm.nih.gov/genbank, Benson et al. 1997) via BOLD. Genetic data deposited in GenBank can be retrieved easily with the Python tool Biopython (e.g. Module “Entrez”) (http://biopython.org, Cock et al. 2009).

## Data resources

The data underpinning the analysis reported in this paper are deposited in the Figshare Repository at http://dx.doi.org/10.6084/m9.figshare.830412.

## Results and discussion

### Taxonomic data depository and cybertaxonomy

**Figures 14.**
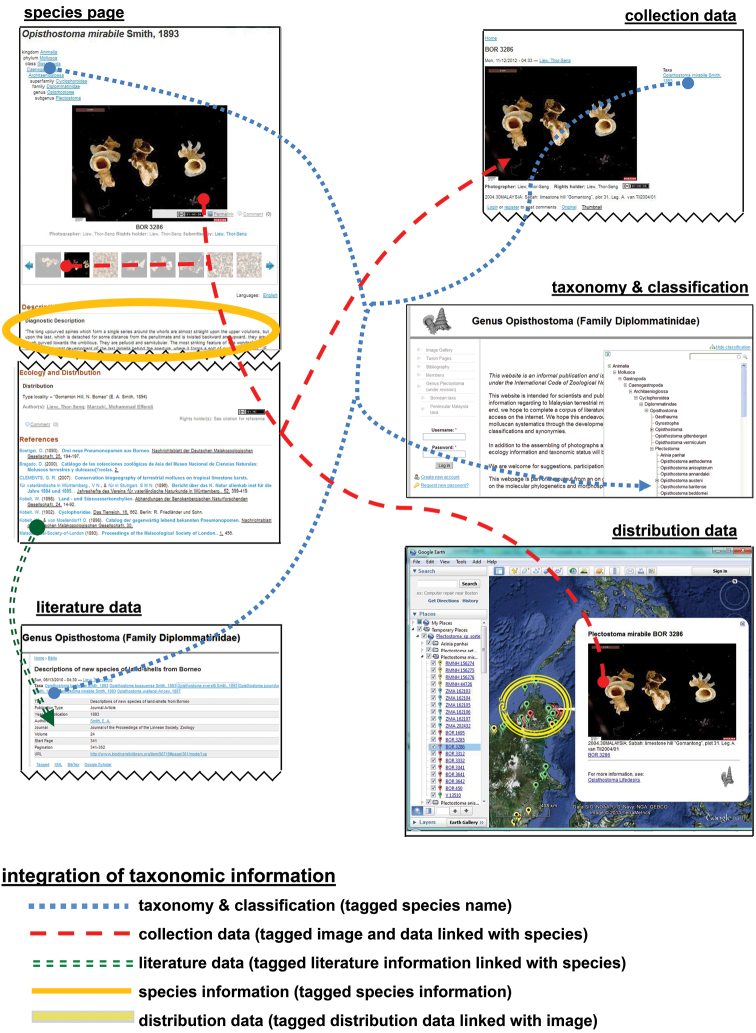
User interface of the Lifedesks online database and Google Earth. Lifedesks was used for data storage, management, and tagging; Google Earth was used for data sorting and exploration.

**Figures 15.**
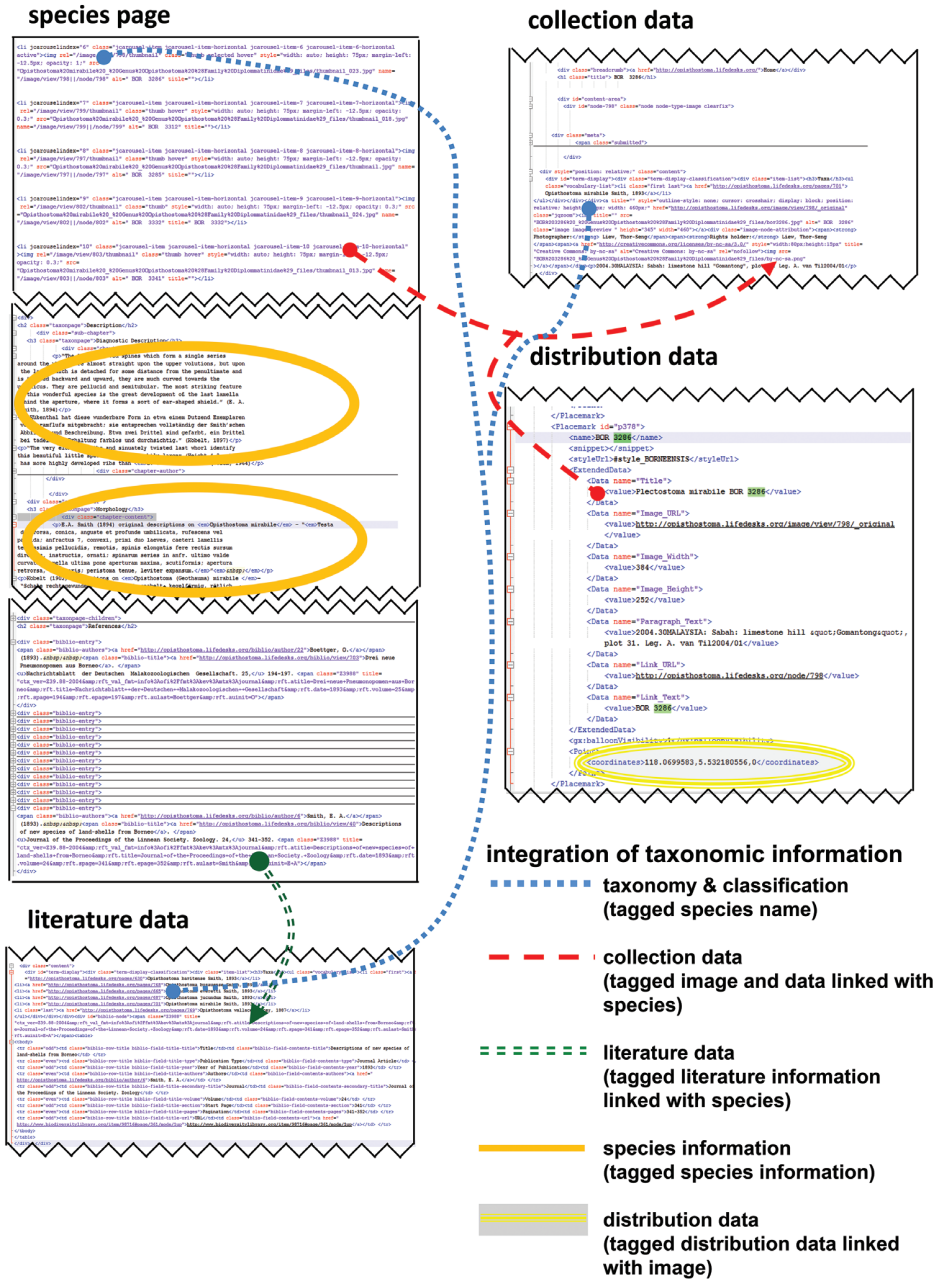
The raw tagged and linked data (in XML format) that underlay the Lifedesks online database and Google Earth can be accessed and used without the need of a specific platform.

While uploading the data of 31 species, 62 references, 214 collections, 290 pictures, and 31 species pages to Lifedesks, they were simultaneously tagged and linked to each other (Figure 14). The textual information can be downloaded and accessed offline in the xhtml format (source code) (Figures 15). Similarly, the taxonomic information that was used in creating the KML file can be retrieved in KML format (source code) (Figures 15, Appendix 5). In addition to the taxonomic data, a total of 155 3D models of 29 *Plectostoma* species were constructed, which belong to 86 samples. For the ease of specimen and species comparison, all 3D models were saved in ten separate. blend files, each containing 20 layers (Appendix 6–15). The 3D models (i.e. specimens) that belong to the same species were saved in the same layer.

Most taxonomists will have established their own workflow when working with taxonomic data. However, often only fractions of such data are published in a taxonomic revision paper. Thus, extra work would be needed to upload the data to online data platforms whenever a taxonomic revision is done in a traditional manner (i.e. not using cybertaxonomic tools). Here, we demonstrated that, in fact, no extra works is required if a cybertaxonomic workflow is adopted. Taxonomists themselves, who maintain, tag, link and create the data will benefit most by using these cybertaxonomy tools. For example, with some existing tools, such as Google Earth, the information can be integrated, explored and analysed in an interactive way, which will increase the efficiency of the taxonomic methodology (sensu Figure 1 in Sluys 2013; Appendix 5).

Furthermore, all textual information is preserved, and can be simply accessed in raw format. In other words, this information is not locked and can be retrieved and integrated with very basic programming skills (such as Python) even when the desirable platform (internet) and software are not available. More importantly, adopting this workflow in taxonomy practice will help realise the vision of Wheeler et al. (2012), that “The resultant cyber-enabled taxonomy, or cybertaxonomy, would open access to biodiversity data to developing nations, assure access to reliable data about species, and change how scientists and citizens alike access, use and think about biological diversity information”.

### Morphological analysis

Table 3 shows the morphological data matrix of 31 *Plectostoma* species and 11 general qualitative shell characters. Twenty-seven out of 31 *Plectostoma* species have a unique set of shell character states. The remaining four species share two unique sets of general characters states, namely, 1) *Plectostoma salpidomon* (van Benthem Jutting, 1952), and *Plectostoma laemodes* (van Benthem Jutting, 1961); and 2) *Plectostoma dindingensis* sp. n., and *Plectostoma mengaburensis* sp. n. However, each of these four species is distinguishable by other shell characters (see diagnosis and species description in the taxonomy section).

**Table 3.** Characters matrix for 31 *Plectostoma* species.

Species that share the same general character state	Species	Apex form	Apical spire form	Basal spire form	Parietal constriction teeth	Basal constriction teeth	Tuba form	Aperture view	Peristome types	Spiral lines	Rib shape	Rib thickness
‡	*Plectostoma salpidomon* (van Benthem Jutting, 1952)	1	2	2	2	2	2	2	2	2	2	2
‡	*Plectostoma laemodes* (van Benthem Jutting, 1961)	1	2	2	2	2	2	2	2	2	2	2
|	*Plectostoma dindingensis* sp. n.	2	2	2	4	2	1	3	2	1	3	2
|	*Plectostoma mengaburensis* sp. n.	2	2	2	4	2	1	3	2	1	3	2
§	*Plectostoma ikanensis* sp. n. Form BOR 5504	2	2	1	4	2	2	1	2	1	3	2
§	*Plectostoma ikanensis* sp. n. Form BOR 5507	2	2	1	4	2	2	1	2	1	3	2
	*Plectostoma annandalei* (Sykes, 1903)	1	2	1	?	?	2	2	2	?	3	?
	*Plectostoma kayiani* sp. n.	2 or 3	2	1	4	2	2	1	2	2	1	2
	*Plectostoma charasense* (Tomlin, 1948)	1	1	1	2	1	2	2	2	2	2	1
	*Plectostoma christae* (Maassen, 2001)	2	2	2	2	2	1	4	2	2	3	2
	*Plectostoma crassipupa* (van Benthem Jutting, 1952)	2 or 3	2	2	4	2	3	4	2	1	3	2
	*Plectostoma davisoni* sp. n.	2 or 3	2	2	2	2	2	1	2	1	3	2
	*Plectostoma kakiense* (Tomlin, 1948)	2 or 3	2	2	2	2	2	2	2	2	3	2
	*Plectostoma kitteli* (Maassen, 2002)	1	1	1	2	2	2	2	2	2	2	2
	*Plectostoma klongsangensis* (Panha, 1997)	1	1	1	?	?	2	2	2	1	1	1
	*Plectostoma kubuensis* sp. n.	2 or 3	2	2	4	2	2	2	2	2	3	2
	*Plectostoma laidlawi* (Sykes, 1902)	1 or 2	2	1	2	2	2	2	2	1	3	2
	*Plectostoma palinhelix* (van Benthem Jutting, 1952)	3	2	2	2	2	2	3	2	1	3	2
	*Plectostoma panhai* (Maassen, 2001)	2	2	2	3	2	1	3	2	1	3	2
	*Plectostoma praeco* (van Benthem Jutting, 1961)	1	2	1	2	2	2	2	2	2	2	2
	*Plectostoma relauensis* sp. n.	2 or 3	2	2	3	2	2	1	2	1	3	2
	*Plectostoma retrovertens* (Tomlin, 1938)	2	2	2	2	2	2	3	2	1	3	2
	*Plectostoma sciaphilum* (van Benthem Jutting, 1952)	1	1	2	2	2	3	4	2	1	2	2
	*Plectostoma senex* (van Benthem Jutting, 1952)	1	1	2	2	1	3	4	2	2	2	2
	*Plectostoma sinyumensis* (Maassen, 2001)	2	2	3	4	2	1	3	2	2	3	2
	*Plectostoma siphonostomum* (van Benthem Jutting, 1952)	1	1	1	2	2	1	4	2	1	3	2
	*Plectostoma tenggekensis* sp. n.	2	2	1	2	2	2	2	2	2	1	2
	*Plectostoma tohchinyawi* sp. n.	1	1	1	2	2	2	2	2	1	1	1
	*Plectostoma tonkinianum* (Dautzenberg & Fischer, 1905)	1	2	2	1	2	3	1	1	2	2	2
	*Plectostoma turriforme* (van Benthem Jutting, 1952)	1	1	2	1	1	3	4	2	2	2	2
	*Plectostoma umbilicatum* (van Benthem Jutting, 1952)	2	1	2	4	2	1	3	2	1	3	2
	*Plectostoma whitteni* sp. n.	1	2	2	4	2	3	1	2	2	3	2

**Characters Character states:** Apex form (Figure 4) 1 = distinctly convex; 2 = moderately convex; 3 = slightly convex. Apical spire form (Figure 5) 1 = oblong; 2 = depressed. Basal spire form (Figure 6) 1 = conical; 2 = ovoid; 3 = ellipsoid. Parietal constriction teeth (Figure 7) 1 = two long lamella; 2 = two knob-shaped teeth; 3 = single knob-shaped tooth; 4 = no tooth. Basal constriction teeth (Figure 8) 1 = two knob-shaped teeth; 2 = no tooth. Tuba form (Figure 9) 1 = tuba type 1; 2 = tuba type 2; 3 = tuba type 3. Aperture view 1 = frontal view; 2 = right lateral view; 3 = back view; and 4 = left lateral view. Peristome types (Figure 11) 1 = simple peristome; 2 = double peristome. Spiral lines (Figure 12) 1 = thick and thin; 2 = thin only. Rib shape (Figure 13C) 1 = single-humped; 2 = slightly curved; 3 = straight. Rib thickness (Figure 13B) 1 = thick; 2 = thin.

Our shell shape characterisation approach that views the shell as a petrified ontogeny provides a set of distinguishable general characters for species delimitation (Table 3). Most of the species in this study can be identified just by using these general shape characters. Furthermore, those species that are not distinguishable with these general shape characters, are distinguishable by using more specific shell shape and size characters (see Taxonomy Key).

However, it is important to note that the intra- and inter-specific variation in shell shape is more difficult to characterise than the variation in shell size. This is reflected in our species description, where the variation in shell size and countable shell characters, such as ribs, but not the variation in general shell shape are explicitly given. Nevertheless, the general qualitative shell shape characters do implicitly reflect the variation because many of these, such as spire shape, tuba, and spiral lines, are obtained by categorising the quantitative variation of these characters (e.g. Figures 4 and 5).

### Conservation status assessment

Overall, we suggest that 10 of the non-Bornean *Plectostoma* species are threatened and *Plectostoma sciaphilum* is extinct. Specifically, *Plectostoma umbilicatum*, *Plectostoma senex*, *Plectostoma turriforme*, *Plectostoma retrovertens*, *Plectostoma charasense*, and *Plectostoma tenggekensis* are in the Critically Endangered category; *Plectostoma kubuensis* is in the Endangered category; and *Plectostoma dindingensis*, *Plectostoma palinhelix*, and *Plectostoma laidlawi* are in the Vulnerable category. All of these species, except *Plectostoma laidlawi*, occur in limestone hills in the State of Pahang, Malaysia, where many of these hills are being quarried or are at risk of being quarried. Our assessments would eventually be submitted to IUCN.

### Molecular phylogeny and COI barcoding

**Figures 16.**
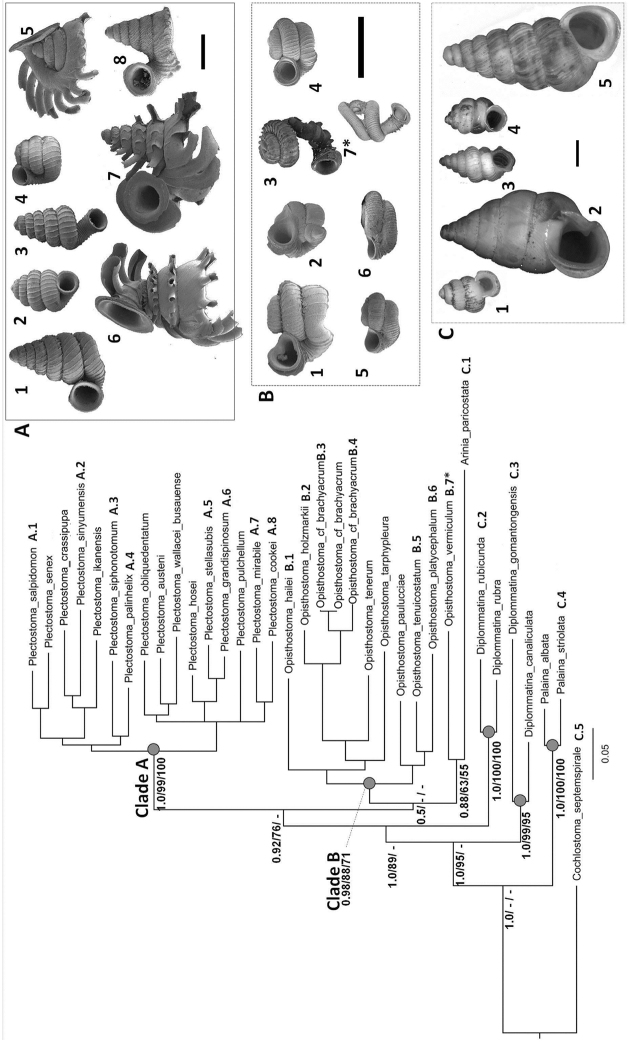
The phylogeny of Diplommatinidae genera with *Cochlostoma septemspirale* (Wagner, 1897) as outgroup. Bayesian inference 50% majority-rule consensus trees based on the concatenated dataset consisting of parts of 28S, 18S, COI, and 16S. Bayesian posterior probabilities, percent bootstrap support after 1000 maximum likelihood replicates, and percent bootstrap support after 1000 maximum parsimony replicates are shown for the major clades. Clade A consists of *Plectostoma* species, Clade B consists of *Opisthostoma* species. The shell forms are shown for representative taxa. Details of the taxa can be found in Table 1 (no. 1–28, 42, 48, 61, 69, 71, 73, and 78). Scale bar = 1 mm.

As revealed by the Bayesian posterior probability (PP) and maximum likelihood analysis bootstrap (BS) values of the phylogenetic tree in Figure 16, *Plectostoma* is monophyletic (BI/ML/PA; 1.0/99/100) and is the sister taxon of the less well-supported clade of *Opisthostoma* + *Arinia* (0.5/<70/<70). Within the *Opisthostoma* + *Arinia* clade, all *Opisthostoma* except *Opisthostoma vermiculum* form a well-supported clade (0.98/88/71). The divergence between these clades is similar to the divergence between other genera in Diplommatinidae. Our phylogenetic analysis suggested that *Opisthostoma vermiculum* Clements & Vermeulen, 2008 in Clements et al. (2008) has been incorrectly assigned to the genus *Opisthostoma*.

Table 4 shows the Kimura 2-parameter distances for all sequence pairs within each group and the net average between groups of sequences (for sequence alignment see Appendix 16). This reveals that all species pairs exceed a divergence of 10% (n = 169), with the exception of *Plectostoma crassipupa* vs. *Plectostoma christae* and *Plectostoma crassipupa* vs. *Plectostoma laidlawi*. Genetic divergence within each species is below 9% (n = 14, mean = 2.6%, SD = 0.1%), with the exception of *Plectostoma crassipupa*.

**Table 4.** COI sequence divergence † within and between 19 *Plectostoma* species.

Number of specimens	Divergence within groups ‡	Divergence between groups of sequences §																		
			*Plectostoma christae*	*Plectostoma crassipupa*	*Plectostoma davisoni*	*Plectostoma dindingensis*	*Plectostoma ikanensis*	*Plectostoma kakiense*	*Plectostoma kubuensis*	*Plectostoma laidlawi*	*Plectostoma mengaburensis*	*Plectostoma palinhelix*	*Plectostoma relauensis*	*Plectostoma retrovertens*	*Plectostoma salpidomon*	*Plectostoma senex*	*Plectostoma sinyumensis*	*Plectostoma siphonostomum*	*Plectostoma tohchinyawi*	*Plectostoma umbilicatum*
8	0.06	*Plectostoma christae*																		
3	0.13	*Plectostoma crassipupa*	0.09*																	
2	0.00	*Plectostoma davisoni*	0.15	0.12																
1	n.a.	*Plectostoma dindingensis*	0.15	0.14	0.20															
6	0.01	*Plectostoma ikanensis*	0.15	0.11	0.18	0.19														
1	n.a.	*Plectostoma kakiense*	0.14	0.11	0.17	0.21	0.17													
2	0.00	*Plectostoma kubuensis*	0.16	0.14	0.20	0.18	0.19	0.16												
3	0.08	*Plectostoma laidlawi*	0.12	0.09*	0.16	0.17	0.16	0.14	0.15											
2	0.01	*Plectostoma mengaburensis*	0.14	0.10	0.18	0.20	0.16	0.16	0.21	0.14										
2	0.00	*Plectostoma palinhelix*	0.13	0.12	0.18	0.15	0.18	0.15	0.20	0.15	0.16									
1	n.a.	*Plectostoma relauensis*	0.12	0.13	0.21	0.17	0.20	0.18	0.19	0.15	0.19	0.14								
1	n.a.	*Plectostoma retrovertens*	0.12	0.13	0.19	0.14	0.18	0.18	0.19	0.17	0.18	0.11	0.15							
5	0.04	*Plectostoma salpidomon*	0.11	0.11	0.15	0.14	0.16	0.16	0.19	0.14	0.15	0.12	0.15	0.12						
1	n.a.	*Plectostoma senex*	0.15	0.14	0.19	0.18	0.19	0.21	0.23	0.15	0.22	0.14	0.19	0.14	0.12					
2	0.00	*Plectostoma sinyumensis*	0.17	0.12	0.19	0.21	0.20	0.18	0.20	0.15	0.19	0.20	0.20	0.20	0.18	0.21				
5	0.03	*Plectostoma siphonostomum*	0.10	0.12	0.17	0.15	0.17	0.17	0.17	0.15	0.17	0.14	0.14	0.12	0.12	0.18	0.19			
2	0.00	*Plectostoma tohchinyawi*	0.13	0.12	0.18	0.16	0.17	0.18	0.20	0.15	0.18	0.12	0.17	0.13	0.06	0.10	0.21	0.14		
2	0.00	*Plectostoma umbilicatum*	0.16	0.13	0.19	0.21	0.19	0.17	0.20	0.16	0.18	0.19	0.20	0.19	0.18	0.22	0.13	0.19	0.20	
2	0.01	*Plectostoma whitteni*	0.15	0.14	0.18	0.16	0.21	0.21	0.23	0.18	0.20	0.15	0.18	0.14	0.11	0.11	0.21	0.15	0.10	0.21

† Analyses were conducted using the Kimura 2-parameter model (Kimura 1980). The analysis involved 51 nucleotide sequences. Codon positions included were 1st+2nd+3rd. All positions with less than 95% site coverage were eliminated. There were a total of 615 positions in the final dataset. These analyses were conducted in MEGA5 (Tamura et al. 2011).

‡ The number of base substitutions per site from averaging over all sequence pairs within each group.

§ The number of base substitutions per site from estimation of net average between groups of sequences. Genetic distances between two species smaller than 10% are shown in bold.

One key determinant for the success of DNA barcoding is prior knowledge of intra- and interspecific genetic distances for the barcoding marker in question. The optimum intra- and interspecific threshold in gastropods is higher than the conventional value (Hebert et al. 2003) of 3% (e.g. 4% in Davison et al. 2009, 6% in Köhler and Johnson 2012, 9.8%–25% in Parmakelis et al. 2013). In our study, we also found higher values of intraspecific variation and interspecific divergence’ of COI for three well-defined species, namely, *Plectostoma salpidomon*, *Plectostoma christae*, and *Plectostoma siphonostomum* are larger and smaller than 10%, respectively.

A study on a pulmonate limestone-dwelling micro-landsnail in the same region also suggests intraspecific COI divergence not exceeding 10% based on the Kimura 2-parameter model (Hoekstra and Schilthuizen 2011), and similar values were obtained for *Everettia*, a Malaysian pulmonate not restricted to limestone (Liew et al. 2009). Hence, based on our results, together with the only other two studies on the COI variation of land snails in Sundaland, we advise caution in using a conventional threshold value in COI genetic variability for species delimitation, when the background genetic variability of COI is unknown for a particular taxon in a particular geographic region.

### Key to Peninsular Malaysia *Plectostoma*

**Table:** 

1	Oblong apical spire	2
–	Depressed apical spire	10
2	Tuba with type 1 coiling	3
–	Tuba with type 2 or 3 coiling	4
3	Constriction with 2 parietal teeth. Aperture visible when shell observed in left lateral view	*Plectostoma siphonostomum*
–	Constriction without parietal tooth. Aperture visible when shell observed in back view	*Plectostoma umbilicatum*
4	Tuba with type 2 coiling. Aperture visible when shell observed in right lateral view	5
–	Tuba with type 3 coiling. Aperture visible when shell observed in left lateral view	8
5	Spire with both thick and thin spiral lines	6
–	Spire with only thin spiral lines	7
6	Left lateral side of outer peristome projected not more than two times the width of the right lateral side	*Plectostoma tohchinyawi*
–	Left lateral side of outer peristome projected more than three times the width of the right lateral side	*Plectostoma klongsangensis*
7	Constriction with 2 basal teeth	*Plectostoma charasense*
–	Constriction without basal tooth	*Plectostoma kitteli*
8	Constriction without basal tooth	*Plectostoma sciaphilum*
–	Constriction with 2 basal teeth	9
9	Constriction with 2 long lamella-shaped parietal teeth	*Plectostoma turriforme*
–	Constriction with knob-shaped parietal teeth	*Plectostoma senex*
10	Tuba with type 1 coiling	11
–	Tuba with type 2 or 3 coiling	15
11	Constriction with 1 or 2 parietal teeth	12
–	Constriction without parietal teeth	13
12	Constriction with 1 parietal tooth	*Plectostoma panhai*
–	Constriction with 2 parietal teeth	*Plectostoma christae*
13	Ellipsoid basal spire	*Plectostoma sinyumensis*
–	Ovoid basal spire	14
14	More than 1/4 of the tuba visible in top view	*Plectostoma mengaburensis*
–	Less than 1/4 of the tuba visible in top view	*Plectostoma dindingensis*
15	Tuba with type 3 coiling	16
–	Tuba with type 2 coiling	18
16	Simple peristome	*Plectostoma tonkinianum*
–	Double peristome	17
17	Aperture visible when shell observed in frontal view	*Plectostoma whitteni*
–	Aperture visible when shell observed in left lateral view	*Plectostoma crassipupa*
18	Aperture visible when shell observed in frontal or back views	19
–	Aperture visible when shell observed in right lateral view	24
19	Aperture visible when shell observed in back views	20
–	Aperture visible when shell observed in frontal views	21
20	Spire 1.6–1.9 mm high	*Plectostoma palinhelix*
–	Spire 2.3–2.6 mm high	*Plectostoma retrovertens*
21	Ovoid basal spire	22
–	Conical basal spire	23
22	Constriction with 1 parietal tooth	*Plectostoma relauensis*
–	Constriction with 2 parietal teeth	*Plectostoma davisoni*
23	Spire with both thick and thin spiral lines	*Plectostoma ikanensis*
–	Spire with only thin spiral lines	*Plectostoma kayiani*
24	Spire with straight radial ribs	25
–	Spire with slightly curved or single-humped radial ribs	28
25	Conical basal spire	26
–	Ovoid basal spire	27
26	Whorl periphery distinctly convex	*Plectostoma laidlawi*
–	Whorl periphery slightly convex	*Plectostoma annandalei*
27	Constriction without parietal teeth	*Plectostoma kubuensis*
–	Constriction with 2 parietal teeth	*Plectostoma kakiense*
28	Conical basal spire	29
–	Ovoid basal spire	30
29	Spire 1.6–1.7 mm high	*Plectostoma tenggekensis*
–	Spire 2.0–2.2 mm high	*Plectostoma praeco*
30	Whole tuba attaches to spire	*Plectostoma laemodes*
–	Less than 2/3 of the tuba attaches to spire	*Plectostoma salpidomon*

## Taxonomy

### Genus Plectostoma

http://species-id.net/wiki/Plectostoma

Plectostoma Adam 1865: 177. Type species: *Plectostoma DeCrespignii* (by original designation)
Geothauma Crosse 1892: 282. Type species (by original designation): *Plectostoma grandispinosum* (Godwin-Austen, 1889).

#### Generic classification dispute.

The genus *Opisthostoma* was described by Blanford and Blanford (1860) based on one species–*Opisthostoma nilgiricum* from India. Adam (1865b) described a second species of *Opisthostoma*, namely, *Opisthostoma decrespignyi*, which he previously described under the new genus *Plectostoma* (Adam 1865a). Nevertheless, Blanford (1867) concluded that the conchological differences between these two taxa were not enough to create a different genus. Instead, he suggested these could be two different subgenera. Next, another two subgenera–*Gyrostropha* Ancey, 1887 and *Geothauma* Crosse, 1892, were proposed for different forms of *Opisthostoma* and *Plectostoma*. However, Smith (1893a) suggested that this subgeneric classification was not necessary until more data other than shell morphology were available. Since then, a classification into three subgenera within the genus *Opisthostoma*, namely, *Geothauma*, *Opisthostoma*, and *Plectostoma* has generally been accepted (e.g. von Martens and Thiele 1908, van Benthem Jutting 1932, van Benthem Jutting 1952), until, in a recent review of the genus *Opisthostoma*, Vermeulen (1991, 1994) followed a classification into only two subgenera, namely, *Opisthostoma* and *Plectostoma*.

#### Diagnosis.

**Figures 17.**
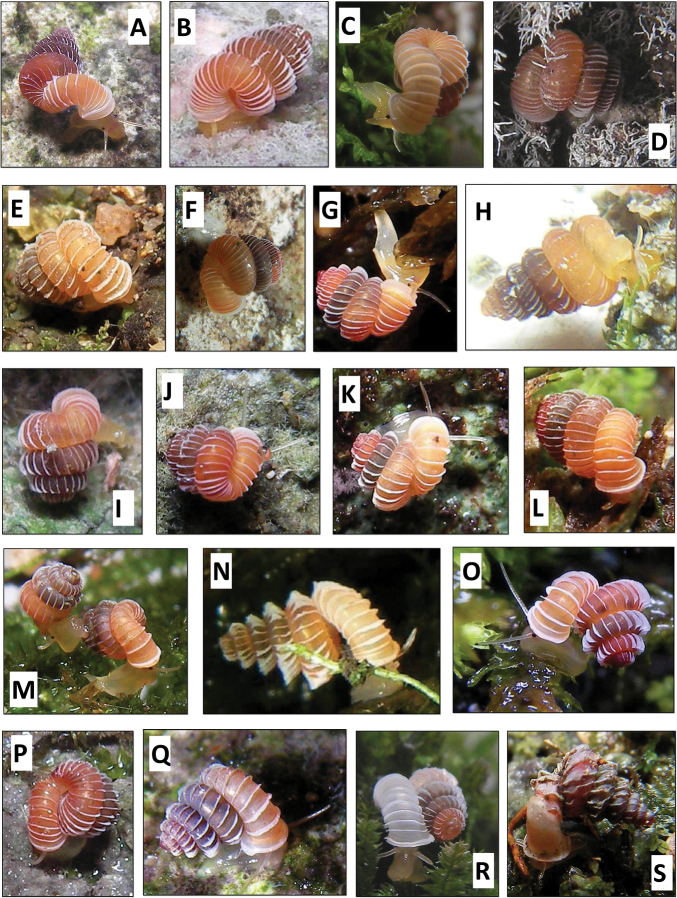
Photographs of 17 living *Plectostoma* species. **A** *Plectostoma salpidomon* (van Benthem Jutting, 1952), BOR 5569 **B** *Plectostoma umbilicatum* (van Benthem Jutting, 1952), BOR 5503 **C** *Plectostoma palinhelix* (van Benthem Jutting, 1952), BOR 5520 **D** *Plectostoma kakiense* (Tomlin, 1948), BOR 5516 **E** *Plectostoma mengaburensis* sp. n., BOR 5574 **F** *Plectostoma kubuensis* sp. n., BOR 5518 **G** *Plectostoma whitteni* sp. n., BOR 5536 **H** *Plectostoma senex* (van Benthem Jutting, 1952), BOR 5631 **I** *Plectostoma crassipupa* (van Benthem Jutting, 1952), BOR 5515 **J** *Plectostoma ikanensis* sp. n. Form BOR 5507, BOR 5507 **K** *Plectostoma ikanensis* sp. n. Form BOR 5504, BOR 5504 **L** *Plectostoma sinyumensis* (Maassen, 2001), BOR 5537 **M** *Plectostoma crassipupa* (van Benthem Jutting, 1952), BOR 5512 **N** *Plectostoma siphonostomum* (van Benthem Jutting, 1952), BOR 5557 **O** *Plectostoma laidlawi* (Sykes, 1902) Form BOR 5510, BOR 5510 **P** *Plectostoma relauensis* sp. n., BOR 5511 **Q** *Plectostoma christae* (Maassen, 2001), BOR 5505 **R** *Plectostoma retrovertens* (Tomlin, 1938), BOR 5559 **S** *Plectostoma tohchinyawi* sp. n., BOR 5533.

Despite the distinct ecological niche differences (see below–Distribution and habitat) between *Opisthostoma* and *Plectostoma*, it is not feasible to use this criterion in the genus identification, because information about the ecology is usually not available as most collections are made by soil sampling. After 150 years of work on *Opisthostoma*, it is still difficult to identify reliable apomorphic character states that can be used to distinguish between *Opisthostoma* and *Plectostoma* (Vermeulen 1991, 1994). Both share the character state of the constriction, which is a slight shrinkage in the whorl towards the end of the spire. When the animal retracts into its shell, its operculum rests at the constriction (Vermeulen 1991). It is, however, possible to make a morphological distinction between *Opisthostoma* and *Plectostoma* on the basic of the shell colouration in a fully grown adult, which is orange or pinkish in *Plectostoma* and white or pale yellowish in *Opisthostoma*. The colour differences between these two genera are very clear when comparing the living snails or freshly dead shell material (Figure 17, and Appendix 18). Some *Plectostoma* species have a regularly coiled tuba, and a shell form that is similar to *Arinia*. However, *Plectostoma* and *Arinia* can be easily distinguished by shell colour differences. The shell colour in *Arinia* is similar to that in *Opisthostoma*.

#### Description.

**Apex.** Protoconch is either slightly, moderately or distinctly convex (Figure 3).

**Spire.** Height: 1.0 mm–3.7 mm. Width: 0.85 mm–2.60 mm (Figure 11). Number of whorls between 2 3/4–71/4. Apical spire shape: oblong or depressed conical (Figure 4). Basal spire shape: conical, ovoid or ellipsoid (Figure 5). Whorl periphery: flat, slightly, moderately or distinctly convex. Umbilicus: open, partially closed, or totally closed.

**Constriction.** Parietal teeth: parietal side of inner constriction whorl (Figure 2) with two long lamellae (Figure 6A), two ridges with a knob at each end (Figure 6B), one ridge with a knob at one end (Figure 6C), or no teeth (Figure 6D). Basal teeth: basal side of inner constriction whorl (Figure 2) with no teeth (Figure 7B), one ridge running parallel with the whorl growing direction, one ridge with a knob at one end running perpendicular to the whorl growing direction, or a combination of the latter two types (Figure 7A).

**Tuba.** Coiling direction: regular coiling (type 1, Figure 8A) or distorted (Type 2, or 3) (Figure 8B and C). Tuba whorl length in proportion to spire last whorl: *ca.* 3/8–1 1/2. Proportion of tuba that attaches to spire: whole to none.

**Aperture and peristome.** Peristome: simple aperture without outer peristome (Figure 10B), or double peristome (Figure 10C and 10D). Shape of outer peristome (Figure 10A): same as inner peristome and uniformly round, or highly projected or slightly projected at either a particular side or at a several sides of anterior, poteriorior, left and right laterial (Figure 9 and 10A).

**Spiral lines.** Either thick or thin, or only thin lines present (Figure 12).

**Radial ribs.** Rib density: 4–32 per mm on the spire’s last whorl in right lateral view (Figure 13A). Intensity: thick or thin (Figure 13B). Shape: straight, slightly curved, distinctly curved, single humped, single looped or double looped and the shape remaining the same or changing between between the spire and the tuba (Figure 13C, but single-looped, and double-humped not shown). Inclination: from orthoclin to prosoclin.

#### Distribution and habitat.

**Figures 18.**
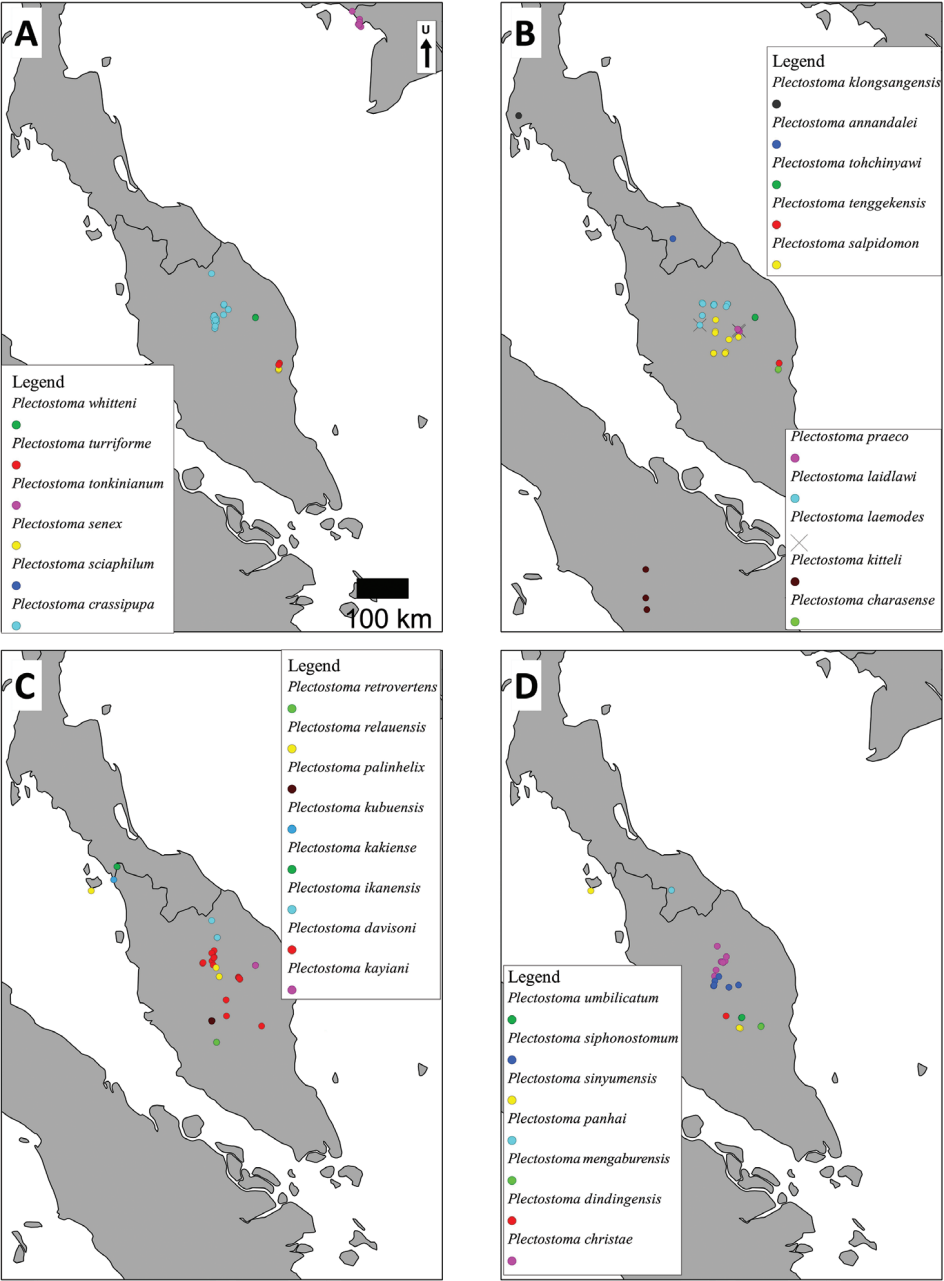
Distributional records of *Plectostoma* species. **A**–**D** distribution records for each of 31 *Plectostoma* species.

The distribution range of *Plectostoma* is about 4.6 million square kilometres within the extent limited by 11°N, 97°E and 5°S, 120°E. However probably less than 5% of this large area is covered by limestone outcrops where suitable habitat may exist for obligate karst taxa like *Plectostoma*. The genus counts 79 species and occurs in Vietnam (1 species), Thailand (1), Peninsular Malaysia (28), Sumatra (1), and Borneo (48). Peninsular Malaysia, Sumatra and Borneo are part of the biogeographical region called Sundaland (Johnson 1964). *Plectostoma* is found on most limestone hills. However, the genus is conspicuously absent on the limestone hills to the west of the central mountain ranges, such as the hills in the States of Perak and Kedah in Peninsular Malaysia, and in the northwestern half of Sumatra (Figure 18). No species have been recorded from the east coast of Sumatra, where hardly any limestone outcrops exist.

Based on collection data and our field experience, there is a distinct ecological divergence between *Plectostoma* and *Opisthostoma*. This was already observed in the 19^th^ century (Blanford and Blanford 1860, de Crespigny 1865, Blanford 1866), and also by Berry (1961). *Plectostoma* can only be found in limestone outcrops, where the rock face is its major habitat, although a few individuals can occasionally be found on vegetation debris below the limestone rock face. *Opisthostoma*, on the other hand, is a soil dweller, living in leaf litter on the forest floor. They are mostly but not exclusively found in forest over limestone bedrock (Schilthuizen et al. 2003b).

#### Phylogenetic relationships.

Our molecular phylogenetic analysis reveals that *Plectostoma*, *Opisthostoma*, and *Arinia* are phylogenetically closely related (Figure 16). It is important to point out that the phylogenetic relationships among *Plectostoma*, *Opisthostoma* (except *Opisthostoma vermiculum*), *Opisthostoma vermiculum*, and *Arinia* are unresolved. Figure 16 shows representative shell morphologies of the taxa that were included in the phylogenetic analysis, and it is clear that it is rather difficult to find shared derived characteristics (synapomorphies) in size, spire shape, or tuba coiling regime, for either *Opisthostoma* or *Plectostoma*.

Nonetheless, we treat *Plectostoma* and *Opisthostoma* as two separate genera based on their ecological divergence and differences in adult shell colouration. Similarly, we propose that *Opisthostoma vermiculum* and *Arinia* should be considered as two separate genera. However, this hypothesis needs further testing with more genetic data from *Opisthostoma vermiculum* Clements & Vermeulen, 2008 (in Clements et al. 2008), the conchologically similar *Opisthostoma gittenbergeri* Vermeulen & Clements, 2008 and further *Arinia* species.

### Plectostoma dindingensis sp. n.

http://zoobank.org/529D652A-B50B-4B19-BD5D-0D769F9EBC4F

http://species-id.net/wiki/Plectostoma_dindingensis

**Figures 19.**
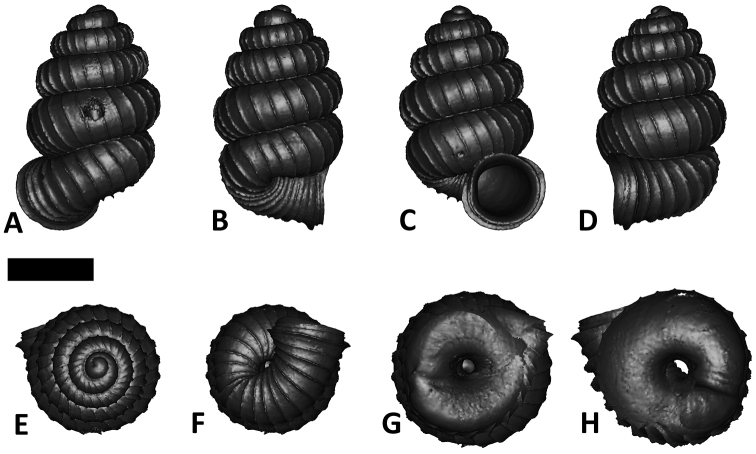
*Plectostoma dindingensis* sp. n. BOR 5642, Holotype. **A** frontal view **B** left lateral view **C** back view **D** right lateral view **E** top view **F** bottom view **G** parietal part of constriction inner whorl **H** basal part of constriction inner whorl. Scale bar = 1 mm (for **A–F**).

Figure 19 , Appendix 7

#### Type material.

Holotype: BOR 5642(1). Paratypes: BOR 5612(3)

#### Diagnosis.

Shares with *Plectostoma mengaburensis* and *Plectostoma panhai* the general shell form, in terms of apex, spire and tuba, but differs by lacking constriction teeth and having a more tightly coiled tuba (less than 1/4 of the tuba visible in top view).

#### Etymology.

This species is named after its type locality–Dinding.

#### Description.

**Apex.** Shape: moderately convex.

**Spire.** Height: 1.8–1.9 mm. Width: 1.4–1.5 mm. Number of whorls: 3 7/8–4 5/8. Apical spire shape: depressed conical. Basal spire shape: ovoid. Whorl periphery: distinctly convex. Umbilicus: partially closed by tuba.

**Constriction.** Parietal teeth: none. Basal teeth: none.

**Tuba.** Coiling direction: type 1 and aperture visible from back view. Tuba whorl length in proportion to spire last whorl: *ca.* 5/8–3/4. Proportion of tuba that attaches to the spire: whole.

**Aperture and peristome.** Peristome: double peristomes. Outer peristome shape: same as inner peristome and uniformly projected all around, except the posterior part.

**Spiral lines.** Thick lines: present. Thin lines: present.

**Radial ribs.** Rib density: 6–8 ribs per mm. Rib intensity: thin. Shape: straight. Inclination: orthoclin.

#### Distribution.

Type locality. The exact location is unknown. The specimens are labeled as collected from “dinding”. It could be near Kampung Bukit Dinding, Pahang (3°49'41"N, 102°22'3"E).

Distribution range. The species has only been recorded from the type locality (Figure 18D).

#### Conservation status.

Vulnerable (D2 ver. 10.1). The samples was collected from a living population in 1997. The population status remains unclear. The area around Kampung Bukit Dinding has been converted to plantation and no significant undisturbed forest coverage remains.

#### Discussion.

See discussion under *Plectostoma christae*.

### Plectostoma mengaburensis sp. n.

http://zoobank.org/3C81401C-9C4C-44C2-9A44-9F2F115D8D37

http://species-id.net/wiki/Plectostoma_mengaburensis

**Figures 20.**
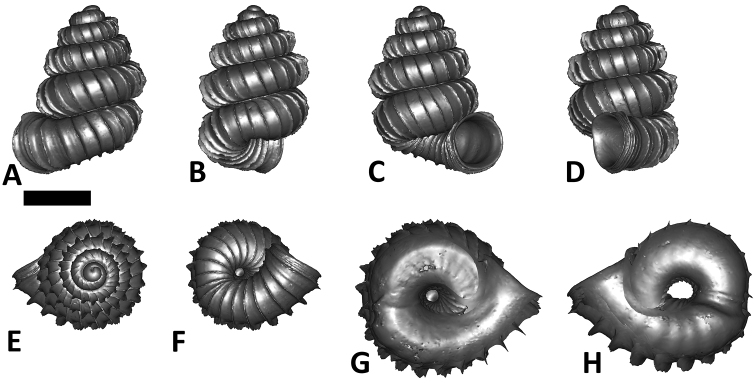
*Plectostoma mengaburensis* sp. n. BOR 5643. **A** frontal view **B** left lateral view **C** back view **D** left lateral view **E** top view **F** bottom view **G** parietal part of constriction inner whorl **H** basal part of constriction inner whorl. Scale bar = 1 mm (for **A–F**).

Figures 17E , 20 , Appendix 7

#### Type material.

Holotype: BOR 5643(1). Paratypes: BOR 5574(>25), V8822 (6)

#### Diagnosis.

Shares with *Plectostoma dindingensis* and *Plectostoma panhai* the general shell form, in the terms of apex, spire and tuba, but differs by lacking constriction teeth and having a less tightly coiled tuba (more than 1/4 of the tuba visible in top view).

#### Etymology.

This species is named after its type locality–Mengabur.

#### Description.

**Apex.** Shape: moderately convex.

**Spire.** Height: 1.9–2.2 mm. Width: 1.4–1.6 mm. Number of whorls: 3 7/8–4 1/2. Apical spire shape: depressed conical. Basal spire shape: ovoid. Whorl periphery: distinctly convex. Umbilicus: open or half of the umbilicus closed by tuba.

**Constriction.** Parietal teeth: none. Basal teeth: none.

**Tuba.** Coiling direction: type 1 and aperture visible from back view. Tuba whorl length in proportion to spire last whorl: *ca.* 5/8. Proportion of tuba that attaches to spire: whole.

**Aperture and peristome.** Peristome: double peristomes. Outer peristome shape: same as inner peristome and uniformly projected all around, except the posterior part.

**Spiral lines.** Thick lines: present. Thin lines: present.

**Radial ribs.** Rib density: 7 ribs per mm. Rib intensity: thin. Shape: straight. Inclination: orthoclin.

#### Distribution.

Type locality. An unnamed small limestone hill in the plantation near the large Bukit Mengabur quarry (3°43'50"N, 102°49'40"E).

Distribution range. This species only occurs in the Mengabur limestone cluster (Figure 18D).

#### Conservation status.

Near Threatened. This species only occurs in the Mengabur limestone cluster, which is quite large (*ca.* 10 km^2^, estimated from Google Earth), and its vegetation cover is largely undisturbed. However, quarrying activities have started at the eastern part of the cluster and the whole limestone cluster is surrounded by plantation.

#### Discussion.

See discussion under *Plectostoma christae*.

### Plectostoma sinyumensis

(Maassen, 2001)

http://species-id.net/wiki/Plectostoma_sinyumensis

**Figures 21.**
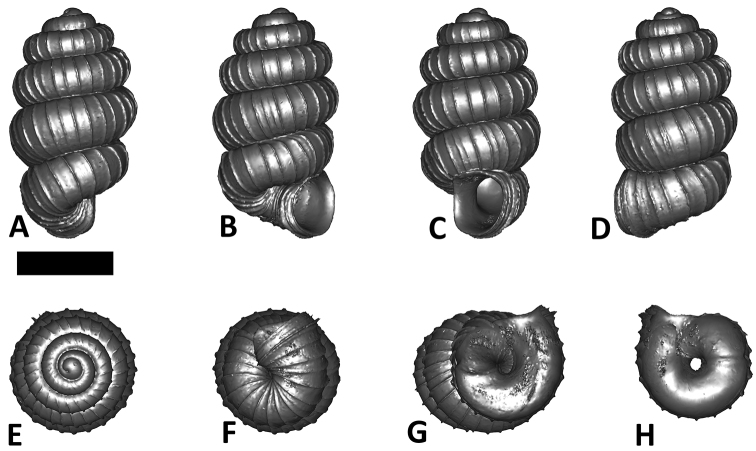
*Plectostoma sinyumensis* (Maassen, 2001) BOR5537. **A** frontal view **B** left lateral view **C** back view **D** right lateral view **E** top view **F** bottom view **G** parietal part of constriction inner whorl **H** basal part of constriction inner whorl. Scale bar = 1 mm (for **A–F**).

Figures 17L , 21 , Appendix 7

Opisthostoma sinyumensis Maassen, 2001: 52, figures 1, 6 & 7 (original description).
Opisthostoma sinyumensis Maassen, Clements (2007: 74).
Opisthostoma sinyumensis Maassen, Clements et al. (2008: 2760).
Opisthostoma sinyumensis Maassen, Webster et al. (2012: 628).

#### Type material.

Holotype: ZMA 138439(1) (Seen). Paratypes: ZMA 138440(>25) (Seen), RMNH 81804(2) (Seen).

#### Other examined materials.

BOR 462(5), BOR 5537(>10), BOR 5623(>50).

#### Diagnosis.

Shares with *Plectostoma dindingensis*, *Plectostoma mengaburensis*, *Plectostoma christae*, and *Plectostoma panhai* the general shell form, in terms of apex, apical spire and tuba, but differs by having an ellipsoid basal spire.

#### Description.

**Apex.** Shape: moderately convex.

**Spire.** Height: 1.6–1.9 mm. Width: 1.2–1.3 mm. Number of whorls: 3–3 3/4. Apical spire shape: depressed conical. Basal spire shape: ellipsoid. Whorl periphery: distinctly convex. Umbilicus: almost completely closed by tuba.

**Constriction.** Parietal teeth: none. Basal teeth: none.

**Tuba.** Coiling direction: type 1 and aperture visible between right lateral and back view. Tuba whorl length in proportion to spire last whorl: *ca.* 5/8–3/4. Proportion of tuba that attaches to spire: whole.

**Aperture and peristome.** Peristome: double peristomes. Outer peristome shape: same as inner peristome and uniformly projected all around, except the posterior part.

**Spiral lines.** Thick lines: absent. Thin lines: present.

**Radial ribs.** Rib density: 9–10 ribs per mm. Rib intensity: thin. Shape: straight. Inclination: orthoclin.

#### Distribution.

Type locality. Gunung Senyum, Pahang (3°42'35"N, 102°26'3"E).

Distribution range. In addition to the type locality, this species also was found at the Jebak Puyuh limestone outcrop, which lies about 1 km east of Gunung Senyum (Figure 18D). BOR 462 was collected in Pulau Singa Besar, which is located about 300 km from type locality. The reliability of the collection data is dubious (the same was found for BOR 463 of *Plectostoma relauensis*).

#### Conservation status.

Near Threatened. There are four limestone hills in this cluster. Gunung Senyum and Jebak Puyuh are the larger among these four hills. The former is gazetted as recreation forest but the latter has been at risk of destruction. Jebak Puyuh had been earmarked for quarrying several years ago, but the plan has been abandoned. *Plectostoma sinyumensis* has been recorded from these two hills, but its status at the two smaller hills remains unknown. In a survey in July 2010, a living population was recorded at Jebak Puyuh, in an enclosed humid sinkhole. Intensive surveying on the limestone rock faces of Gunung Senyum and other parts of Jebak Puyuh has failed to retrieve any additional living individuals, probably because most of the rock faces were very dry.

#### Discussion.

See discussion under *Plectostoma christae*.

### Plectostoma umbilicatum

(van Benthem Jutting, 1952)

http://species-id.net/wiki/Plectostoma_umbilicatum

Figure 17B , 22 , Appendix 8

Opisthostoma umbilicatum van Benthem Jutting, 1952: 49, figure 25 (original description).
Opisthostoma umbilicatum van Benthem Jutting, van Benthem Jutting (1961: 39).
Opisthostoma umbilicatum van Benthem Jutting, Clements (2007: 74).
Opisthostoma umbilicatum van Benthem Jutting, Clements et al. (2008: 2760).

#### Type material.

Holotype: ZMA 136070(1) (Seen). Paratypes: ZMA 136071(8) (Seen).

#### Other examined materials.

BOR 5503(>10), BOR 5625(>25).

#### Diagnosis.

Shares with *Plectostoma dindingensis*, *Plectostoma mengaburensis*, and *Plectostoma panhai* the tuba form, but differs by having an oblong conical apical spire.

#### Description.

**Apex.** Shape: moderately convex.

**Spire.** Height: 2.0–2.3 mm. Width: 1.3–1.5 mm. Number of whorls: 4 5/8–4 3/8. Apical spire shape: oblong conical. Basal spire shape: ovoid. Whorl periphery: distinctly convex. Umbilicus: completely open or partially closed by tuba.

**Constriction.** Parietal teeth: none. Basal teeth: none.

**Tuba.** Coiling direction: type 1 and aperture visible from back view. Tuba whorl length in proportion to spire last whorl: *ca.* 1/2–3/4. Proportion of tuba that attaches to spire: whole.

**Aperture and peristome.** Peristome: double peristomes. Outer peristome shape: same as inner peristome and uniformly projected all around, except the posterior part.

**Spiral lines.** Thick lines: present. Thin lines: present.

**Radial ribs.** Rib density: 6–9 ribs per mm. Rib intensity: thin. Shape: straight. Inclination: orthoclin.

**Figures 22.**
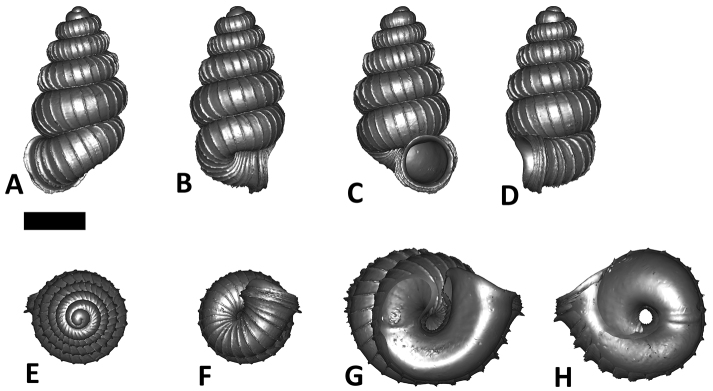
*Plectostoma umbilicatum* (van Benthem Jutting, 1952) BOR5503. **A** frontal view **B** leftlateral view; **C** back view **D** right lateral view **E** top view **F** bottom view **G** parietal part of constriction inner whorl **H** basal part of constriction inner whorl. Scale bar = 1 mm (for **A–F**).

#### Distribution.

Type locality. Limestone hill cluster named Kota Tongkat (3°53'28"N, 102°28'23"E).

Distribution range. It is only known from the type locality. All other adjacent limestone outcrops have been sampled, but only other *Plectostoma* species were found (Figure 18D).

#### Conservation status.

Critically Endangered (B2ab(iii)+C2a(i) ver. 10.1). The Kota Tongkat limestone cluster is surrounded by oil palm plantation and heavily degraded forest. This species is only known from this limestone cluster. Recent soil samplings have not revealed any recent dead shells (Clements et al. 2008). Neverthelss, we found a living population with fewer than 100 individuals at a wet stalagmite of the entrance of one the caves during an intensive survey in May 2011. During that survey, we noticed that all other rock surfaces of the limestone outcrops in Kota Tongkat were very dry. Thus, the recorded and other unknown living populations are at risk of extinction because a long drought might wipe them out.

#### Discussion.

See discussion under *Plectostoma christae*.

### Plectostoma siphonostomum

(van Benthem Jutting, 1952)

http://species-id.net/wiki/Plectostoma_siphonostomum

Figure 17N , 23 , Appendix 8

Opisthostoma siphonostomum van Benthem Jutting, 1952: 52, figure 27 (original description).
Opisthostoma siphonostomum van Benthem Jutting, van Benthem Jutting (1961: 39).
Opisthostoma siphonostomum van Benthem Jutting, Berry (1964: 203).

#### Type material.

Holotype: ZMA 136054(1) (Seen). Paratype: ZMA 136055(8) (Seen).

#### Other examined materials.

ZMA 162138(>100), BOR 5513 (>25), BOR 5521(>10), BOR 5557(>10), V 8199(>50), V 8223(>25).

#### Diagnosis.

Shares with *Plectostoma christae* the tuba form, but differs by having an oblong conical apical and conical basal spire.

#### Description.

**Apex.** Shape: distinctly convex.

**Spire.** Height: 1.9–2.1 mm. Width: 1.2–1.5 mm. Number of whorls: 4 3/4–5. Apical spire shape: oblong conical. Basal spire shape: conical. Whorl periphery: distinctly convex. Umbilicus: Open.

**Constriction.** Parietal teeth: two. Basal teeth: none.

**Tuba.** Coiling direction: type 1 and aperture visible between right lateral and back view. Tuba whorl length in proportion to spire last whorl: *ca.* 3/8–1/2. Proportion of tuba that attaches to spire: at least 1/2.

**Aperture and peristome.** Peristome: double peristomes. Outer peristome shape: same as inner peristome and uniformly projected all around, except the posterior part.

**Spiral lines.** Thick lines: present. Thin lines: present.

**Radial ribs.** Rib density: 7–8 ribs per mm. Rib intensity: thin. Shape: slightly curved. Inclination: orthoclin.

**Figures 23.**
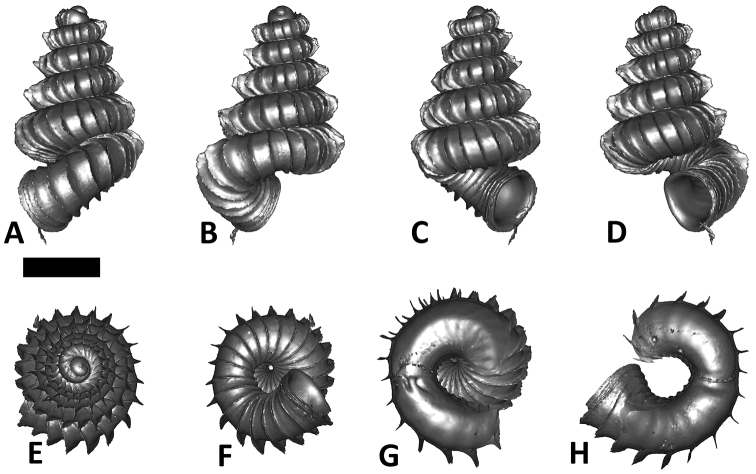
*Plectostoma siphonostomum* (van Benthem Jutting, 1952) BOR5539. **A** frontal view **B** left lateral view **C** back view **D** right lateral view **E** top view **F** bottom view **G** parietal part of constriction inner whorl **H** basal part of constriction inner whorl. Scale bar = 1 mm (for **A–F**).

#### Distribution.

Type locality. Gua Siput, Taman Negara, Pahang (4°26'47"N, 102°14'44"E).

Distribution range. *Plectostoma siphonostomum* has a similar distribution pattern as *Plectostoma salpidomon* and often occurs sympatrically with that species. It can be found in many limestone outcrops in the valley between the Titingwangsa Range, Tahan Range and Benom Range (Figure 18D).

#### Conservation status.

Least concern. Living populations of *Plectostoma siphonostomum* were recorded at several limestone hills during surveys between 2010 and 2011. Several of these are located within the National Park.

#### Discussion.

See discussion under *Plectostoma christae*.

### Plectostoma panhai

(Maassen, 2001)

http://species-id.net/wiki/Plectostoma_panhai

**Figures 24.**
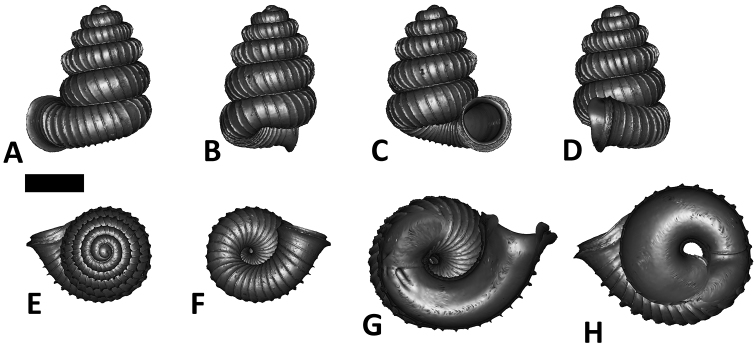
*Plectostoma panhai* (Maassen, 2001) ZMA 138438. **A** frontal view **B** left lateral view **C** back view **D** right lateral view **E** top view **F** bottom view **G** parietal part of constriction inner whorl **H** basal part of constriction inner whorl. Scale bar = 1 mm (for **A–F**).

Figure 24 , Appendix 7

Arinia panhai Maassen, 2001: 55, figure 4, 12 & 13 (original description).

#### Type material.

Holotype: RMNH 81809(1) (Seen). Paratypes: RMNH 81810(2) (Seen).

#### Other examined materials.

ZMA 138438(1).

#### Diagnosis.

Shares with *Plectostoma mengaburensis* and *Plectostoma dindingensis* the general shell form, in the terms of apex, spire and tuba, but differs by having a single parietal constriction tooth.

#### Description.

**Apex.** Shape: moderately convex.

**Spire.** Height: 2.1–2.2 mm. Width: 1.6–1.7 mm. Number of whorls: 4 1/2–4 5/8. Apical spire shape: depressed conical. Basal spire shape: ovoid. Whorl periphery: moderately convex. Umbilicus: open.

**Constriction.** Parietal teeth: one. Basal teeth: none.

**Tuba.** Coiling direction: type 1 and aperture visible from back view. Tuba whorl length in proportion to spire last whorl: *ca.* 3/4. Proportion of tuba that attaches to spire: whole.

**Aperture and peristome.** Peristome: double peristomes. Outer peristome shape: same as inner peristome and uniformly projected all around, except the posterior part.

**Spiral lines.** Thick lines: present. Thin lines: present.

**Radial ribs.** Rib density: 8–9 ribs per mm. Rib intensity: thin. Shape: straight. Inclination: orthoclin.

#### Distribution.

Type locality. Tham Krachaeng, Thailand (6°12'50"N, 101°12'9"E). The location description in the original publication is not completely correct “(06°55'022"N, 101°12'160"E)” (Maassen 2001).

Distribution range. This species is only known from the type locality (Figure 18D).

#### Conservation status.

Data Deficient.

#### Discussion.

See discussion under *Plectostoma christae*.

### Plectostoma christae

(Maassen, 2001)

http://species-id.net/wiki/Plectostoma_christae

**Figures 25.**
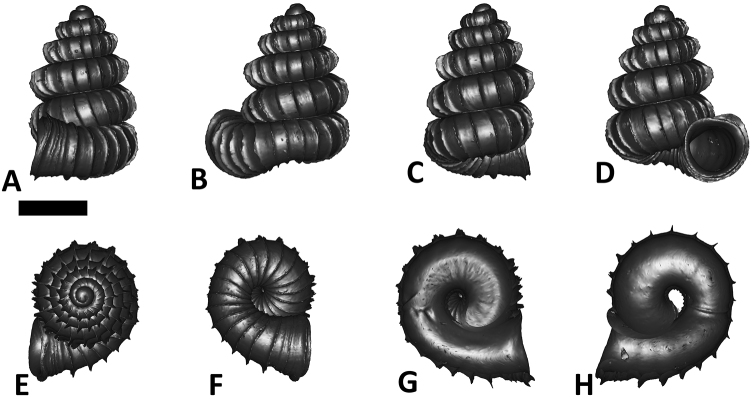
*Plectostoma christae* (Maassen, 2001) BOR 5572. **A** frontal view **B** left lateral view **C** back view **D** right lateral view **E** top view **F** bottom view **G** parietal part of constriction inner whorl **H** basal part of constriction inner whorl. Scale bar = 1 mm (for **A–F**).

Figures 17Q , 25 , Appendix 6

Opisthostoma christae Maassen, 2001: 52, figures 3, 10 & 11 (original description).
Opisthostoma jensi Maassen, 2001: 56, figures 5, 14 & 15 (original description), syn. n.

#### Type material.

Holotype: RMNH 81805(1) (Seen). Paratypes: RMNH 81806(1) (Seen).

#### Other examined materials.

RMNH 81807(1), RMNH 81808(1), ZMA 138436(1), ZMA 138437(2), BOR 3496(1), BOR 5505(>25), BOR 5506(>50), BOR 5509(>25), BOR 5572(2), V 12702(1), V 8314(>25), V 8406(2), V 9153(2), V 9207(>100), V 9285(3).

#### Diagnosis.

Shares with *Plectostoma dindingensis*, *Plectostoma mengaburensis*, and *Plectostoma panhai* the general shell spire form but differs by having two parietal constriction teeth and aperture visible when shell observed in left lateral view.

#### Description.

**Apex.** Shape: moderately convex.

**Spire.** Height: 1.9–2.6 mm. Width: 1.4–1.8 mm. Number of whorls: 3 5/8–4 1/2. Apical spire shape: depressed conical. Basal spire shape: ovoid. Whorl periphery: distinctly convex. Umbilicus: open.

**Constriction.** Parietal teeth: two. Basal teeth: none.

**Tuba.** Coiling direction: type 1 and aperture visible from left lateral view. Tuba whorl length in proportion to spire last whorl: *ca.* 3/8–5/8. Proportion of tuba that attaches to spire: whole.

**Aperture and peristome.** Peristome: double peristomes. Outer peristome shape: same as inner peristome and uniformly projected all around, except the posterior part.

**Spiral lines.** Thick lines: absent. Thin lines: present.

**Radial ribs.** Rib density: 5–6 ribs per mm. Rib intensity: thin. Shape: straight. Inclination: orthoclin.

#### Distribution.

Type locality. Limestone hills, 16 km west of Gua Musang (4°54'46"N, 102°6'22"E).

Distribution range. Limestone hills along the 50 km Northeast transect between 4°38'51"N, 101°58'58"E and 5°0'13"N, 102°11'59"E (Figure 18D).

#### Conservation status.

Near Threatened. Until today, this species has been recorded from at least six limestone hills. In a field survey in 2011 and 2012, living populations of *Plectostoma christae* could be found on four of these hills. All of these are located near the road and are surrounded by oil palm plantation, although there is no immediate threat.

#### Discussion.

*Plectostoma christae*, together with *Plectostoma dindingensis*, *Plectostoma mengaburensis*, *Plectostoma sinyumensis*, *Plectostoma umbilicatum*, *Plectostoma siphonostomum*, and *Plectostoma panhai* represent a group of *Plectostoma* species that have a regulary coiled tuba (type 1 tuba). The species of this group occur only in Peninsular Malaysia and are genetically highly divergent (> 10% differences in COI) from the others (Table 4). All of the seven species are distributed allopatrically (Figure 18D).

We synonymised *Plectostoma jensi* with *Plectostoma christae*, both of which were described from the same locality. Maassen (2001) distinguished between them by the slight difference in umbilicus opening and aperture tilting. In the material at our disposal, we recognised that these differences are intrapopulational variation. All individuals share the same diagnostic shell characters as mentioned above. In addition to the morphological evidence, the genetic variation between individuals with different shell forms is smaller than our species delimitation threshold of 10%.

Two species of this group, namely *Plectostoma christae* and *Plectostoma siphonostomum*, have a wider distribution range than other species in this group. The two species occur parapatrically on the limestone hills in the centre of Peninsular Malaysia (Figure 18D). On the other hand, very little is known of the distribution range of *Plectostoma panhai*. Although this species was reported only once and only from the type locality, it might also occur at other limestone sites near the type locality. *Plectostoma panhai* is very similar to *Plectostoma christae*, but the two are separated by more than 150 km, and the limestone hills in between are occupied by three other *Plectostoma* species. The disjunct distribution and its single constriction tooth support the decision that *Plectostoma panhai* is a distinct species from *Plectostoma christae*.

The remaining four species of this group, namely, *Plectostoma sinyumensis*, *Plectostoma mengaburensis*, *Plectostoma dindingensis*, and *Plectostoma umbilicatum*, are site endemics, occurring at each of the four small limestone clusters in the centre of Peninsular Malaysia (Figure 18D). These clusters are each quite isolated, with no other limestone hills within a 20 km radius.

Although these four species occur in adjacent limestone limestone clusters, and they have similar shell shapes, their taxonomic status are clear. The COI sequence divergence between these species is larger than 13% and each of them has a set of diagnostic shell characters (Tables 3 and 4). This may raise the question how each species evolved in each limestone cluster and how long these four species have been isolated. For example, a neighbouring species, *Plectostoma salpidomon*, has a similar distribution range as the former four species, but the morphological and genetic divergence is much smaller than in these four species. Presumably, the answer lies in the details of the geomorphological evolution of the limestone outcrops, which, however, remains largely unknown.

### Plectostoma crassipupa

(van Benthem Jutting, 1952)

http://species-id.net/wiki/Plectostoma_crassipupa

**Figures 26.**
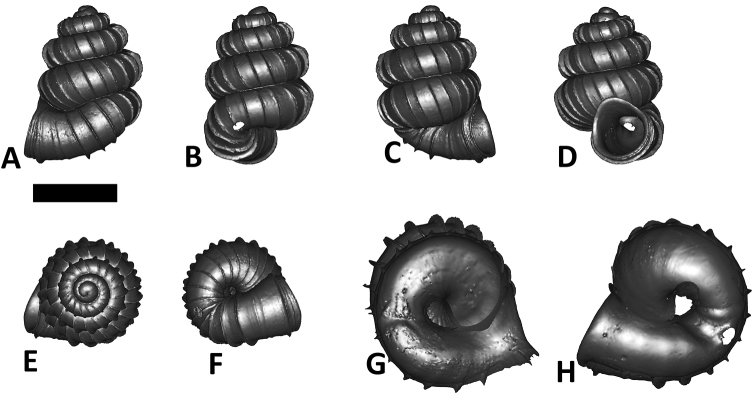
*Plectostoma crassipupa* (van Benthem Jutting, 1952) BOR 5512. **A** frontal view **B** left lateral view **C** back view **D** left lateral view **E** top view **F** bottom view **G** parietal part of constriction inner whorl **H** basal part of constriction inner whorl. Scale bar = 1 mm (for **A–F**).

Figures 17I, M , 26 , Appendix 6

Opisthostoma crassipupa van Benthem Jutting, 1952: 51, figure 26 (original description).
Opisthostoma crassipupa van Benthem Jutting, van Benthem Jutting (1961: 39).
Opisthostoma crassipupa van Benthem Jutting, Clements (2007: 74).
Opisthostoma crassipupa van Benthem Jutting, Clements et al. (2008: 2760).

#### Type material.

Holotype: ZMA 135994(1) (Seen). Paratype: ZMA 135993(>50) (Seen).

#### Other examined materials.

BOR 5512(>50), BOR 5515(2), BOR 5624(>10), BOR 5629(3), V 8392(>10), V 8407(10), V 8408(4), V 8437(>10), V 8898(7). V 8912(1), V 8956(1), V 9097(9), V 9157(>10), V 9326(>10), V 9353(3), V 9366(10).

Genetic distance between BOR 5512 and BOR 5515 is 14%.

#### Diagnosis.

Shares with *Plectostoma sciaphilum*, *Plectostoma senex*, and *Plectostoma turriforme* the tuba form, but differs by having a slightly or moderately convex apex and depressed apical spire.

#### Description.

**Apex.** Shape: slightly or moderately convex.

**Spire.** Height: 1.3–3.0 mm. Width: 1.2–1.4 mm. Number of whorls: 3 1/2–4 1/2. Apical spire shape: depressed conical. Basal spire shape: ovoid. Whorl periphery: moderately or distinctly convex. Umbilicus: closed by tuba (common) or partially open (rare).

**Constriction.** Parietal teeth: none. Basal teeth: none.

**Tuba.** Coiling direction: type 3 and aperture visible from left lateral view. Tuba whorl length in proportion to spire last whorl: *ca.* 1/2–3/4 Proportion of tuba that attaches to spire: whole.

**Aperture and peristome.** Peristome: double peristomes. Outer peristome shape: same as inner peristome and uniformly projected all around, except the posterior part.

**Spiral lines.** Thick lines: present. Thin lines: present.

**Radial ribs.** Rib density: 6–8 ribs per mm. Rib intensity: thin. Shape: straight. Inclination: moderately prosoclin.

#### Distribution.

Type locality. Gua Musang (4°52'59"N, 101°58'12"E).

Distribution range. This species mainly occurs in the limestome hills that are located between Gua Musang and as far as 30 km radius of Gua Musang. One populations exists at a limestone hill that is located about 90 km north of Gua Musang (Figure 18A).

#### Conservation status.

Least Concern. Almost all the limestone hills are located along main roads and/or surrounded by oil palm plantation or cleared for urban development. However, several large limestone hills that hold the species are located in the well-protected National Park (Taman Negara), State of Pahang, Malaysia.

#### Discussion.

The species is well-characterised, although it displays considerable variability in the shell shape (Appendix 6).

### Plectostoma tonkinianum

(Dautzenberg & Fischer, 1905)

http://species-id.net/wiki/Plectostoma_tonkinianum

**Figures 27.**
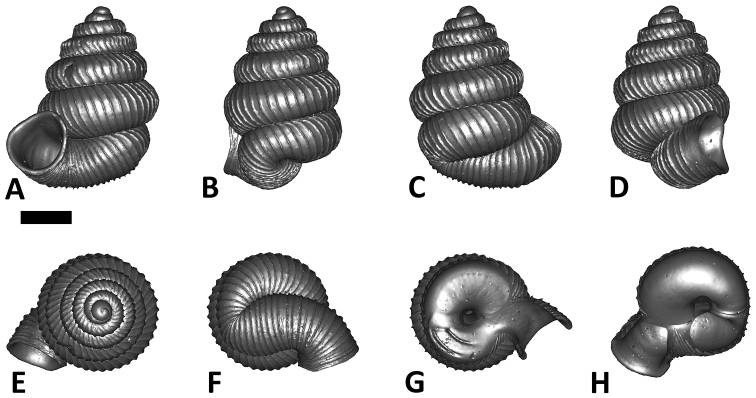
*Plectostoma tonkinianum* (Dautzenberg & Fischer, 1905) V 9940. **A** frontal view **B** left lateral view **C** back view **D** right lateral view **E** top view **F** bottom view; **G** parietal part of constriction inner whorl **H** basal part of constriction inner whorl. Scale bar = 1 mm (for **A–F**).

Figure 27 , Appendix 8

Opisthostoma tonkinianum Dautzenberg & Fischer, 1905: 444, plate 10 - figure 5, 6 and 7 (original description).
Opisthostoma tonkinianum Dautzenberg & Fischer, Saurin (1953: 134).
Opisthostoma tonkinianum Dautzenberg & Fischer, van Benthem Jutting (1962: 12).
Opisthostoma tonkinianum Dautzenberg & Fischer, Fischer (1963: 34).

#### Type material.

Not seen.

#### Other examined materials.

ZMA 162136(1), V 10000(>25), V 10010(>25), V 10022(>25), V 11243(>25), V 11270(>25), V 11432(3), V 11502(>50), V 7937(5), V 9940(>10), V 9957(>25).

#### Diagnosis.

Shares with *Plectostoma whitteni* the general shell form, in terms of apex, spire and tuba, but differs by having two lamella-shaped constriction teeth and a higher spire (> 3 mm).

#### Description.

**Apex.** Shape: distinctly convex.

**Spire.** Height: 3.1–3.8 mm. Width: 2.4–2.6 mm. Number of whorls: 4 3/4–4 7/8. Apical spire shape: depressed conical. Basal spire shape: ovoid. Whorl periphery: distinctly convex. Umbilicus: completely closed by tuba.

**Constriction.** Parietal teeth: two lamellae. Basal teeth: none.

**Tuba.** Coiling direction: type 3 and aperture visible in left lateral view. Tuba whorl length in proportion to spire last whorl: *ca.* 5/8. Proportion of tuba that attaches to spire: whole.

**Aperture and peristome.** Peristome: simple peristome.

**Spiral lines.** Thick lines: absent. Thin lines: present.

**Radial ribs.** Rib density: 6–7 ribs per mm. Rib intensity: thin. Shape: slightly curved to single-humped. Inclination: orthoclin.

#### Distribution.

Type locality. “Tonkin” (Dautzenberg and Fischer 1905). The exact locality should be the limestone hill near Chau Dao.

Distribution range. Limestone hills in the Province of Kien Giang (Figure 18A).

#### Conservation status.

Data deficient. Some recent dead shells have been collected but no information is available on the habitat and population status.

#### Discussion.

In recent years, this species has been recorded at several limestone hills in the vicinity of Ha Tien, which is a popular tourism site. Although the type specimen cannot be located and the exact type locality cannot be determined, the shell morphology of the recent material fits well with the description in the original publication.

In the original publication, Dautzenberg and Fischer (1905) mentioned that this species was collected by M. Mansuy from Tonkin (French protectorate), which was a large area that included part of Southern China, and the Northern parts of Laos and Vietnam. Dautzenberg and Fischer (1905) further provided a list of locations where most of the snails were collected. One of these was, Chau Doc, which is located about 70 km from Ha Tien. Furthermore, a recent intensive land snail survey in the Northern Provinces of Laos, namely, Hua Phan and Luang Prabang, did not discover any *Plectostoma* species (Muratov and Abdou 2006). Hence, *Plectostoma tonkinianum* probably occurs in the small limestone hill cluster in the coastal area of the Southern part of Vietnam and neighboring Cambodia.

### Plectostoma whitteni sp. n.

http://zoobank.org/37B4DE69-9255-4632-AF7F-8EB75D1D4CD8

http://species-id.net/wiki/Plectostoma_whitteni

**Figures 28.**
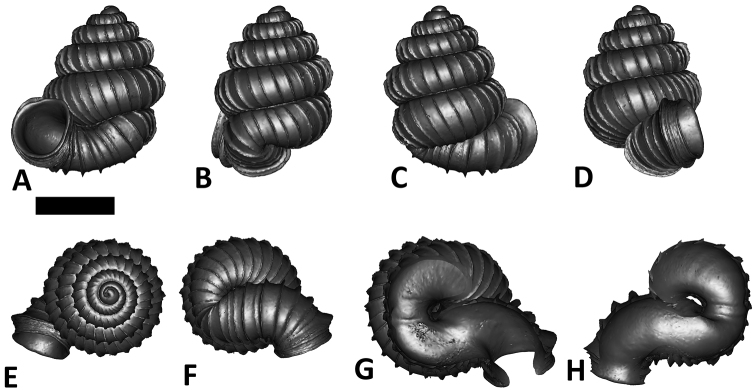
*Plectostoma whitteni* sp. n. BOR 5644. **A** frontal view **B** left lateral view **C** back view **D** right lateral view **E** top view **F** bottom view **G** parietal part of constriction inner whorl **H** basal part of constriction inner whorl. Scale bar = 1 mm (for **A–F**).

Figures 17G , 28 , Appendix 8

#### Type material.

Holotype: BOR 5644(1). Paratypes: BOR 5536(>10), V 8802(1), V 8885(>10).

#### Diagnosis.

Shares with *Plectostoma tonkinianum* the general shell form, in the terms of apex, spire and tuba, but differs by lacking constriction teeth and lower spire (< 2 mm).

#### Etymology.

This species is named after Dr. Tony Whitten, who was senior biodiversity specialist of the World Bank between 1995 and 2010, and is currently the Regional Director for Asia-Pacific Fauna & Flora International. Dr. Whitten has been actively promoting the protection of the biodiversity that is associated with limestone and has been involved in conservation action to protect limestone habitats.

#### Description.

**Apex.** Shape: distinctly convex.

**Spire.** Height: 1.7–1.9 mm. Width: 1.4–1.6 mm. Number of whorls: 3 7/8–4 1/4. Apical spire shape: depressed conical. Basal spire shape: ovoid. Whorl periphery: distinctly convex. Umbilicus: completely closed by tuba.

**Constriction.** Parietal teeth: none. Basal teeth: none.

**Tuba.** Coiling direction: type 3 and aperture visible in left lateral view. Tuba whorl length in proportion to spire last whorl: *ca.* 1/2–5/8. Proportion of tuba that attaches to spire: whole.

**Aperture and peristome.** Peristome: double peristomes. Outer peristome shape: same as inner peristome and uniformly projected all around, except the posterior part.

**Spiral lines.** Thick lines: absent. Thin lines: present.

**Radial ribs.** Rib density: 6–7 ribs per mm. Rib intensity: thin. Shape: straight. Inclination: orthoclin.

#### Distribution.

Type locality. Gua Taat, Tasik Kenyir (4°51'3"N, 102°43'21"E).

Distribution range. This species only occurs in Gua Taat, Tasik Kenyir (Figure 18A).

#### Conservation status.

Least Concern. Gua Taat is located in a prioritised protected National Park with good forest cover.

#### Discussion.

The general shell form of *Plectostoma whitteni* is similar to *Plectostoma tonkinianum*. There is no genetic information for *Plectostoma tonkinianum*, but *Plectostoma whitteni* is genetically closer to *Plectostoma tohchinyawi* than to any of the other 18 species. Nevertheless, *Plectostoma whitteni* is considered a distinct species as compared to *Plectostoma tonkinianum* and *Plectostoma tohchinyawi* because of the lack of constriction teeth.

### Plectostoma sciaphilum

(van Benthem Jutting, 1952)

http://species-id.net/wiki/Plectostoma_sciaphilum

**Figures 29.**
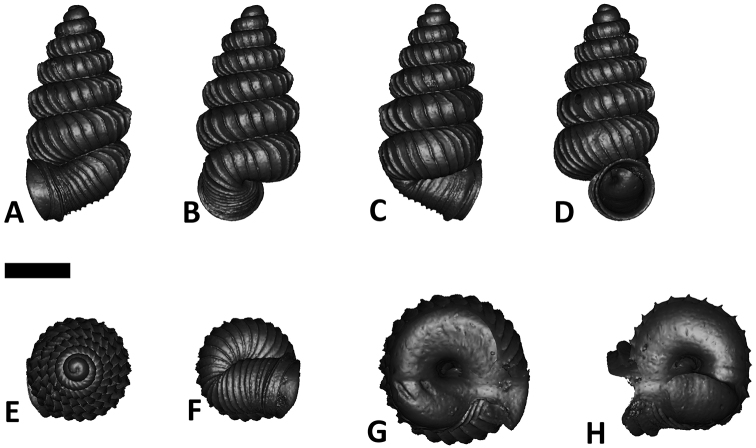
*Plectostoma sciaphilum* (van Benthem Jutting, 1952) ZMA 136050. **A** frontal view **B** left lateral view **C** back view **D** right lateral view **E** top view **F** bottom view **G** parietal part of constriction inner whorl **H** basal part of constriction inner whorl. Scale bar = 1 mm (for **A–F**).

Figure 29 , Appendix 7

Opisthostoma sciaphilum van Benthem Jutting, 1952: 45, figure 23 (original description).

#### Type material.

Holotype: ZMA 136049(1) (Seen). Paratypes: ZMA 136050(>10) (Seen).

#### Diagnosis.

Shares with *Plectostoma senex* and *Plectostoma turriforme* the tuba form, but differs by lacking basal constriction teeth.

#### Description.

**Apex.** Shape: distinctly convex.

**Spire.** Height: 2.6–2.9 mm. Width: 1.5–1.6 mm. Number of whorls: 4 1/4–5 1/2. Apical spire shape: oblong conical. Basal spire shape: ovoid. Whorl periphery: distinctly convex. Umbilicus: completely closed by tuba.

**Constriction.** Parietal teeth: two. Basal teeth: none.

**Tuba.** Coiling direction: type 3 and aperture visible in left lateral view. Tuba whorl length in proportion to spire last whorl: *ca.* 3/8. Proportion of tuba that attaches to spire: whole.

**Aperture and peristome.** Peristome: double peristomes. Outer peristome shape: same as inner peristome and uniformly projected all around, except the posterior part.

**Spiral lines.** Thick lines: present. Thin lines: present.

**Radial ribs.** Rib density: 6–8 ribs per mm. Rib intensity: thin. Shape: slightly curved.

#### Distribution.

Type locality. Bukit Panching, Pahang (3°53'28"N, 103°8'26"E).

Distribution range. Endemic to Bukit Panching (not seen in Figure 18A because its symbol overlaps with *Plectostoma senex*).

#### Conservation status.

Extinct. Its only habitat–Bukit Panching, has been completed quarried away (see also Schilthuizen and Clements 2008). The ruin is now inundated. The status of this species in a previous assessment (IUCN redlist) was: Critically Endangered B2ab(ii,iii) ver. 3.1 (Clements 2009a).

#### Discussion.

See under *Plectostoma turriforme*.

### Plectostoma senex

(van Benthem Jutting, 1952)

http://species-id.net/wiki/Plectostoma_senex

**Figures 30.**
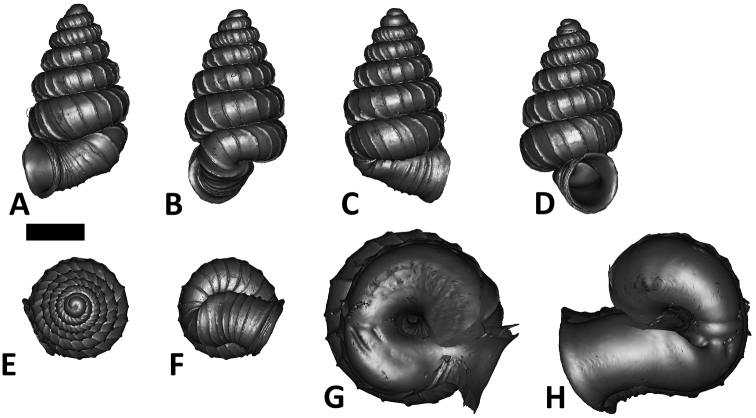
*Plectostoma senex* (van Benthem Jutting, 1952) BOR 5603. **A** frontal view **B** left lateral view **C** back view **D** right lateral view **E** top view **F** bottom view **G** parietal part of constriction inner whorl **H** basal part of constriction inner whorl. Scale bar = 1 mm (for **A–F**).

Figures 17H , 30 , Appendix 7

Opisthostoma senex van Benthem Jutting, 1952: 47, figure 24 (original description).

#### Type material.

Holotype: ZMA 136052(1) (Seen). Paratypes: ZMA 136053(>10) (Seen).

#### Other examined materials.

RMNH 44723(5), BOR 460(1), BOR 5575(3), BOR 5603(2), BOR 5628(>100), BOR 5631(>10), V 5117(9).

#### Diagnosis.

Shares with *Plectostoma sciaphilum* and *Plectostoma turriforme* the tuba form, but differs by having two knob-shaped constriction teeth and fewer than 6 whorls.

#### Description.

**Apex.** Shape: distinctly convex.

**Spire.** Height: 2.6–3.1 mm. Width: 1.6–1.7 mm. Number of whorls: 5 1/8–5 5/8. Apical spire shape: oblong conical. Basal spire shape: ovoid. Whorl periphery: moderately to distinctly convex. Umbilicus: completely closed by tuba.

**Constriction.** Parietal teeth: two. Basal teeth: one transverse tooth.

**Tuba.** Coiling direction: type 3 and aperture visible in left lateral view. Tuba whorl length in proportion to spire last whorl: *ca.* 3/8. Proportion of tuba that attaches to spire: whole.

**Aperture and peristome.** Peristome: double peristomes. Outer peristome shape: same as inner peristome and uniformly projected all around, except the posterior part.

**Spiral lines.** Thick lines: absent. Thin lines: present.

**Radial ribs.** Rib density: 4 ribs per mm. Rib intensity: thin. Shape: slightly curved. Inclination: orthoclin.

#### Distribution.

Type locality. Gua Charas, limestone hill near Sungai Lembing in the state of Pahang, Malaysia (3°54'27"N, 103°8'47"E).

Distribution range. In addition to the type locality, this species had been recorded from Bukit Panching (Figure 18A).

#### Conservation status.

Critically Endangered (B2ab(iii)+C2a(i) ver. 10.1). This species is known from two locations. One of these, Bukit Panching, does not exist anymore. Two intensive surveys at the other location, Bukit Charas, were conducted in Feb. 2010 and May 2011. Two living populations with fewer than 50 individuals were found at wet staglamites near the cave. No other living population was found elsewhere on Bukit Charas. The status of this species in a previous assessment (IUCN redlist) was: vulnerable D2 ver. 3.1 (Clements 2009b).

#### Discussion.

See discussion under *Plectostoma turriforme*.

### Plectostoma turriforme

(van Benthem Jutting, 1952)

http://species-id.net/wiki/Plectostoma_turriforme

**Figures 31.**
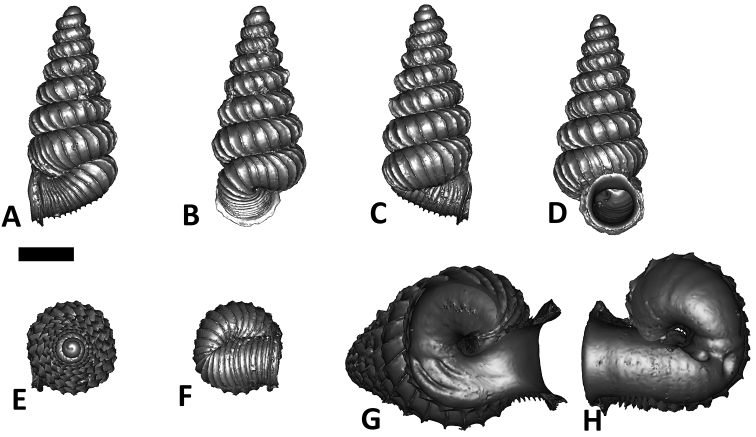
*Plectostoma turriforme* (van Benthem Jutting, 1952) BOR 5609. **A** frontal view **B** leftlateral view **C** back view **D** right lateral view **E** top view **F** bottom view **G** parietal part of constriction inner whorl **H** basal part of constriction inner whorl. Scale bar = 1 mm (for **A–F**).

Figure 31 , Appendix 8

Opisthostoma turriforme van Benthem Jutting, 1952: 43, figure 22 (original description).

#### Type material.

Holotype: ZMA 136068(1) (Seen). Paratypes: ZMA 136069(>10) (Seen).

#### Other examined materials.

BOR 461(2), BOR 5609(2).

#### Diagnosis.

Shares with *Plectostoma sciaphilum* and *Plectostoma senex* the tuba form, but differs by having two lamella-shaped constriction teeth and having more than 6 whorls.

#### Description.

**Apex.** Shape: distinctly convex.

**Spire.** Height: 3.2 mm. Width: 1.6 mm. Number of whorls: 7. Apical spire shape: oblong conical. Basal spire shape: conical. Whorl periphery: distinctly convex. Umbilicus: completely closed by tuba.

**Constriction.** Parietal teeth: two. Basal teeth: two.

**Tuba.** Coiling direction: type 3 and aperture visible in left lateral view. Tuba whorl length in proportion to spire last whorl: *ca.* 3/8. Proportion of tuba that attaches to spire: whole.

**Aperture and peristome.** Peristome: double peristomes. Outer peristome shape: same as inner peristome and uniformly projected all around, except the posterior part.

**Spiral lines.** Thick lines: absent. Thin lines: present.

**Radial ribs.** Rib density: 5 ribs per mm. Rib intensity: thin. Shape: slightly curved. Inclination: orthoclin.

#### Distribution.

Type locality. Bukit Tenggek, Pahang (4°0'51"N, 103°9'34"E).

Distribution range. In addition to the type locality, this species can be found at nearby Bukit Sagu (Figure 18A).

#### Conservation status.

Critically Endangered (B2ab(iii)+C2a(i) ver. 10.1). The whole Bukit Tenggek will disappear by 2014 because of quarrying activity. Moreover, more than half of Bukit Sagu has already been quarried away (see also Schilthuizen and Clements 2008). Although living individuals had been recorded from Bukit Sagu in 1997 (BOR 5609), neither living nor recently dead individuals were found in a recent survey conducted in 2010 and 2011.

#### Discussion.

*Plectostoma turriforme*, *Plectostoma sciaphilum*, and *Plectostoma senex* are three very similar species than occur in the four lenticular easternmost limestone hills in Peninsular Malaysia (Figure 18A). These four hills, namely, Bukit Panching, Bukit Charas, Bukit Sagu, and Bukit Tenggek are located along a 15 km longitudinal transect. *Plectostoma turriforme* occurs at the two former sites and *Plectostoma senex* at the two latter sites. *Plectostoma sciaphilum* occurs sympatrically with *Plectostoma turriforme* in Bukit Panching. These four hills (and thus the three species) are among the most isolated limestone outcrops in Peninsular Malaysia.

### Plectostoma palinhelix

(van Benthem Jutting, 1952)

http://species-id.net/wiki/Plectostoma_palinhelix

**Figures 32.**
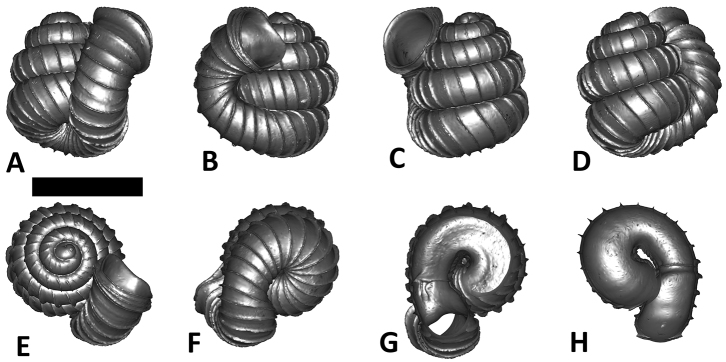
*Plectostoma palinhelix* (van Benthem Jutting, 1952) BOR 5520. **A** frontal view **B** left lateral view **C** back view **D** right lateral view **E** top view **F** bottom view **G** parietal part of constriction inner whorl **H** basal part of constriction inner whorl. Scale bar = 1 mm (for **A–F**).

Figures 17C , 32 , Appendix 12

Opisthostoma palinhelix van Benthem Jutting, 1952: 40, figure 20 (original description).

#### Type material.

Holotype: ZMA 136030(1) (Seen). Paratype: ZMA 136031(>10) (Seen).

#### Other examined materials.

BOR 466(1), BOR 5520(5), V 5104(5).

#### Diagnosis.

Shares with *Plectostoma retrovertens* the spire and tuba form, but differs by having spire height between 1.6–1.9 mm.

#### Description.

**Apex.** Shape: slightly convex.

**Spire.** Height: 1.6–1.9 mm. Width: 1.1–1.3 mm. Number of whorls: 3 3/4–4 1/4. Apical spire shape: depressed conical. Basal spire shape: ovoid. Whorl periphery: moderately to distinctly convex. Umbilicus: partially or completely closed by tuba.

**Constriction.** Parietal teeth: two. Basal teeth: none.

**Tuba.** Coiling direction: type 2 and aperture visible between right lateral and back view; the tuba coils upward until the first teleconch whorl of the spire. Tuba whorl length similar to that of the last whorl of the spire. Proportion of tuba that attaches to spire: whole.

**Aperture and peristome.** Peristome: double peristomes. Outer peristome shape: similer to inner peristome, projected all around, except the posterior part, where the two lateral sides are slightly more projected than the anterior side.

**Spiral lines.** Thick lines: present. Thin lines: present.

**Radial ribs.** Rib density: 7–8 ribs per mm. Rib intensity: thin. Shape: straight. Inclination: orthoclin.

#### Distribution.

Type locality. Bukit Serdam, Pahang (3°49'47"N, 101°55'36"E).

Distribution range. To date, this species has been recorded from three limestone hills, namely Bukit Serdam, Gua Kechil and a small hill nearby (Figure 18C).

#### Conservation status.

Vulnerable (B2ab(iii) ver. 10.1). There are four limestone hills in this area, of which Bukit Serdam and its next unnamed hill support populations (assessment done in 2010 and 2011). Two of the hills, namely, Bukit Serdam and Gunung Panas, are now being quarried. The smallest unnamed hill is highly degraded and Gua Kechil is surrounded by oil palm plantation.

#### Discussion.

See discussion under *Plectostoma retrovertens*.

### Plectostoma retrovertens

(Tomlin, 1938)

http://species-id.net/wiki/Plectostoma_retrovertens

**Figures 33.**
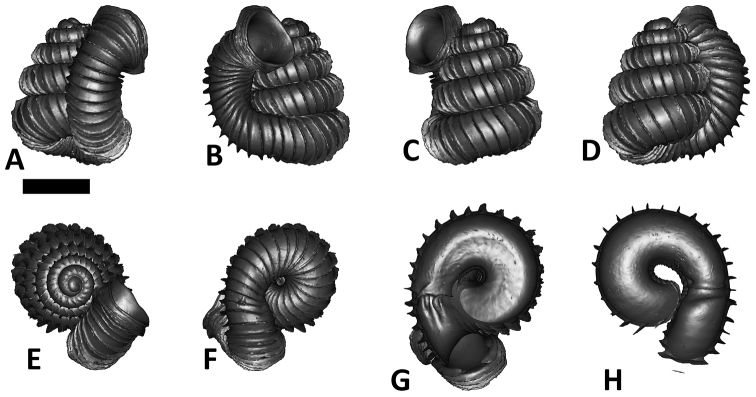
*Plectostoma retrovertens* (Tomlin, 1938) BOR 5559. **A** frontal view **B** left lateral view **C** back view **D** right lateral view **E** top view **F** bottom view **G** parietal part of constriction inner whorl **H** basal part of constriction inner whorl. Scale bar = 1 mm (for **A–F**).

Figures 17R , 33 , Appendix 13

Opisthostoma retrovertens Tomlin, 1938: 73, Plate 2 - figure 3 (original description).
Opisthostoma retrovertens Tomlin, van Benthem Jutting (1952: 39).
Opisthostoma retrovertens Tomlin, Berry (1961).
Opisthostoma retrovertens Tomlin, Berry (1962).
Opisthostoma retrovertens Tomlin, Berry (1963).
Opisthostoma retrovertens Tomlin, Berry (1964).
Opisthostoma retrovertens Tomlin, Berry (1966).
Opisthostoma retrovertens Tomlin, Solem (1966).
Opisthostoma retrovertens Tomlin, Illert (1987: 800, figure 2a).
Opisthostoma retrovertens Tomlin, Solem and Solem (1976: 31).
Opisthostoma retrovertens Tomlin, Heller (2001: 426).
Opisthostoma retrovertens Tomlin, Clements (2007: 74).
Opisthostoma retrovertens Tomlin, Clements et al. (2008: 2760).

#### Type material.

Holotype: BMNH 1938.10.25.2(1) (Seen). Paratype: ZMA 136044(>10) (Seen).

#### Other examined materials.

RMNH 244699(1), RMNH 44725(8), ZMA 162133(>10), ZMA 162148(8), BOR 5559(3), BOR 5621(9), V 5124(9).

#### Diagnosis.

Shares with *Plectostoma palinhelix* the spire and tuba form, but differs by having spire height between 2.3–2.6 mm.

#### Description.

**Apex.** Shape: moderately convex.

**Spire.** Height: 2.3–2.6 mm. Width: 1.5–1.7 mm. Number of whorls: 4 5/8–5. Apical spire shape: depressed conical. Basal spire shape: ovoid. Whorl periphery: distinctly convex. Umbilicus: partially or completely closed by tuba.

**Constriction.** Parietal teeth: two. Basal teeth: none.

**Tuba.** Coiling direction: type 2 and aperture visible between right lateral and back view; the tuba coils upward until the first teleconch whorl of the spire. Tuba whorl length similar to the length of the last whorl of the spire. Proportion of tuba that attaches to spire: whole.

**Aperture and peristome.** Peristome: double peristomes. Outer peristome shape: similer to inner peristome, projected all around, except the posterior part, where two lateral sides are slightly more projected than the anterior side.

**Spiral lines.** Thick lines: present. Thin lines: present.

**Radial ribs.** Rib density: 7–9 ribs per mm. Rib intensity: thin. Shape: straight. Inclination: orthoclin.

#### Distribution.

Type locality. Bukit Chintamanis, Pahang (3°26'45"N, 102°0'51"E).

Distribution range. Endemic to the type locality (Figure 18C).

#### Conservation status.

Critically Endangered (B2ab(iii)+C2a(i) ver. 10.1). This species is endemic to a single limestone hill–Bukit Chintamanis. Most of the western part of this hill is gone due to the quarry activity in the past and this part is now covered by secondary vegetation. The rest of the outcrop is surrounded by plantation, which is subjected to periodic clearing and replanting. According to Berry (1961), live individuals can only be found at the rock surface (Site A in Berry 1961). Berry (1962) reports finding several thousand individuals at Site A.

A recent intensive survey for *Plectostoma retrovertens* was done at the type locality in August 2010 and May 2011. Site A is a rock surface smaller than 20 m^2^. It appears that the vegetation cover and habitat structure of Site A has not changed as compared to the assessment done by Berry (1962, 1964). However, only three live individuals were found during the survey in Aug. 2010. Furthermore, the surrounding of Bukit Chintamanis has been cleared in 2011. Judging by the population trends and habitat conditions together, this species is at the brink of extinction. The status of this species in a previous assessment (IUCN redlist) was: vulnerable D2 ver. 3.1 (Clements 2009c).

#### Discussion.

*Plectostoma retrovertens* and *Plectostoma palinhelix* are very distinct from other *Plectostoma* by having a very long tuba in relation to their spire. In fact, a preliminary phylogenetic analysis suggests that these two are basal species for all Peninsular Malaysia’s *Plectostoma*. These two species are located more 40 km apart on outcrops that belong to the same limestone facies (Figure 18C). The two species are very similar in their shell shape, but *Plectostoma retrovertens* is about one-third larger than *Plectostoma palinhelix*. Furthermore, the genetic divergence between the two species is 10%.

### Plectostoma ikanensis sp. n.

http://zoobank.org/D450E51F-D3A1-49B7-ACA2-E70A5C42ED2C

http://species-id.net/wiki/Plectostoma_ikanensis

**Figures 34.**
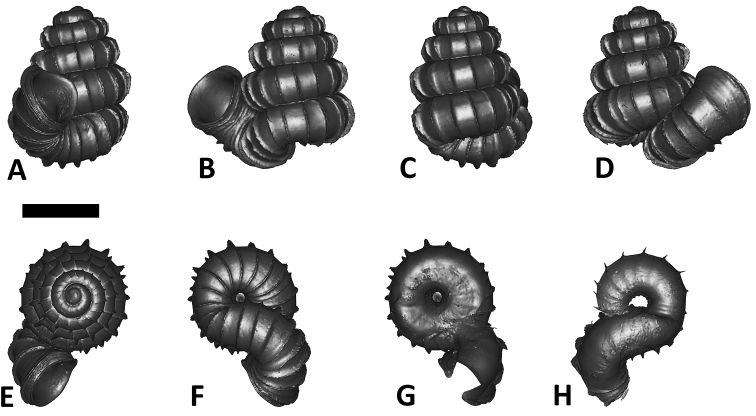
*Plectostoma ikanensis* sp. n. (Form BOR 5504) BOR 5504. **A** frontal view **B** left lateral view **C** back view **D** right lateral view **E** top view **F** bottom view **G** parietal part of constriction inner whorl **H** basal part of constriction inner whorl. Scale bar = 1 mm (for **A–F**).

**Figures 35.**
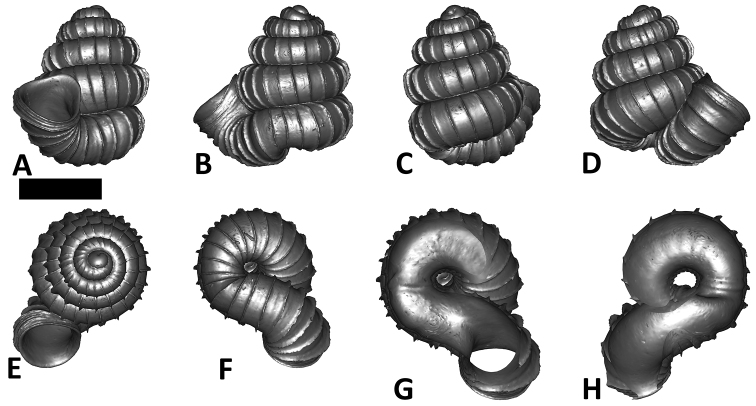
*Plectostoma ikanensis* sp. n. (Form BOR 5507) BOR 5645. **A** frontal view **B** left lateral view **C** back view **D** right lateral view **E** top view **F** bottom view **G** parietal part of constriction inner whorl **H** basal part of constriction inner whorl. Scale bar = 1 mm (for **A–F**).

Figures 17J, K , 34 , 35 , Appendix 11 , 14

#### Type material.

Holotype: BOR 5645(1)

Paratypes: BOR 5507(6), BOR 5622(>50), BOR 5504(>10), V 9446(>100), V 9320(6).

#### Etymology.

This species is named after its type locality–Gua Ikan.

#### Diagnosis.

Shares with *Plectostoma kayiani* the general shell form, in terms of spire and tuba shape, but differs by having both thick and thin spiral lines.

#### Description for shell form 5504

(Figs 17K and 34, and Appendix 14). **Apex.** Shape: moderately convex.

**Spire.** Height: 1.7 mm. Width: 1.3 mm. Number of whorls: 4 1/8–4 1/4. Apical spire shape: depressed conical. Basal spire shape: conical. Whorl periphery: distinctly convex. Umbilicus: open.

**Constriction.** Parietal teeth: none. Basal teeth: none

**Tuba.** Coiling direction: type 2 and aperture visible in right lateral view. Tuba whorl length in proportion to spire last whorl: ca.3/4–7/8. Proportion of tuba that attaches to spire: whole.

**Aperture and peristome.** Peristome: double peristomes. Outer peristome shape: similar to inner peristome, projected all around, except the posterior part, where left lateral sides are slightly more projected than the anterior and right lateral side.

**Spiral lines.** Thick lines: present. Thin lines: present.

**Radial ribs.** Rib density: 5 ribs per mm. Rib intensity: thin. Shape: straight. Inclination: orthoclin.

#### Description for shell form 5507

(Figs 17J and 35, and Appendix 11). **Apex.** Shape: moderately convex.

**Spire.** Height: 1.8–1.9 mm. Width: 1.3 mm. Number of whorls: 3 7/8–4. Apical spire shape: depressed conical. Basal spire shape: conical. Whorl periphery: distinctly convex. Umbilicus: open.

**Constriction.** Parietal teeth: none. Basal teeth: none.

**Tuba.** Coiling direction: type 2 and aperture visible in left lateral view. Tuba whorl length in proportion to spire last whorl: *ca.* 5/8. Proportion of tuba that attaches to spire: whole.

**Aperture and peristome.** Aperture with double peristome. Peristome: double peristomes. Outer peristome shape: similar to inner peristome, projected all around, except the posterior part, where left lateral sides are slightly more projected than the anterior and right lateral side.

**Spiral lines.** Thick lines: present. Thin lines: present.

**Radial ribs.** Rib density: 7 ribs per mm. Rib intensity: thin. Shape: straight. Inclination: orthoclin.

#### Distribution.

Type locality. Gua Ikan in the State of Kelantan (5°21'9"N, 102°1'34"E).

Distribution range. In addition to the type location, this species also occurs at nearby limestone hills as far as 30 km away (Figure 18C).

#### Conservation status.

Least concern. Living populations of *Plectostoma ikanensis* were recorded at the type locality in 2011. The type locality is gazetted as recreation forest.

#### Discussion.

The two forms of *Plectostoma ikanensis* were found in the type locality at two different parts of the hill that within a distance of 100 m. The overall shell appearances of these two forms are very different, especially in terms of tuba coiling direction and spire shape. Interestingly, one of the *Plectostoma ikanensis* forms (i.e. BOR 5507) is very similar to *Plectostoma davisoni* on the basis of these two shell characters. In most of the other *Plectostoma* species examined in this study, tuba coiling direction and spire shape are rather stable characters within a species. Nevertheless, both *Plectostoma ikanensis* forms lack a constriction which unite them and distinguish them from other similar species (see Diagnosis). In addition to the morphological evidence, we found that the genetic divergence of these two forms is smaller than 1% (Table 4), and our preliminary phylogenetic analysis shows that the two forms are reciprocally monophyletic. We therefore conclude that they be classified as the same species.

### Plectostoma kayiani sp. n.

http://zoobank.org/30F310AB-7709-4FF3-B5DD-5477AEBAA8BD

http://species-id.net/wiki/Plectostoma_kayiani

**Figures 36.**
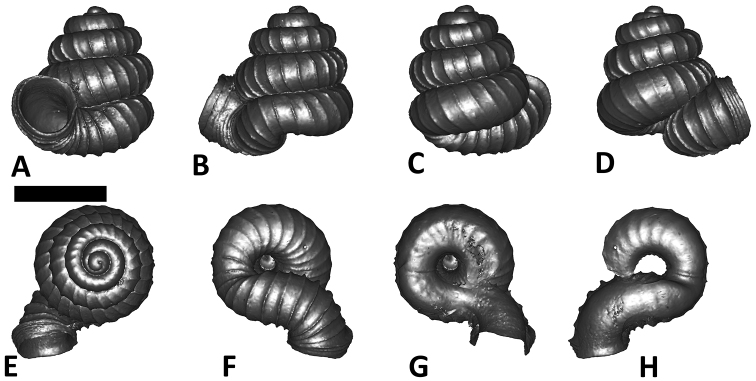
*Plectostoma kayiani* sp. n. RMNH 330803. **A** frontal view **B** left lateral view **C** back view **D** right lateral view **E** top view **F** bottom view **G** parietal part of constriction inner whorl **H** basal part of constriction inner whorl. Scale bar = 1 mm (for **A–F**).

Figure 36 , Appendix 11

#### Type material.

Holotype: RMNH 330803 (1). Paratypes: V 8883(6), V 14243(1).

#### Etymology.

This species is named after Kay Arnold and Ian Mellsop from New Zealand, who have generously supported wildlife conservation work in many parts of Peninsular Malaysia, including the forests around Lake Kenyir where this species was discovered.

#### Diagnosis.

Shares with *Plectostoma ikanensis* the general shell form, in terms of spire and tuba shape, but differs by lacking thick spiral lines.

#### Description.

**Apex.** Shape: slightly to moderately convex.

**Spire.** Height: 1.4–1.6 mm. Width: 1.2–1.4 mm. Number of whorls: 3 1/2–3 3/4. Apical spire shape: depressed conical. Basal spire shape: conical. Whorl periphery: distinctly convex. Umbilicus: open.

**Constriction.** Parietal teeth: none. Basal teeth: none.

**Tuba.** Coiling direction: type 2 and aperture visible from front view. Tuba whorl length in proportion to spire last whorl: *ca.* 5/8–3/4. Proportion of tuba that attaches to spire: almost whole except the part near the aperture.

**Aperture and peristome.** Peristome: double peristomes. Outer peristome shape: similer to inner peristome, projected all around, except the posterior part, where left lateral sides are slightly more projected than the anterior and right lateral side.

**Spiral lines.** Thick lines: absent. Thin lines: present.

**Radial ribs.** Rib density: 8–10 ribs per mm. Rib intensity: thin. Shape: slightly curved. Inclination: orthoclin.

#### Distribution.

Type locality. Gua Bewah, Tasik Kenyir (4°51'3"N, 102°43'21"E).

Distribution range. To date, this species is only known to occur at two neighbouring limestone outcrops, namely, Gua Bewah and Gua Taat at the southern part of Tasik Kenyir (Figure 18C).

#### Conservation status.

Least concern. The outcrops where this species is found, are partially submerged in Southeast-Asia’s largest man-made lake, Tasik Kenyir. Despite this, there is a good forest cover around and on the limestone outcrops. Furthermore, these two hills are located in a protected National Park.

#### Discussion.

This species occurs sympatrically with *Plectostoma tohchinyawi* and *Plectostoma whitteni*.

### Plectostoma davisoni sp. n.

http://zoobank.org/D54FF7DC-7DD6-44D3-8A53-C45DEAF5243E

http://species-id.net/wiki/Plectostoma_davisoni

**Figures 37.**
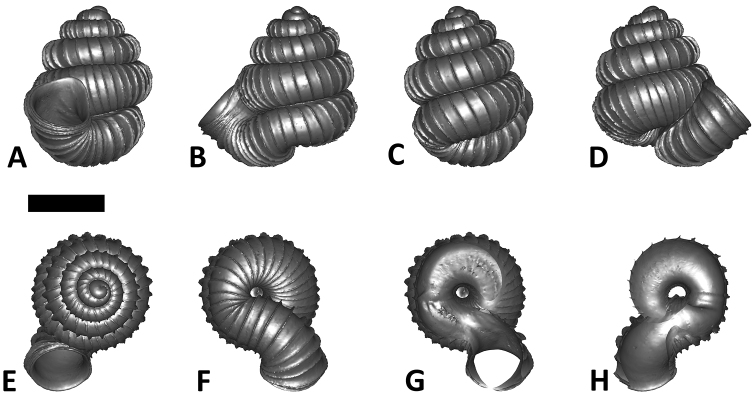
*Plectostoma davisoni* sp. n. BOR 5646. **A** frontal view **B** left lateral view **C** back view **D** right lateral view **E** top view **F** bottom view **G** parietal part of constriction inner whorl **H** basal part of constriction inner whorl. Scale bar = 1 mm (for **A–F**).

Figure 37 , Appendix 9 , 10

#### Type material.

Holotype: BOR 5646(1)

Paratypes: ZMA 162069(>25), ZMA 162070(3), ZMA 162071(>50), ZMA 162146(7), ZMA 162147(8), BOR 5508(>25), BOR 5626(>25), BOR 5641(9), V 8652(6), V 8929(>25), V 9206(6), V 8265(>10), V 8301(>25), V 9243(>50), V 9340(7), V 9417(7), V 14242(5).

#### Diagnosis.

Shares with *Plectostoma relauensis* the general shell form, in terms of apex, spire, and tuba shape, but differs by having two parietal constriction teeth.

#### Etymology.

This species is named after Dr. Geoffrey Davison, who has been involved in the conservation of limestone hills in Malaysia, and has collected a lot of snail specimens, many of which are included in this revision of *Plectostoma* from Malaysia.

#### Description.

**Apex.** Shape: slightly to moderately convex.

**Spire.** Height: 1.6–2.0 mm. Width: 1.4–1.5 mm. Number of whorls: 3 5/8–4 7/8. Apical spire shape: depressed conical. Basal spire shape: ovoid. Whorl periphery: moderately to distinctly convex. Umbilicus: open.

**Constriction.** Parietal teeth: two. Basal teeth: none.

**Tuba.** Coiling direction: type 2 and aperture visible in frontal view. Tuba whorl length in proportion to spire last whorl: ca. 5/8–3/4. Proportion of tuba that attaches to spire: whole.

**Aperture and peristome.** Peristome: double peristomes. Outer peristome shape: similer to inner peristome, projected all around, except the posterior part, where the two lateral sides are slightly more projected than the anterior side.

**Spiral lines.** Thick lines: present. Thin lines: present.

**Radial ribs.** Rib density: 6–7 ribs per mm. Rib intensity: thin. Shape: straight. Inclination: orthoclin.

#### Distribution.

Type locality. Limestone hill on the right hand side of the road D29, at km 17 when travelling from Jelawang to Gua Musang (4°59'4"N, 101°57'53"E).

Distribution range. This species has a very large distribution range, *ca.* 80 km diameter. It can be found in many limestone outcrops in the central part of Peninsular Malaysia, mainly in the State of Kelantan (Figure 17D). In addition, it can also be found in a cluster of limestone hills located at upper Sungai Keniyam Kecil in Taman Negara (ca. 60 km from Gua Musang).

#### Conservation status.

Least concern. This is a widespread species. Although many hills in Kelantan are being degraded and surrounded by oil palm plantations, there are a few well protected hills in Taman Negara in Pahang, where this species occurs.

#### Discussion.

This species is highly variable in the shell form (Appendix 9 and Appendix 10), and has a very wide distribution range which partly overlaps with many other *Plectostoma* species (Figure 18). It is very densely distributed in the State of Kelantan, parapatric with *Plectostoma christae*. The species becomes more sparse toward the limestone hills in Taman Negara, Pahang (Figure 18C). In view of this, it is possible that the species actually consists of two or more cryptic species, and thus more genetic data are needed.

### Plectostoma relauensis sp. n.

http://zoobank.org/7BC76202-2661-4F7C-A922-4DBA32622400

http://species-id.net/wiki/Plectostoma_relauensis

**Figures 38.**
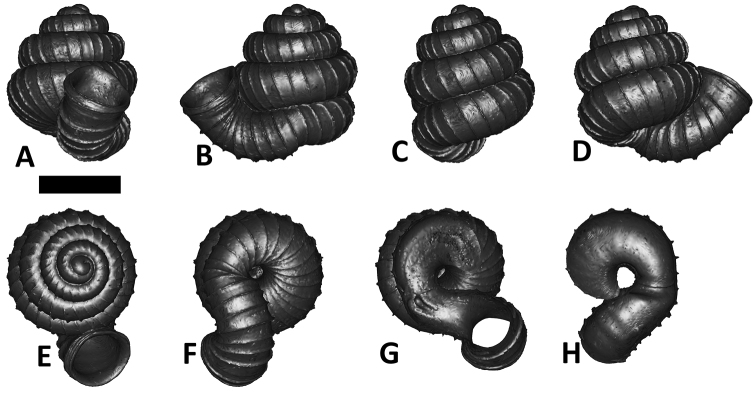
*Plectostoma relauensis* sp. n. BOR 5511. **A** frontal view **B** left lateral view **C** back view **D** right lateral view **E** top view **F** bottom view **G** parietal part of constriction inner whorl **H** basal part of constriction inner whorl. Scale bar = 1 mm (for **A–F**).

Figures 17P , 38 , Appendix 13

#### Type material.

Holotype: BOR 5647(1). Paratypes: BOR 463(2), BOR 5511(>25), V 8169(9).

#### Etymology.

This species is named after its type locality–Relau substation of Taman Negara, where Gua Gajah is located.

#### Diagnosis.

Shares with *Plectostoma davisoni* the general shell form, in terms of apex, spire, and tuba shape, but differs by having a single parietal constriction tooth.

#### Description.

**Apex.** Shape: slightly to moderately convex.

**Spire.** Height: 1.5–1.9 mm. Width: 1.5–1.6 mm. Number of whorls: 3 3/8–3 5/8. Apical spire shape: depressed conical. Basal spire shape: ovoid. Whorl periphery: moderately to distinctly convex. Umbilicus: open.

**Constriction.** Parietal teeth: one. Basal teeth: none.

**Tuba.** Coiling direction: type 2 and aperture visible in frontal view. Tuba whorl length in proportion to spire last whorl: ca. 1/2–5/8. Proportion of tuba that attaches to spire: whole.

**Aperture and peristome.** Peristome: double peristomes. Outer peristome shape: same as inner peristome and uniformly projected all around, except the posterior part.

**Spiral lines.** Thick lines: present. Thin lines: present.

**Radial ribs.** Rib density: 4–7 ribs per mm. Rib intensity: thin. Shape: straight to slightly curved. Inclination: orthoclin.

#### Distribution.

Type locality. Gua Gajah in the Relau substation, Taman Negara. (4°38'15"N, 102°3'50"E).

Distribution range. This species also occurs at a limestone hill located about 20 km north of the type locality. BOR 463 was collection in Pulau Singa Besar, which is located about 300 km from type locality. The reliability of the collection data is dubious (see also BOR 462 of *Plectostoma sinyumensis*).

#### Conservation status.

Least Concern. A large living population of *Plectostoma relauensis* was found at Gua Gajah, which is located in the National Park.

#### Discussion.

This species occurs sympatrically with *Plectostoma siphonostomum* on the same limestone hills. Despite the high density of limestone hills in the area, where many other *Plectostoma* species occur, *Plectostoma relauensis* is only found in two of these (Figure 18).

### Plectostoma kakiense

(Tomlin, 1948)

http://species-id.net/wiki/Plectostoma_kakiense

**Figures 39.**
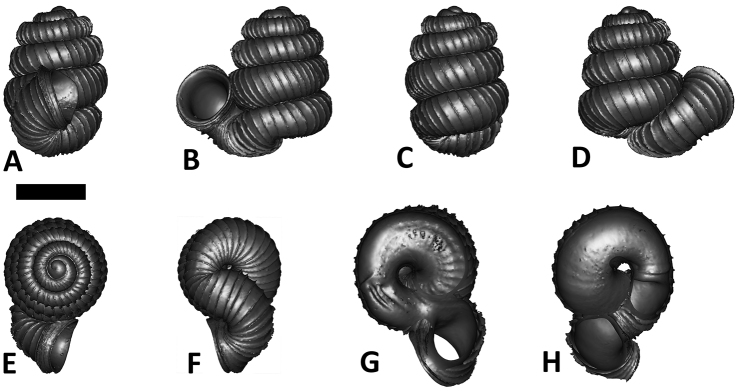
*Plectostoma kakiense* (Tomlin, 1948) BOR 5516. **A** frontal view **B** left lateral view **C** back view **D** right lateral view **E** top view **F** bottom view **G** parietal part of constriction inner whorl **H** basal part of constriction inner whorl. Scale bar = 1 mm (for **A–F**).

Figures 17D , 39 , Appendix 11

Opisthostoma kakiense Tomlin, 1948: 225, Plate 2 - figure 5 (original description).
Opisthostoma kakiense Tomlin, van Benthem Jutting (1952: 39).
Opisthostoma kakiense Tomlin, Ali and Yaakob (2001: 145).
Opisthostoma sp., Razalli et al. (2010: figure 2C).

#### Type material.

Holotype: BMNH 1948.10.2.3(1) (seen).

#### Other examined materials.

ZMA 136009(1), ZMA 162094(5), BOR 445(1), BOR 5516(>25), BOR 5517(>10), BOR 5598(3), V 8789(2).

#### Diagnosis.

Shares with *Plectostoma kubuensis* the general shell form, in terms of apex, spire, and tuba shape, but differs by having two parietal constriction teeth.

#### Description.

**Apex.** Shape: slightly to moderately convex.

**Spire.** Height: 1.7–2.1 mm. Width: 1.5–1.6 mm. Number of whorls: 3 3/4–4 1/4. Apical spire shape: depressed conical. Basal spire shape: ovoid. Whorl periphery: moderately to distinctly convex. Umbilicus: partially closed by tuba.

**Constriction.** Parietal teeth: two. Basal teeth: none.

**Tuba.** Coiling direction: type 2 and aperture visible in right lateral view. Tuba whorl length in proportion to spire last whorl: ca. 5/8–3/4. Proportion of tuba that attaches to spire: whole.

**Aperture and peristome.** Peristome: double peristomes. Outer peristome shape: similar to inner peristome, projected all around, except the posterior part, where the two lateral sides are slightly more projected than the anterior side.

**Spiral lines.** Thick lines: absent. Thin lines: present.

**Radial ribs.** Rib density: 8–10 ribs per mm. Rib intensity: thin. Shape: straight to slightly curved. Inclination: moderately prosoclin.

#### Distribution.

Type locality. Kaki Bukit (6°38'42"N, 100°12'6"E) (Figure 18C). Distribution range. Wang Kelian limestone outcrops.

#### Conservation status.

Near threatened. To date, only two populations are known for two large limestone outcrops in the vast limestone formation in Perlis. These two locations are Kaki Bukit and Wang Kelian, both located in the protected Wang Kelian State Park.

Although Kaki Bukit is a large limestone outcrop with good forest cover, the population density of *Plectostoma kakiense* is very low. During a survey in May 2011, this species was only found near the top of Kaki Bukit where several hundred individuals were found at one location (limestone wall) within an area of ca. 10 m^2^. There were several small populations (fewer than 10 individuals) in small pockets of suitable habitat. The population in Wang Kelian was recorded by Ali and Yaakob (2001), but its status is not known. No live or dead individuals were collected at the dozen isolated limestone hills located within 5 km around Wang Kelian State Park.

#### Discussion.

See discussion under *Plectostoma kubuensis*.

### Plectostoma kubuensis sp. n.

http://zoobank.org/7316ED06-4AF5-4E23-89E3-0BCE01E8B1C5

http://species-id.net/wiki/Plectostoma_kubuensis

**Figures 40.**
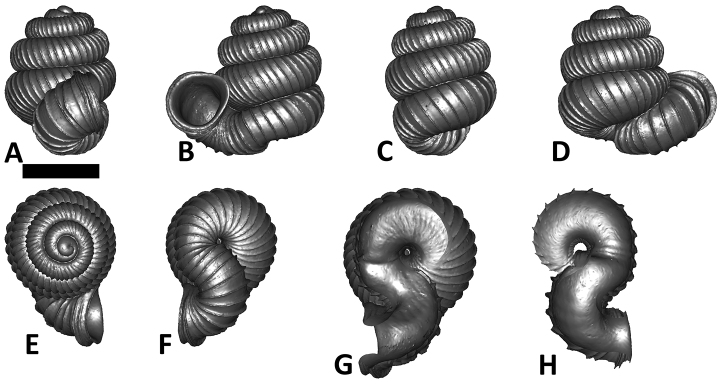
*Plectostoma kubuensis* sp. n. BOR 5648. **A** frontal view **B** left lateral view **C** back view **D** right lateral view **E** top view **F** bottom view **G** parietal part of constriction inner whorl **H** basal part of constriction inner whorl. Scale bar = 1 mm (for **A–F**).

Figures 17F , 40 , Appendix 12

#### Type material.

Holotype: BOR 5648(1). Paratypes: BOR 5518(>25), BOR 5519(>10).

#### Diagnosis.

Shares with *Plectostoma kakiense* the general shell form, in terms of apex, spire, and tuba shape, but differs by lacking constriction teeth.

#### Etymology.

This species is named after its type locality–Bukit Kubu, Perlis.

#### Description.

**Apex.** Shape: slightly to moderately convex.

**Spire.** Height: 1.6–2.0 mm. Width: 1.3–1.5 mm. Number of whorls: 3 5/8–4 1/4. Apical spire shape: depressed conical. Basal spire shape: ovoid. Whorl periphery: moderately to distinctly convex. Umbilicus: open or partially closed by tuba.

**Constriction.** Parietal teeth: none. Basal teeth: none.

**Tuba.** Coiling direction: type 3 and aperture visible in right lateral view. Tuba whorl length in proportion to spire last whorl: *ca.* 5/8–3/4. Proportion of tuba that attaches to spire: whole.

**Aperture and peristome.** Peristome: double peristomes. Outer peristome shape: similar to inner peristome, projected all around, except the posterior part, where the left lateral sides are slightly more projected than the anterior and right lateral side.

**Spiral lines.** Thick lines: absent. Thin lines: present.

**Radial ribs.** Rib density: 10–11 ribs per mm. Rib intensity: thin. Shape: straight to slightly curved. Inclination: moderately prosoclin.

#### Distribution.

Type locality. Bukit Kubu (6°24'15"N, 100°8'37"E) (Figure 18C).

Distribution range. Endemic to the type locality.

#### Conservation status.

Endangered (B2ab(iii)+C2a(i) ver. 10.1). During a survey of a dozen isolated limestone hills of the State of Perlis in May 2011, only Bukit Kubu was found to support a living population of *Plectostoma kubuensis*, consisting of several hundred individuals at the top of Bukit Kubu. Several very small populations (< 50 individuals) live at the other part of Bukit Kubu where the habitat is relatively more exposed and dry. Bukit Kubu is gazetted by Perlis State government as a recreation forest, and its surroundings consist of urban development and paddy fields.

#### Discussion.

From the conchological point of view, the overall shape and size of *Plectostoma kubuensis* and *Plectostoma kakiense* is almost the same. Nevertheless, two inconspicuous but significant shell characters, namely, basal and parietal constriction teeth, mark the difference between these two species. Despite the similarity in shell form, there is a great genetic distance between the two species (16%). The species occur at two limestone hills that lie about 30 km apart. During our survey, we could not find either species at the six limestone hills that are located between these two hills. A single *Plectostoma* shell was recorded by Norhanis et al. (2010) from Pulau Dayang Bunting, Langkawi, and might belong to this species.

### Plectostoma laemodes

(van Benthem Jutting, 1961)

http://species-id.net/wiki/Plectostoma_laemodes

**Figures 41.**
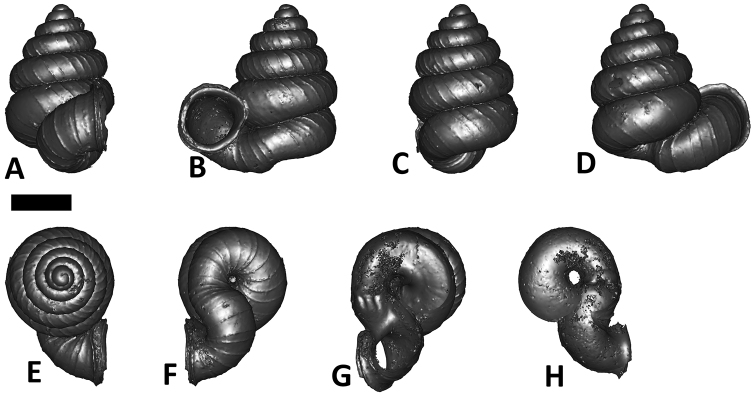
*Plectostoma laemodes* (van Benthem Jutting, 1961) ZMA 136011. **A** frontal view **B** left lateral view **C** back view **D** right lateral view **E** top view **F** bottom view **G** parietal part of constriction inner whorl **H** basal part of constriction inner whorl. Scale bar = 1 mm (for **A–F**).

Figure 41 , Appendix 12

Opisthostoma laemodes van Benthem Jutting, 1961: 40, plate 11 - figure 6 (original description).

#### Type material.

Holotype: ZMA 136010(1) (Seen). Paratypes: ZMA 136011(3) (Seen), ZMA 136012(3) (Seen), ZMA 136013(4) (Seen).

#### Other examined materials.

RMNH 156267(7), V 8490(>25).

#### Diagnosis.

Shares with *Plectostoma salpidomon* the general shell form, in terms of apex, spire, and tuba shape, but differs by having the whole tuba attached to the spire.

#### Description.

**Apex.** Shape: distinctly convex.

**Spire.** Height: 2.3–2.7 mm. Width: 1.6–1.8 mm. Number of whorls: 3 1/8–4 5/8. Apical spire shape: depressed conical. Basal spire shape: ovoid. Whorl periphery: moderately to distinctly convex. Umbilicus: open or partially closed by tuba.

**Constriction.** Parietal teeth: two. Basal teeth: none.

**Tuba.** Coiling direction: type 3 and aperture visible in right lateral view. Tuba whorl length in proportion to spire last whorl: ca. 1/2–5/8. Proportion of tuba that attaches to spire: whole.

**Aperture and peristome.** Peristome: double peristomes. Outer peristome shape: similar to inner peristome, projected all around, except the posterior part, where the left lateral sides are slightly more projected than the anterior and right lateral side.

**Spiral lines.** Thick lines: absent. Thin lines: present.

**Radial ribs.** Rib density: 6–7 ribs per mm. Rib intensity: thin. Shape: straight to slightly curved. Inclination: prosoclin.

#### Distribution.

Type locality. Batu Tai Gadjah, Ulu Keniyam Kechil, Taman Negara, Pahang Malaysia (4°37'0"N, 102°25'14"E) (Figure 18B).

Distribution range. This species mainly occurs in a cluster of limestone hills that are located at upper Sungai Keniyam Kecil in Taman Negara (Figure 18B). In addition, this species has been recorded from Bukit Jereng, Blau, Kelantan, which is about 80 km west from Sungai Keniyam Kecil.

#### Conservation status.

Least concern. Although no living population has been recorded so far, several large and well protected limestone hills in the Taman Negara probably support a viable population.

#### Discussion.

From a conchological point of view, this species is similar to *Plectostoma salpidomon*; the two may be closely related. In view of biogeography, this species occurs parapatrically with *Plectostoma salpidomon*, which occurs in the limestone hills in between Bukit Jereng and the limestone cluster at Sungai Keniyam Kecil (Figure 18B). Further genetic data are needed to verify the taxonomic status of *Plectostoma laemodes*.

### Plectostoma salpidomon

(van Benthem Jutting, 1952)

http://species-id.net/wiki/Plectostoma_salpidomon

**Figures 42.**
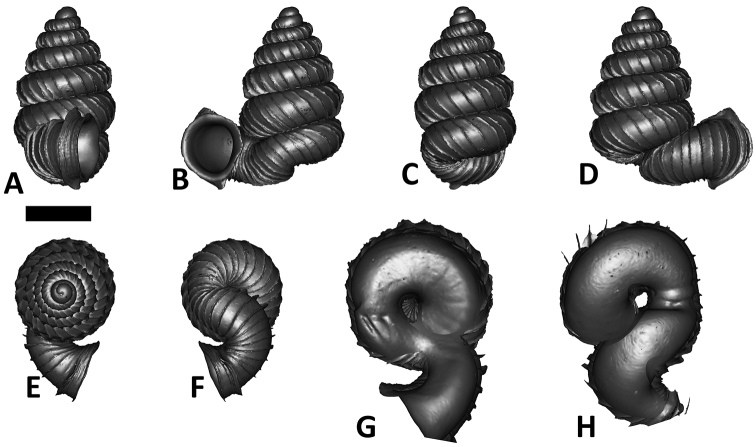
*Plectostoma salpidomon* (van Benthem Jutting, 1952) BOR 5539. **A** frontal view **B** leftlateral view **C** back view **D** right lateral view **E** top view **F** bottom view **G** parietal part of constriction inner whorl **H** basal part of constriction inner whorl. Scale bar = 1 mm (for **A–F**).

Figures 17A , 42 , Appendix 13

Opisthostoma salpidomon van Benthem Jutting, 1952: 42, figure 21 (original description).
Opisthostoma salpidomon van Benthem Jutting, van Benthem Jutting (1961: 39).

#### Type material.

Holotype: ZMA 136045(1) (Seen). Paratypes: ZMA 136046(>25) (Seen), ZMA 136047(2) (Seen), ZMA 136048(>10) (Seen).

#### Other examined materials.

ZMA 162137(>25), BOR 459(1), BOR 5539(>10), BOR 5540(>10), BOR 5541(5), BOR 5542(>25), BOR 5569(>25), BOR 5611(1), V 8171(4), V 8658(>25), V 8706(6), V 9116(7), V 9292(3).

#### Diagnosis.

Shares with *Plectostoma laemodes* the general shell form, in terms of apex, spire, and tuba shape, but differs by having a tuba that attaches partly to the spire.

#### Description.

**Apex.** Shape: distinctly convex.

**Spire.** Height: 2.5–2.8 mm. Width: 1.5–1.7 mm. Number of whorls: 4 5/8–5 1/4. Apical spire shape: depressed conical. Basal spire shape: ovoid. Whorl periphery: moderately to distinctly convex. Umbilicus: completely or partially closed by tuba.

**Constriction.** Parietal teeth: two. Basal teeth: none.

**Tuba.** Coiling direction: type 2 and aperture visible in right lateral view. Tuba whorl length in proportion to spire last whorl: ca. 3/4–7/8. Proportion of tuba that attaches to spire: less than 2/3.

**Aperture and peristome.** Peristome: double peristomes. Outer peristome shape: similar to inner peristome, projected all around, except the posterior part, where the two lateral sides are slightly more projected than the anterior side.

**Spiral lines.** Thick lines: absent. Thin lines: present.

**Radial ribs.** Rib density: 5–6 ribs per mm. Rib intensity: thin. Shape: slightly curved. Inclination: prosoclin.

#### Distribution.

Type locality. Gua Bama, Pahang (4°11'37"N, 101°58'2"E) (Figure 18B).

Distribution range. *Plectostoma salpidomon* can be found in many limestone hills in the valley in between the Titiwangsa, Tahan and Benom mountain ranges (Figure 18B).

#### Conservation status.

Least concern. *Plectostoma salpidomon* has a wide distribution range. Living populations have been recorded in many limestone hills and some of these are in protected areas.

#### Discussion.

See discussion under *Plectostoma laemodes*.

### Plectostoma kitteli

(Maassen, 2002)

http://species-id.net/wiki/Plectostoma_kitteli

**Figures 43.**
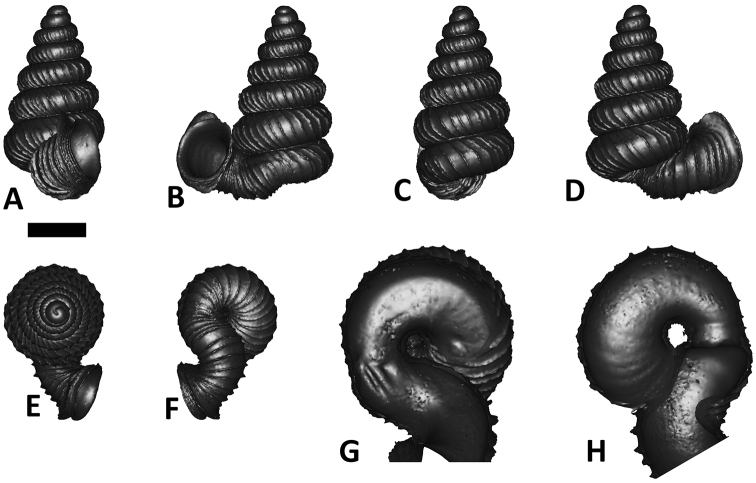
*Plectostoma kitteli* (Maassen, 2002) V 12697. **A** frontal view **B** left lateral view **C** back view **D** right lateral view **E** top view **F** bottom view **G** parietal part of constriction inner whorl **H** basal part of constriction inner whorl. Scale bar = 1 mm (for **A–F**).

Figure 43 , Appendix 11

Opisthostoma kitteli Maassen, 2002: 176, figures 35 & 36 (original description).

#### Type material.

Holotype: RMNH 92942(1) (seen). Paratypes: RMNH 92956(>25) (Seen), V 12697(9) (Seen).

#### Other examined materials.

BOR 1697(>10). YSC.

#### Diagnosis.

Shares with *Plectostoma charasense*, *Plectostoma tohchinyawi*, and *Plectostoma klongsangensis* the general shell form, in terms of apex, spire, and tuba shape, but differs by lacking thick spiral lines and basal constriction teeth.

#### Description.

**Apex.** Shape: distinctly convex.

**Spire.** Height: 2.9–3.0 mm. Width: 1.7–1.8 mm. Number of whorls: 5 5/8–5 3/4. Apical spire shape: oblong conical. Basal spire shape: conical. Whorl periphery: distinctly convex. Umbilicus: partially closed by tuba.

**Constriction.** Parietal teeth: two. Basal teeth: none.

**Tuba.** Coiling direction: type 2 and aperture visible in right lateral view. Tuba whorl length in proportion to spire last whorl: ca. 3/4–7/8. Proportion of tuba that attaches to spire: less than 2/3.

**Aperture and peristome.** Peristome: double peristomes. Outer peristome shape: similar to inner peristome, projected all around, except the posterior part, where the two lateral sides are slightly more projected than the anterior side.

**Spiral lines.** Thick lines: absent. Thin lines: present.

**Radial ribs.** Rib density: 5–6 ribs per mm. Rib intensity: thin. Shape: slightly curved. Inclination: orthoclin.

#### Distribution.

Type locality. Cave Pangian (local name Ngalau Pangian - 0°27'45"S, 100°45'8"E)

Distribution range. This species was also found at a limestone hill near Kampung Desa Gadut, West Sumatra (0°15'36"S, 100°44'16"E). This hill is located about 20 km from the type locality. In addition, a private collector (Yansen Chen, pers. comm.) also collected the same species at Tiangko cave, about 230 km from the type locality (Figure 18B). It therefore appears to have a wide distribution range in Sumatra. It may thus be expected that the limestone outcrops around the three hills mentioned, might support the same species.

#### Conservation status.

Data deficient. This species has only been recorded from three limestone hills in West Sumatra. The population status and actual distribution range of this species remain unknown. Nevertheless, concern is warrented, as the surroundings of the type locality and several other limestone outcrops in the same area are highly degraded.

#### Discussion.

There are large areas in the Northern and Western provinces of Sumatra that are covered by limestone hills (Verstappen and Genootschap 1973). Despite intensive malacological surveys in some of these areas (van Benthem Jutting 1959), no *Plectostoma* species was found until 1997. To date, *Plectostoma kitteli* is the only *Plectostoma* species found in Sumatra (Maassen 2002).

### Plectostoma klongsangensis

(Panha, 1997)

http://species-id.net/wiki/Plectostoma_klongsangensis

Opisthostoma klongsangensis Panha, 1997: 133, figure 1 (original description).
Opisthostoma klongsangensis Panha, Hemmen and Hemmen (2001: 39).
Opisthostoma klongsangensis Panha, Nabhitabhata (2009: 50).

#### Type material.

Holotype: CUIZMD 0001 (not seen). Paratypes: CUIZMDM 0002 (Not seen), CUIZMDM 0003 in ZMA (Not seen, specimen could not be located in ZMA).

#### Diagnosis.

Shares with *Plectostoma charasense*, *Plectostoma tohchinyawi*, and *Plectostoma kitteli* the general shell form, in terms of apex, spire, and tuba, but differs by having both thin and thick spiral lines, and the left lateral side of outer peristome projected more than three times the distance of the right lateral side of outer peristome.

**Description** (estimated from figure in Panha 1996). **Apex.** Shape: distinctly convex.

**Spire.** Height: 2.3 mm. Width: 1.7 mm. Number of whorls: 5 1/2. Apical spire shape: oblong conical. Basal spire shape: conical. Whorl periphery: distinctly convex. Umbilicus: open.

**Constriction.** Unknown.

**Tuba.** Coiling direction: type 2 and aperture visible in right lateral view. Tuba whorl length in proportion to spire last whorl: ca. 7/8. Proportion of tuba that attaches to spire: less than 2/3.

**Aperture and peristome.** Peristome: double peristomes. Outer peristome shape: different from inner peristome, the left lateral side of outer peristome is projected from inner peristome about 0.7 mm, and the right side of outer peristome about 0.1 mm, but narrowed toward the anterior part of outer peristome.

**Spiral lines.** Thick lines: present. Thin lines: present.

**Radial ribs.** Rib density: 3 ribs per mm. Rib intensity: thick. Shape: single-looped. Inclination: prosoclin.

#### Distribution.

Type locality. Khlong Saeng Wildlife Sanctuary Surat Thani Province, Thailand (8°31'13"N, 98°25'17"E) (Figure 18B).

Distribution range. Unknown.

#### Conservation status.

Data deficient. This species has been collected once in Khlong Saeng Wildlife Sanctuary.

#### Discussion.

From a conchological point of view, this species appears not to be related to *Plectostoma* species from the Malay Peninsula and Sumatra. Instead, it is almost identical to *Plectostoma mirabile*, an endemic species from Gomantong Cave, Sabah, Borneo. These two species are separated by the South China Sea and located more than 2000 km apart. It remains to be determined whether this similarity is due to a disjunct distribution of closely related forms, or rather convergent shell evolution.

The taxonomic status of the species remains doubtful. In the original publication, Panha (1996) compared it with *Plectostoma heteropleuron* (Vermeulen 1994) and *Plectostoma perspectivum* (Vermeulen, 1994) from Northern Borneo, but not with *Plectostoma mirabile* (Smith, 1893), which has an almost identical shell as *Plectostoma klongsangensis*. This is all the more remarkable, as *Plectostoma mirabile* (Smith, 1893) was treated in Vermeulen (1994), where both *Plectostoma heteropleuron* and *Plectostoma perspectivum* were originally described.

In conclusion, more data are needed to verify the taxonomic status and the interesting biogeography of this species.

### Plectostoma charasense

(Tomlin, 1948)

http://species-id.net/wiki/Plectostoma_charasense

**Figures 44.**
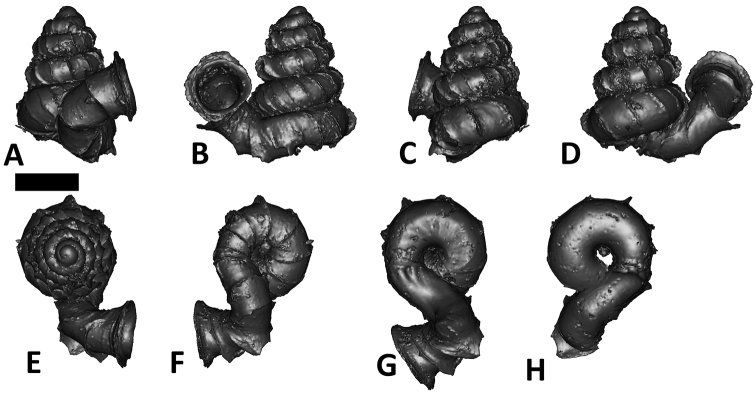
*Plectostoma charasense* (Tomlin, 1948) ZMA 162063. **A** frontal view **B** left lateral view **C** back view **D** right lateral view **E** top view **F** bottom view **G** parietal part of constriction inner whorl **H** basal part of constriction inner whorl. Scale bar = 1 mm (for **A–F**).

Figure 44 , Appendix 14

Opisthostoma charasense Tomlin, 1948: 225, Plate 2 - figure 4 (original description).
Opisthostoma charasense Tomlin, van Benthem Jutting (1952: 42).

#### Type material.

Holotype: BNHM 1948.10.2.2(1) (Seen).

#### Other examined materials.

ZMA 162063(>10), ZMA 162064(>10).

#### Diagnosis.

Shares with *Plectostoma kitteli*, *Plectostoma tohchinyawi*, and *Plectostoma klongsangensis* the general shell form, in terms of apex, spire, and tuba shape, but differs by lacking thick spiral lines and by having two basal constriction teeth.

#### Description.

**Apex.** Shape: distinctly convex.

**Spire.** Height: 1.9–2.0 mm. Width: 1.3–1.4 mm. Number of whorls: 4 1/8. Apical spire shape: oblong conical. Basal spire shape: conical. Whorl periphery: distinctly convex. Umbilicus: open.

**Constriction.** Parietal teeth: two. Basal teeth: two.

**Tuba.** Coiling direction: type 2 and aperture visible from right lateral view. Tuba whorl length in proportion to spire last whorl: ca. 7/8. Proportion of tuba that attaches to spire: less than 1/2.

**Aperture and peristome.** Peristome: double peristomes. Outer peristome shape: same as inner peristome and uniformly projected all around, except the posterior part.

**Spiral lines.** Thick lines: absent. Thin lines: present.

**Radial ribs.** Rib density: 4 ribs per mm. Rib intensity: thick. Shape: slightly curved to single-humped. Inclination: moderately prosoclin.

#### Distribution.

Type locality. Gua Charas, limestone hill near Sungai Lembing in the state of Pahang, Malaysia (3°54'27"N, 103°8'47"E).

Distribution range. Until now, this species has been recorded from two neighbouring limestone hills, namely, Gua Charas and Bukit Panching. However, the whole hill of Bukit Panching has been lost to quarrying (Figure 18B).

#### Conservation status.

Critically Endangered (B2ab(iii)+C2a(i) ver. 10.1). Gua Charas might support a viable population. However, the hill is currently surrounded by oil palm plantation with a very narrow forested buffer zone. No living individuals or fresh dead shells were found at Gua Charas after exhaustive search during several sampling trips in 2010 and 2011.

#### Discussion.

The taxonomic status of this species is stable. Although the two neighbouring species, *Plectostoma tohchinyawi* and *Plectostoma praeco*, are similar to *Plectostoma charasense*, there are a few key shell characters that separate *Plectostoma charasense* from the others. See discussion under *Plectostoma tohchinyawi* for more discussion about the biogeography of this species.

### Plectostoma tohchinyawi sp. n.

http://zoobank.org/1D7B3A14-7D4D-42A1-B4BB-E12A71C560B5

http://species-id.net/wiki/Plectostoma_tohchinyawi

Figure 17S , 45 , Appendix 14

#### Type material.

Holotype: BOR 5649(1). Paratypes: BOR 5533(>10), BOR 5534(>10), BOR 5535(>10), V 8811(>25).

#### Diagnosis.

Shares with *Plectostoma charasense*, *Plectostoma kitteli*, and *Plectostoma klongsangensis* the general shell form, in terms of apex, spire, and tuba shape, but differs by having both thin and thick spiral lines, and the left lateral side of outer peristome projected not more than twice the width of the right lateral side of outer peristome.

#### Etymology.

This species is named after Dato’ Toh Chin Yaw, who was the Chairman of Industry, Trade and Environment Committee in the Terengganu State Government between 2008 and 2013. It is rare to find politicians working closely with the public for wildlife conservation. During his time in office, Dato’ Toh was tireless in his efforts to promote and preserve Terengganu’s natural heritage. One of his most influential decisions was getting the State to ban the hunting of threatened flying foxes (*Pteropus vampyrus*). Before he left office, he was working with scientists to gazette the Kenyir Wildlife Corridor as a protected area and was helping to secure funds to improve anti-poaching efforts in that area.

#### Description.

**Apex.** Shape: distinctly convex.

**Spire.** Height: 2.1–2.4 mm. Width: 1.3–1.5 mm. Number of whorls: 4 7/8–5 3/8. Apical spire shape: oblong conical. Basal spire shape: conical. Whorl periphery: distinctly convex. Umbilicus: open.

**Constriction.** Parietal teeth: two. Basal teeth: none.

**Tuba.** Coiling direction: type 2 and aperture visible in right lateral view. Tuba whorl length in proportion to spire last whorl: ca. 7/8–8/8. Proportion of tuba that attaches to spire: less than 1/2.

**Aperture and peristome.** Peristome: double peristomes. Outer peristome shape: similar to inner peristome, projected all around, except the posterior part, where the two lateral sides are distinctly more projected than the anterior side.

**Spiral lines.** Thick lines: present. Thin lines: present.

**Radial ribs.** Rib density: 4–6 ribs per mm. Rib intensity: thick. Shape: slightly curved to single-humped. Inclination: prosoclin.

**Figures 45.**
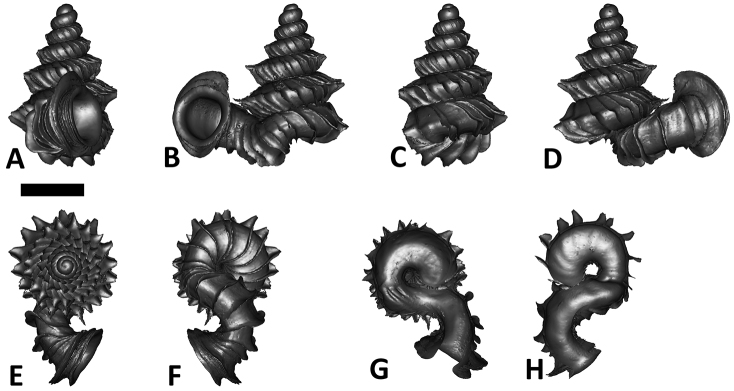
*Plectostoma tohchinyawi* sp. n. BOR 5649. **A** frontal view **B** left lateral view **C** back view **D** right lateral view **E** top view **F** bottom view **G** parietal part of constriction inner whorl **H** basal part of constriction inner whorl. Scale bar = 1 mm (for **A–F**).

#### Distribution.

Type locality. Gua Bewah, Tasik Kenyir (4°51'3"N, 102°43'21"E).

Distribution range. To date, this species is only known to occur at two neighbouring limestone outcrops, namely, Gua Bewah and Gua Taat at the Southern part of Tasik Kenyir (Figure 18B).

#### Conservation status.

Least concern. The only two limestone outcrops where this species was found are partially submerged in Southeast Asia’s largest man-made lake, Tasik Kenyir. Nonetheless, there is good forest cover around and on the limestone outcrops. Furthermore, these two hills are located in the Taman Negara Pahang, which is a prioritised protected area.

#### Discussion.

From a conchological point of view, this species is related to *Plectostoma charasense*. Both are thought to be affiliated with the *Plectostoma* species from North Borneo. *Plectostoma tohchinyawi* has a high conical spire, thick and projected radial ribs, and a long detached tuba. These are the typical characteristics for dozens of *Plectostoma* species in North Borneo. In addition, *Plectostoma tohchinyawi* and *Plectostoma charasense* live on the five lenticular limestone outcrops that are the easternmost outcrops of the Malay Peninsula, thus closest geographically to Borneo (Figure 18B).

### Plectostoma annandalei

(Sykes, 1903)

http://species-id.net/wiki/Plectostoma_annandalei

Opisthostoma annandalei Sykes, 1903: 198, Plate 20 - figures 4 & 5 (original description).
Opisthostoma annandalei Sykes, Laidlaw (1928: 36).
Opisthostoma annandalei Sykes, van Benthem Jutting (1952: 41).

#### Type material.

Not seen.

#### Diagnosis.

Shares with *Plectostoma laidlawi*, *Plectostoma tenggekensis*, and *Plectostoma praeco* the general shell form, in terms of spire and tuba shape, but differs by having slightly convex whorl periphery and straight ribs.

#### Description

(estimated from figure in Sykes 1903). **Apex.** Shape: moderately convex.

**Spire.** Height: 2 mm. Width: 1.3 mm. Number of whorls: 4 1/2. Apical spire shape: depressed conical. Basal spire shape: conical. Whorl periphery: moderately convex. Umbilicus: Open.

**Constriction.** Unknown.

**Tuba.** Coiling direction: type 2 and aperture visible in right lateral view. Tuba whorl length in proportion to spire last whorl: approximately the same as the spire’s last whorl length. Proportion of tuba that attaches to spire: less than 1/2.

**Aperture and peristome.** Peristome: double peristomes. Outer peristome shape: same as inner peristome and uniformly projected all around, except the posterior part.

**Spiral lines.** Unknown

**Radial ribs.** Rib density: 5 ribs per mm. Rib intensity: unknown. Shape: straight. Inclination: orthoclin.

#### Distribution.

Type locality. This species is only known from its type locality, Jalor (Biserat). The exact location was not described in the original publication of this species. From the collectors’ report (Annandale and Robinson 1913), we estimated the location and name of the limestone hill from a map in the report. This hill was named Bukit Bayu. Later, we estimated its coordinates from Google Earth (6°16'48"N, 101°13'35"E) (Figure 18B).

Distribution range. Unknown.

#### Conservation status.

Data Deficient.

#### Discussion.

This species has not been seen or collected after the original description. The type specimens cannot be located. Sykes (1903) mentioned that it is similar to *Plectostoma laidlawi* but he did not explain explicitly in what way. This is not unexpected, as *Plectostoma laidlawi* was the only one *Plectostoma* known from Peninsular Malaysia at the time, although more than 20 *Plectostoma* species had already been described from Borneo. Thus, one can assume that the statement made by Sykes was based on the geographical proximity.

Interestingly, *Plectostoma panhai* was described from a limestone hill located just 8 km from the location where *Plectostoma annandalei* was found. In addition to the geographical proximity, the two neighbouring species are similar in several shell characteristics. The shell spires are very similar in terms of number of whorls, overall shape and size. On the other hand, the main difference between these two species is the tuba coiling direction (type 1 vs. type 2).

### Plectostoma praeco

(van Benthem Jutting, 1961)

http://species-id.net/wiki/Plectostoma_praeco

**Figures 46.**
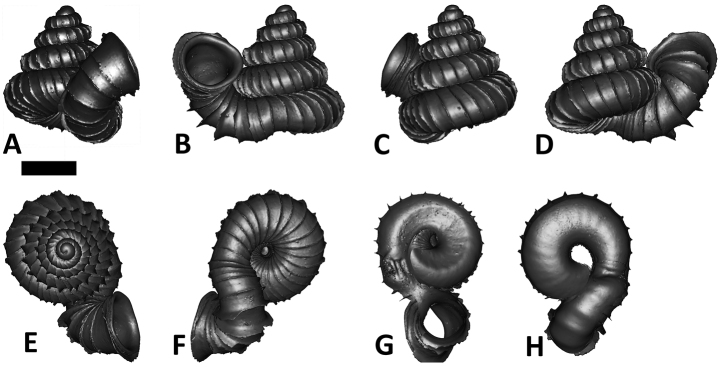
*Plectostoma praeco* (van Benthem Jutting, 1961) ZMA 162150. **A** frontal view **B** left lateral view **C** back view **D** right lateral view **E** top view **F** bottom view **G** parietal part of constriction inner whorl **H** basal part of constriction inner whorl. Scale bar = 1 mm (for **A–F**).

Figure 46 , Appendix 14

Opisthostoma praeco van Benthem Jutting, 1961: 39, Plate 2 - figure 5 (original description).

#### Type material.

Holotype: ZMA 136034(1) (Seen). Paratypes: ZMA 136041(>50) (Seen), ZMA 136042(>25) (Seen).

#### Other examined materials.

ZMA 162150(>100), ZMA 162151(>100), ZMA 162152(>100), ZMA 162153(>10).

#### Diagnosis.

Shares with *Plectostoma laidlawi*, *Plectostoma tenggekensis*, and *Plectostoma annandalei* the general shell form, in terms of spire and tuba shape, but differs by having slightly curved ribs.

#### Description.

**Apex.** Shape: distinctly convex.

**Spire.** Height: 2.0–2.2 mm. Width: 1.8–2.0 mm. Number of whorls: 4 1/4–4 5/8. Apical spire shape: depressed conical. Basal spire shape: conical. Whorl periphery: moderately to distinctly convex. Umbilicus: open.

**Constriction.** Parietal teeth: two. Basal teeth: none.

**Tuba.** Coiling direction: type 2 and aperture visible in right lateral view. Tuba whorl length in proportion to spire last whorl: *ca.* 5/8–7/8. Proportion of tuba that attaches to spire: more than 1/3 but less than 1/2.

**Aperture and peristome.** Peristome: double peristomes. Outer peristome shape: similar to inner peristome, projected all around, except the posterior part, where the left lateral sides are slightly more projected than the anterior and right lateral side.

**Spiral lines.** Thick lines: absent. Thin lines: present.

**Radial ribs.** Rib density: 6–7 ribs per mm. Rib intensity: thin. Shape: slightly curved. Inclination: moderately prosoclin.

#### Distribution.

Type locality. Batu Che Derani, Taman Negara Pahang (4°35'57"N, 102°25'57"E).

Distribution range. *Plectostoma praeco* has been recorded from four other hills at upper Sungai Keniyam Kecil, which is in the vicinity of the type locality (Figure 18B).

#### Conservation status.

Least concern. All the limestone hills where *Plectostoma praeco* occurs, are located in the National Park.

#### Discussion.

There is no genetic information for *Plectostoma praeco*. Morphological similarity suggests that *Plectostoma praeco* and *Plectostoma tenggekensis* are closely related. Each of the two species has a narrow distribution range and they are separated from each other by at least 50 km.

### Plectostoma tenggekensis sp. n.

http://zoobank.org/38A095DD-552F-4F77-8774-1E834531352C

http://species-id.net/wiki/Plectostoma_tenggekensis

**Figures 47.**
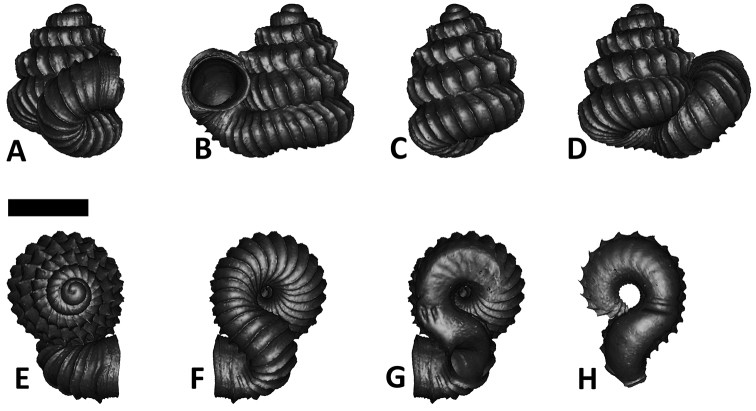
*Plectostoma tenggekensis* sp. n. BOR 5650. **A** frontal view **B** left lateral view **C** back view **D** right lateral view **E** top view **F** bottom view **G** parietal part of constriction inner whorl **H** basal part of constriction inner whorl. Scale bar = 1 mm (for **A–F**).

Figure 47 , Appendix 14

#### Type material.

Holotype: BOR 5650(1). Paratypes: V 13554(5), BOR 444(4), BOR 5596(>10).

#### Diagnosis.

Shares with *Plectostoma laidlawi*, *Plectostoma annandalei*, and *Plectostoma praeco* the general shell from, in terms of spire and tuba shape, but differs by having single-humped shaped ribs.

#### Etymology.

This species is named after its type locality–Bukit Tenggek, Kuantan, Pahang.

#### Description.

**Apex.** Shape: moderately convex.

**Spire.** Height: 1.6–1.7 mm. Width: 1.3–1.4 mm. Number of whorls: 4–4 1/4. Apical spire shape: depressed. Basal spire shape: conical. Whorl periphery: moderately convex. Umbilicus: open.

**Constriction.** Parietal teeth: two. Basal teeth: none.

**Tuba.** Coiling direction: type 2 and aperture visible in right lateral view. Tuba whorl length in proportion to spire last whorl: *ca.* 5/8 of the spire last whorl length. Proportion of tuba that attaches to spire: less than 1/3.

**Aperture and peristome.** Peristome: double peristomes. Outer peristome shape: similar to inner peristome, projected all around, except the posterior part, where the left lateral sides are slightly more projected than the anterior and right lateral side.

**Spiral lines.** Thick lines: absent. Thin lines: present.

**Radial ribs.** Rib density: 6–7 ribs per mm. Rib intensity: thin. Shape: slightly curved. Inclination: moderately prosoclin.

#### Distribution.

Type locality. Bukit Tenggek, Pahang (4°0'51"N, 103°9'34"E).

Distribution range. Endemic to type locality (Figure 18B).

#### Conservation status.

Critically Endangered (B2ab(iii)+C2a(i) ver. 10.1). The entire Bukit Tenggek will be gone by 2014 because of quarrying activity.

#### Discussion.

See discussion under *Plectostoma praeco*.

### Plectostoma laidlawi

(Sykes, 1902)

http://species-id.net/wiki/Plectostoma_laidlawi

**Figures 48.**
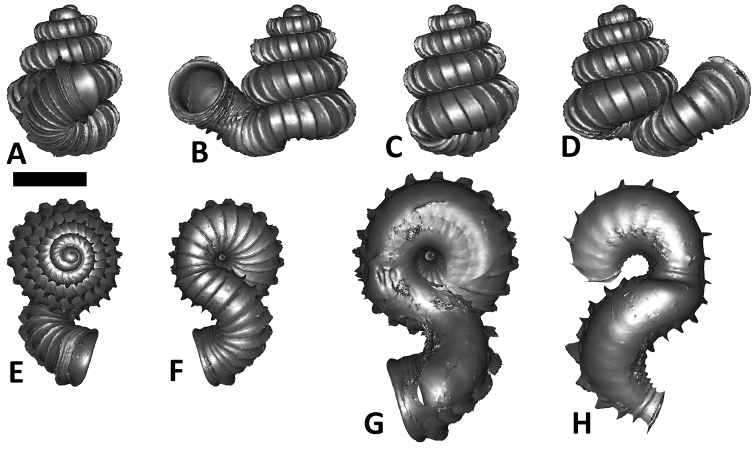
*Plectostoma laidlawi* (Sykes, 1902) (Form BOR 5510). **A** frontal view **B** left lateral view **C** back view **D** right lateral view **E** top view **F** bottom view **G** parietal part of constriction inner whorl **H** basal part of constriction inner whorl. Scale bar = 1 mm (for **A–F**).

**Figures 49.**
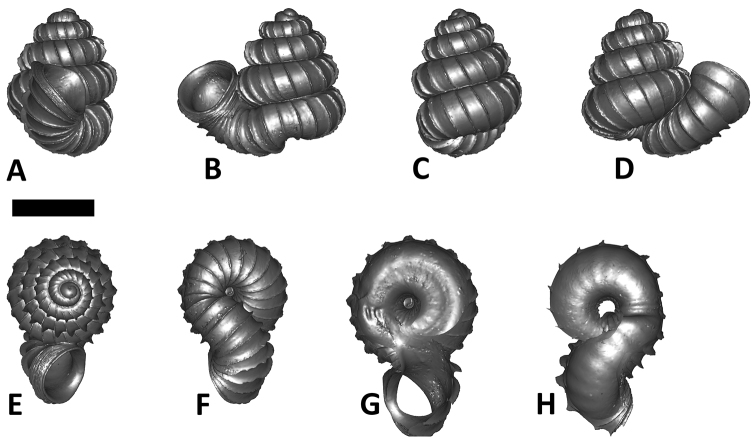
*Plectostoma laidlawi* (Sykes, 1902) (Form BOR 5571). **A** frontal view **B** leftlateral view **C** back view **D** right lateral view **E** top view **F** bottom view **G** parietal part of constriction inner whorl **H** basal part of constriction inner whorl. Scale bar = 1 mm (for **A–F**).

Figures 17O , 48 , 49 , Appendix 15

Opisthostoma laidlawi Sykes, 1902a: 22 (original description).
Opisthostoma laidlawi Sykes, Sykes (1902b: Plate 3 - figures 13 & 14).
Opisthostoma laidlawi Sykes, von Moellendorff (1902: 143).
Opisthostoma laidlawi Sykes, Laidlaw (1928: 36).
Opisthostoma laidlawi Sykes, van Benthem Jutting (1952: 41).
Opisthostoma laidlawi Sykes, Ito et al. (2009: 58).
Opisthostoma laidlawi Sykes, Sasaki (2010: 126, figure 2.7B).

#### Type material.

Holotype: Not Seen. Paratype: ZMA 136014(1) (Seen).

#### Other examined materials.

BMNH C_ACC1825(1), BOR 5510(>25), BOR 5571(2), RMNH 156268(1), ZMA 162095(2), ZMA 162097(>25), ZMA 162099(>10), ZMA 162100(>25), ZMA 162096(1), V5558(1), V 7878(3), V 8345(>100), V 8476(>25), V 8670(>25), V 8693(>50), V 8950(>50), V 9044(>25), V 9109(>25), V 9125(>25), V 9359(6).

#### Diagnosis.

Shares with *Plectostoma annandalei*, *Plectostoma tenggekensis*, and *Plectostoma praeco* the general shell form in terms of spire and tuba shape, but differs by having a distinctly convex whorl periphery and straight ribs.

#### Description.

**Apex.** Shape: moderately to distinctly convex.

**Spire.** Height: 1.6–2.3 mm. Width: 1.3–1.8 mm. Number of whorls: 3 5/8–4. Apical spire shape: depressed conical. Basal spire shape: conical to ovoid. Whorl periphery: distinctly convex. Umbilicus: open.

**Constriction.** Parietal teeth: two. Basal teeth: none.

**Tuba.** Coiling direction: type 2 and aperture visible in right lateral view. Tuba whorl length in proportion to spire last whorl: *ca.* 5/8–7/8. Proportion of tuba that attaches to spire: varies from completely attached to as much as half of the tuba detached from spire.

**Aperture and peristome.** Peristome: double peristomes. Outer peristome shape: similar to inner peristome, projected all around, except the posterior part, where the two lateral sides are slightly more projected than the anterior side or left lateral sides slightly more projected than the anterior and right lateral side.

**Spiral lines.** Thick lines: present. Thin lines: present.

**Radial ribs.** Rib density: 5–6 ribs per mm. Rib intensity: thin. Shape: straight to slightly curved. Inclination: moderately prosoclin.

#### Distribution.

Type locality. “Kelantan, Malay Peninsula”, collected by J. Waterstradt (Sykes 1902). According to Waterstradt’s (1902) itinerary, he visited one of the limestone hills at Kampung Pulai. The Pulai Princess cave (4°47'38"N, 101°56'31"E) fits perfectly with Waterstradt’s descriptions on the local population, temples and hill’s topography; he also described that he and his collectors sampled shells here (Waterstradt 1902: 9–10). We conclude that Pulai Princess Cave must be the type locality of *Plectostoma laidlawi*. However, a recent survey at the highly degraded Bukit Pulai Princess cave failed to retrieve any shells of *Plectostoma*.

Distribution range. This species occurs on the limestone hills along Berok River and Nenggiri River (Figure 18B).

#### Conservation status.

Vulnerable (D2 ver. 10.1). Living populations were present on only two limestone hills during a survey in May 2011. All the limestone hills for which this species is known are surrounded by degraded forest and oil palm plantation.

#### Discussion.

The morphology of this species is quite variable in shell size and degree of attachment of the tuba to the spire. The distribution range partially overlaps with that of *Plectostoma davisoni*. It is possible that the species actually consists of two or more cryptic species, and thus more genetic data are needed.

## Author contributions

Conceived and designed the experiments: Thor-Seng Liew. Performed the experiments: Thor-Seng Liew. Analyzed the data: Thor-Seng Liew, Jaap Jan Vermeulen. Contributed reagents/materials/analysis tools: Thor-Seng Liew, Jaap Jan Vermeulen, Mohammad Effendi bin Marzuki, Menno Schilthuizen. Wrote the paper: Thor-Seng Liew, Jaap Jan Vermeulen, Menno Schilthuizen, Mohammad Effendi bin Marzuki.

## Supplementary Material

XML Treatment for Plectostoma

XML Treatment for Plectostoma dindingensis

XML Treatment for Plectostoma mengaburensis

XML Treatment for Plectostoma sinyumensis

XML Treatment for Plectostoma umbilicatum

XML Treatment for Plectostoma siphonostomum

XML Treatment for Plectostoma panhai

XML Treatment for Plectostoma christae

XML Treatment for Plectostoma crassipupa

XML Treatment for Plectostoma tonkinianum

XML Treatment for Plectostoma whitteni

XML Treatment for Plectostoma sciaphilum

XML Treatment for Plectostoma senex

XML Treatment for Plectostoma turriforme

XML Treatment for Plectostoma palinhelix

XML Treatment for Plectostoma retrovertens

XML Treatment for Plectostoma ikanensis

XML Treatment for Plectostoma kayiani

XML Treatment for Plectostoma davisoni

XML Treatment for Plectostoma relauensis

XML Treatment for Plectostoma kakiense

XML Treatment for Plectostoma kubuensis

XML Treatment for Plectostoma laemodes

XML Treatment for Plectostoma salpidomon

XML Treatment for Plectostoma kitteli

XML Treatment for Plectostoma klongsangensis

XML Treatment for Plectostoma charasense

XML Treatment for Plectostoma tohchinyawi

XML Treatment for Plectostoma annandalei

XML Treatment for Plectostoma praeco

XML Treatment for Plectostoma tenggekensis

XML Treatment for Plectostoma laidlawi

## Appendix

(http://dx.doi.org/10.6084/m9.figshare.830412)

**Appendix 1.** Video tutorials of cybertaxonomy procedures.

**Appendix 2.** Details for collection data.

**Appendix 3.** Spiral striation profile on the shell spire of 29 *Plectostoma* species.

**Appendix 4.** Radial rib spacing profile on the shell spire of 29 *Plectostoma* species.

**Appendix 5.** A KML file consisting of collection data, image links and distribution data.

**Appendix 6.** A Blender file consisting of 17 shell 3D models of 2 species, Part 1 of 10.

*Plectostoma christae* (Maassen, 2001)

- V 12702 (1), BOR 5505 (1), BOR 5506 (2), BOR 5572(1), V 9285 (2).

*Plectostoma crassipupa* (van Benthem Jutting, 1952)

- BOR 5512 (1), V 8392 (2), V 9097 (3), V 9326 (2), V 9353 (2).

**Appendix 7.** A Blender file consisting of 18 shell 3D models of 6 species, Part 2 of 10.

*Plectostoma dindingensis* sp. n.

- BOR 5612 (3), BOR 5642 (1).

*Plectostoma mengaburensis* sp. n.

- BOR 5643 (1), V 8822 (1).

*Plectostoma panhai* (Maassen, 2001)

- ZMA 138438 (1), RMNH 81810 (2).

*Plectostoma sciaphilum* (van Benthem Jutting, 1952)

- ZMA 136050 (3).

*Plectostoma senex* (van Benthem Jutting, 1952)

- BOR 5628 (2), BOR 5603 (1).

*Plectostoma sinyumensis* (Maassen, 2001)

- BOR 5623 (2), BOR 5537 (1).

**Appendix 8.** A Blender file consisting of 14 shell 3D models of 5 species, Part 3 of 10.

*Plectostoma siphonostomum* (van Benthem Jutting, 1952)

- BOR 5521 (1), BOR 5557 (1).

*Plectostoma whitteni* sp. n.

- BOR 5644 (1), V 8885 (2).

*Plectostoma tonkinianum* (Dautzenberg & Fischer, 1905)

- V 11502 (1), V 9940 (1).

*Plectostoma turriforme* (van Benthem Jutting, 1952)

- BOR 5509 (1), ZMA 136069 (2).

*Plectostoma umbilicatum* (van Benthem Jutting, 1952)

- BOR 5503 (2), BOR 5625 (2).

**Appendix 9.** A Blender file consisting of 19 shell 3D models of 1 species, Part 4 of 10.

*Plectostoma davisoni* sp. n.

- BOR 5626 (2), BOR 5641 (2), BOR 5646 (1), V 14242 (1), V 8265 (3), V 8652 (3), V 9206 (1), V 9243 (2), ZMA 162071 (4).

**Appendix 10.** A Blender file consisting of 19 shell 3D models of 1 species, Part 5 of 10.

*Plectostoma davisoni* sp. n.

- V 9340 (2), ZMA 162069 (5), ZMA 162070 (3), ZMA 162146 (3), ZMA 162147 (6).

**Appendix 11.** A Blender file consisting of 18 shell 3D models of 4 species, Part 6 of 10.

*Plectostoma kayiani* sp. n.

- RMNH 330803 (1), V 8883 (2)

*Plectostoma ikanensis* sp. n. Form BOR 5507

- BOR 5645 (1), V 9320 (4), V9446 (4).

*Plectostoma kakiense* (Tomlin, 1948)

- BOR 5516 (1), BOR 5517 (2).

*Plectostoma kitteli* (Maassen, 2002)

- RMNH 92956 (2), V 12697 (1).

**Appendix 12.** A Blender file consisting of 14 shell 3D models of 3 species, Part 7 of 10.

*Plectostoma kubuensis* sp. n.

- BOR 5518 (1), BOR 5519 (3), BOR 5648 (1).

*Plectostoma laemodes* (van Benthem Jutting, 1961)

- RMNH 156267 (3), V 8490 (2), ZMA 136011 (1).

*Plectostoma palinhelix* (van Benthem Jutting, 1952)

- BOR 5520 (1), V 5104 (2).

**Appendix 13.** A Blender file consisting of 11 shell 3D models of 3 species, Part 8 of 10.

*Plectostoma relauensis* sp. n.

- BOR 5511 (2), BOR 5647 (1), V 8196 (2).

*Plectostoma retrovertens* (Tomlin, 1938)

- V 5142 (2), BOR 5559 (1).

*Plectostoma salpidomon* (van Benthem Jutting, 1952)

- BOR 5539 (1), V 8658 (2).

**Appendix 14.** A Blender file consisting of 13 shell 3D models of 5 species, Part 9 of 10.

*Plectostoma tohchinyawi* sp. n.

- BOR 5533 (2), BOR 5649 (1), V 8811 (2).

*Plectostoma charasense* (Tomlin, 1948)

- ZMA 162063 (1), ZMA 162064 (2).

*Plectostoma praeco* van Benthem Jutting, 1961

- ZMA 162150 (1), ZMA 162151 (1), ZMA 162152 (1).

*Plectostoma ikanensis* sp. n. Form BOR 5504

- BOR 5504 (1).

*Plectostoma tenggekensis* sp. n.

- BOR 5596 (1), BOR 5649 (1).

**Appendix 15.** A Blender file consisting of 10 shell 3D models of 1 species, Part 10 of 10.

*Plectostoma laidlawi* (Sykes, 1902) Form BOR 5510

- BOR 5510 (1), V 5558 (1), ZMA 136014 (1).

*Plectostoma laidlawi* (Sykes, 1902) Form BOR 5571

- BOR 5571 (1), V 7878 (2), ZMA 162099 (4).

**Appendix 16.** 51 COI sequences for 19 *Plectostoma* species in FASTA format. Each sequence is labeled with BOLD process ID, GenBank accession no, sample ID and species name.

**Appendix 17.** DNA sequence alignment for phylogenetic analysis.

**Appendix 18.** Shell colouration of *Opisthostoma* species.
